# Specialization patterns in symbiotic associations: A community perspective over spatial scales

**DOI:** 10.1002/ece3.10296

**Published:** 2023-07-10

**Authors:** Clara Rodríguez‐Arribas, Isabel Martínez, Gregorio Aragón, Carlos Zamorano‐Elgueta, Lohengrin Cavieres, María Prieto

**Affiliations:** ^1^ Área de Biodiversidad y Conservación, Research Group of “Ecología, sistemática y evolución de hongos y líquenes (ESEFUNLICH)”, Departamento de Biología, Geología, Física y Química Inorgánica, ESCET Universidad Rey Juan Carlos Móstoles Spain; ^2^ Universidad de Aysén Coyhaique Chile; ^3^ CR2‐Center for Climate and Resilience Research (CR)2 Santiago Chile; ^4^ Departamento de Botánica, Facultad de Ciencias Naturales y Oceanográficas Universidad de Concepción Concepción Chile

**Keywords:** lichen, mutualism, niche, specialization, symbiosis

## Abstract

Specialization, contextualized in a resource axis of an organism niche, is a core concept in ecology. In biotic interactions, specialization can be determined by the range of interacting partners. Evolutionary and ecological factors, in combination with the surveyed scale (spatial, temporal, biological, and/or taxonomic), influence the conception of specialization. This study aimed to assess the specialization patterns and drivers in the lichen symbiosis, considering the interaction between the principal fungus (mycobiont) and the associated *Nostoc* (cyanobiont), from a community perspective considering different spatial scales. Thus, we determined *Nostoc* phylogroup richness and composition of lichen communities in 11 *Nothofagus pumilio* forests across a wide latitudinal gradient in Chile. To measure specialization, cyanobiont richness, Simpson's and *d*′ indices were estimated for 37 mycobiont species in these communities. Potential drivers that might shape *Nostoc* composition and specialization measures along the environmental gradient were analysed. Limitations in lichen distributional ranges due to the availability of their cyanobionts were studied. Turnover patterns of cyanobionts were identified at multiple spatial scales. The results showed that environmental factors shaped the *Nostoc* composition of these communities, thus limiting cyanobiont availability to establish the symbiotic association. Besides, specialization changed with the spatial scale and with the metric considered. Cyanolichens were more specialized than cephalolichens when considering partner richness and Simpson's index, whereas the *d*′ index was mostly explained by mycobiont identity. Little evidence of lichen distributional ranges due to the distribution of their cyanobionts was found. Thus, lichens with broad distributional ranges either associated with several cyanobionts or with widely distributed cyanobionts. Comparisons between local and regional scales showed a decreasing degree of specialization at larger scales due to an increase in cyanobiont richness. The results support the context dependency of specialization and how its consideration changes with the metric and the spatial scale considered. Subsequently, we suggest considering the entire community and widening the spatial scale studied as it is crucial to understand factors determining specialization.

## Graphical abstract

Specialization towards cyanbacteria in lichens is a context‐dependent concept, which changes with the metric and the spatial scale considered. As well, environmental factors shape *Nostoc* communities, determining the *Nostoc* phylogroups available in each forest to establish the symbiosis. However, *Nostoc* availability is not the only factor shaping mycobiont distributional ranges along a wide latitudinal gradient in Chile: (a) differences in *Nostoc* phylogroup composition in the different forests; (b) differences in specialization metrics (cyanobacteria richness, Simpson's and d' indices) according to how the interactions are established and the relative abundance of each *Nostoc* phylogroups; (c) wide distributional ranges are either reached by the replacement of partners or by the interaction with wide distributed partners and, (d) percentage found of the different turnover partners of partners within species between forests.

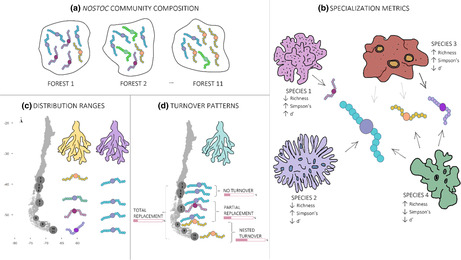

## 1 INTRODUCTION

The degree of specialization of the organisms to the abiotic and biotic conditions is a core concept in ecology. Specialization must be defined with reference to a particular axis of the requirement niche of an organism (i.e. resource), understanding the niche as the *n*‐dimensional hypervolume composed by *n* niche axes which refer to the set of biotic and abiotic requirements determining species or populations persistence (Carscadden et al., 2020; Hutchinson, 1957, 1978). Thus, organisms' requirements along their different niche axes define their specialization patterns (Forister et al., 2012; Futuyma & Moreno, 1988), being an organism considered a specialist when its requirements have a narrow amplitude, whereas generalists have a broad requirement breadth for a given resource or niche axis. The ecological relevance of specialization arises from a better performance of specialist organisms in their optimal habitats, at the expense of their performance in other habitats (Futuyma & Moreno, 1988). In addition, specialists could be negatively affected by disturbance, which may increase their probability of extinction (MacArthur, 1955). For this reason, understanding how specialization is driven and determining which species show a specialist pattern can help to find vulnerable species and to develop conservation strategies (Devictor et al., 2010).

One of the factors to consider specialization in biotic interactions refers to the interacting partners in both antagonistic and mutualistic systems. The specialization patterns range from reciprocal specialization, where the partners interact exclusively with each other, to generalization in which both partners interact with many others, with intermediate patterns in which both partners show different degrees of specialization (asymmetrical specialization). Remarkably, in biotic interactions, this degree of specialization is often considered from one partner (host) towards the other member of the interaction (e.g. Torres‐Martínez et al., 2021).

Within this context of biotic interactions, we focus on mutualist interactions. Mutualism is defined as “an interaction between species that is beneficial to both” (Boucher et al., 1982). This mutual benefit is due to the reciprocal exchange of resources or services (Peay, 2016) and may be affected by the degree of specialization, as a high specialization has been proposed to influence the selection of certain traits which increase the fitness of the association (Aigner, 2001; Irschick et al., 2005; Wilson & Thomson, 1996). Specialization in mutualistic interactions can be influenced by evolutionary and ecological factors (Armbruster, 2006; Herrera et al., 2019). From an evolutionary point of view, the unique and repeated interaction with high‐quality partners over long periods of time promotes coevolution and cospeciation, resulting in a high specialization (Armbruster, 2006; de Vienne et al., 2013; Forister et al., 2012; Herrera et al., 2019; Thompson, 1999, 2005). On the contrary, ecological factors might condition specialization by determining local partner availability due to abiotic filtering and/or biotic interactions (Iglesias‐Prieto et al., 2004; Rolshausen et al., 2020; Suz et al., 2014). The limitation of interactions due to partner availability is influenced by partner presence/absence, but also by its abundance, which promotes the selection of highly abundant compatible partners (Vázquez et al., 2005). In addition, considering the latitudinal diversity gradient (Hillebrand, 2004; Kinlock et al., 2018), the number of species is expected to be higher in the tropics, thus promoting an increase in specialization due to niche partitioning of interacting partners (Schluter, 2000). However, the variation of the degree of specialization along environmental gradients is still ambiguous. In this sense, environmental variables are expected to influence specialization patterns, as under extreme environmental conditions, generalist organisms have more chances to find high‐quality partners to deal with the extreme environmental conditions (Batstone et al., 2018), whereas specialist organisms are expected to be more efficient in habitats where environmental conditions are limited for most of the species (Carboni et al., 2016). Moreover, environmental gradients have been found to influence interactions not only in the level of specialization but also in the sign of the interaction, fluctuating between more positive interactions (i.e. mutualisms) under stressful or low‐resource conditions and negative (i.e. antagonisms) or neutral interactions under benign conditions (O'Brien et al., 2018), emphasizing the importance of the environments in which interactions take place.

The scale, considered as the spatial (i.e. local vs. regional), temporal (i.e. *t*_0_ to *t*_*N*_), biological (i.e. individuals, populations, species and communities), and/or taxonomic (i.e. species, genera, families) extent, plays an important role determining specialization in mutualist systems. How the scale affects specialization depends on the differences in the constancy of interactions between sites, time points, biological, and/or taxonomic entities (Hughes, 2000). Differences in specialization between different scales (e.g. local vs. regional and species vs. families) can be explained by different turnover scenarios as those proposed by Ventre Lespiaucq et al. (2021). These turnover scenarios are based on the analysis of the variation in species composition between different points of a defined scale (beta diversity).

Along with corals, mycorrhizae, legume‐rhizobium, pollination and seed‐dispersal interactions, lichenized fungi are one of the great examples of terrestrial mutualisms. An individual lichen is a complex ecosystem composed by the interaction of a primary fungus (the mycobiont host), one or more photosynthetic partners (photobionts) and an indeterminate number of other microscopic organisms (i.e. yeasts, bacteria; Hawksworth & Grube, 2020). In the symbiosis, the photobionts (algae or cyanobacteria) provide carbohydrates from photosynthesis, while the mycobiont (host) protects the photobionts from desiccation. Cyanobacterial partners (cyanobionts) also fix atmospheric dinitrogen (Nash, 1996).

Lichens associated with cyanobionts (cyanolichens and cephalolichens) represent nearly 10% of all known lichen‐symbiotic fungi (Jüriado et al., 2019; Rikkinen, 2015) and are ecologically important due to their nitrogen fixation properties (Asplund & Wardle, 2017; Ellis, 2012). Cyanobacteria is the only photobiont in bipartite cyanolichens, while cephalolichens (tripartite) have a green algae as the principal photobiont while cyanobacteria are held in modified internal or external structures known as cephalodia. The function of the cyanobiont in both cyanolichens and cephalolichens is different as in cyanolichens the cyanobacteria performs photosynthesis and contributes to nitrogen fixation whereas it is mostly in charge of nitrogen fixation in cephalolichens (Nash, 1996). The result of the interaction, the lichen thalli, is treated as an organism itself and is the entity affected by the specialization pattern acquired. The mycobiont is usually more specialized than the photobiont, possibly because the mycobiont is more dependent on the symbiosis, whereas the green algae and cyanobacteria can live independently (Magain et al., 2017). Thus, the degree of specialization is often considered from the perspective of the mycobiont (host). The advantage of the high specialization from the mycobiont to the photobiont can be seen as an adaptive process which allows an optimization of the fitness of the lichen thallus (i.e. abundance, growth rate, reproduction allocation and survival) to different local biotic and abiotic environmental variables as a result of the interaction with a well locally adapted photobiont to a set of biotic and abiotic variables (Magain et al., 2017; Rolshausen et al., 2018). On the contrary, generalization promotes the flexibility to associate with several photobionts which would translate into an improvement of the ecological tolerance of the lichen thallus (Batstone et al., 2018; Ertz et al., 2018).

Factors determining specialization patterns between mycobionts and photobionts are still unclear (Mark et al., 2020). Photobiont distribution and availability, lichen reproductive mode (linked to a vertical or a horizontal transmission of the photobiont), mycobiont identity, function of the cyanobiont in cyano‐ and cephalolichens, geography, or environmental variables have been shown to play major roles in shaping these patterns (Beck et al., 2002; Blaha et al., 2006; Fedrowitz et al., 2012; Fernández‐Mendoza et al., 2011; Kaasalainen et al., 2021; Lücking et al., 2009; Muggia et al., 2008; Otálora et al., 2010; Peksa & Škaloud, 2011; Piercey‐Normore & Deduke, 2011; Rikkinen, 2003; Yahr et al., 2006). However, most specialization studies of lichens are based on either small biological and/or spatial scales (e.g. Chagnon et al., 2018; Jüriado et al., 2019; Leavitt et al., 2015; Lu et al., 2018), and consequently, there is still a gap in knowledge of specialization patterns in a community context (i.e. considering all the coexisting species of lichens interacting with cyanobionts within forests) and along wide geographical scales.

In this study, we investigated specialization patterns of epiphytic communities of cyano‐ and cephalolichens growing in *Nothofagus pumilio* (Poepp. & Endl.) Krasser forests across a wide latitudinal gradient in Chile. Specifically, we aimed to: (1) analyse the availability and composition of *Nostoc* cyanobiont phylogroups establishing the lichen symbiosis across a broad latitudinal range, (2) correlate the distribution of these phylogroups with environmental variables that might shape the composition of *Nostoc* communities, (3) detect the specialization patterns of mycobionts towards their cyanobionts and the factors (environmental variables, reproductive mode, function of the cyanobiont and mycobiont identity) driving specialization, (4) determine whether generalist mycobionts result in lichens with wider distribution ranges and if these ranges are conditioned by the distribution ranges of their cyanobionts and (5) compare specialization patterns of mycobionts across spatial scales (local vs. regional). Based on previous knowledge, we propose four hypotheses: First, *Nostoc* availability increases with lower latitudes and *Nostoc* community composition is correlated with environmental factors that change with latitude. Second, environmental variables, reproductive mode, function of the cyanobiont, and/or mycobiont species identity are all correlated with specialization of the mycobiont. Third, generalized mycobionts are expected to result in lichens with wider distributional ranges, and specialized mycobionts to be conditioned by the distributional ranges of their cyanobionts. And fourth, specialization of the mycobiont decreases with increasing spatial scale, which could encompass several turnover scenarios.

## 2 MATERIALS AND METHODS

### 2.1 Sampling

**FIGURE 1:**
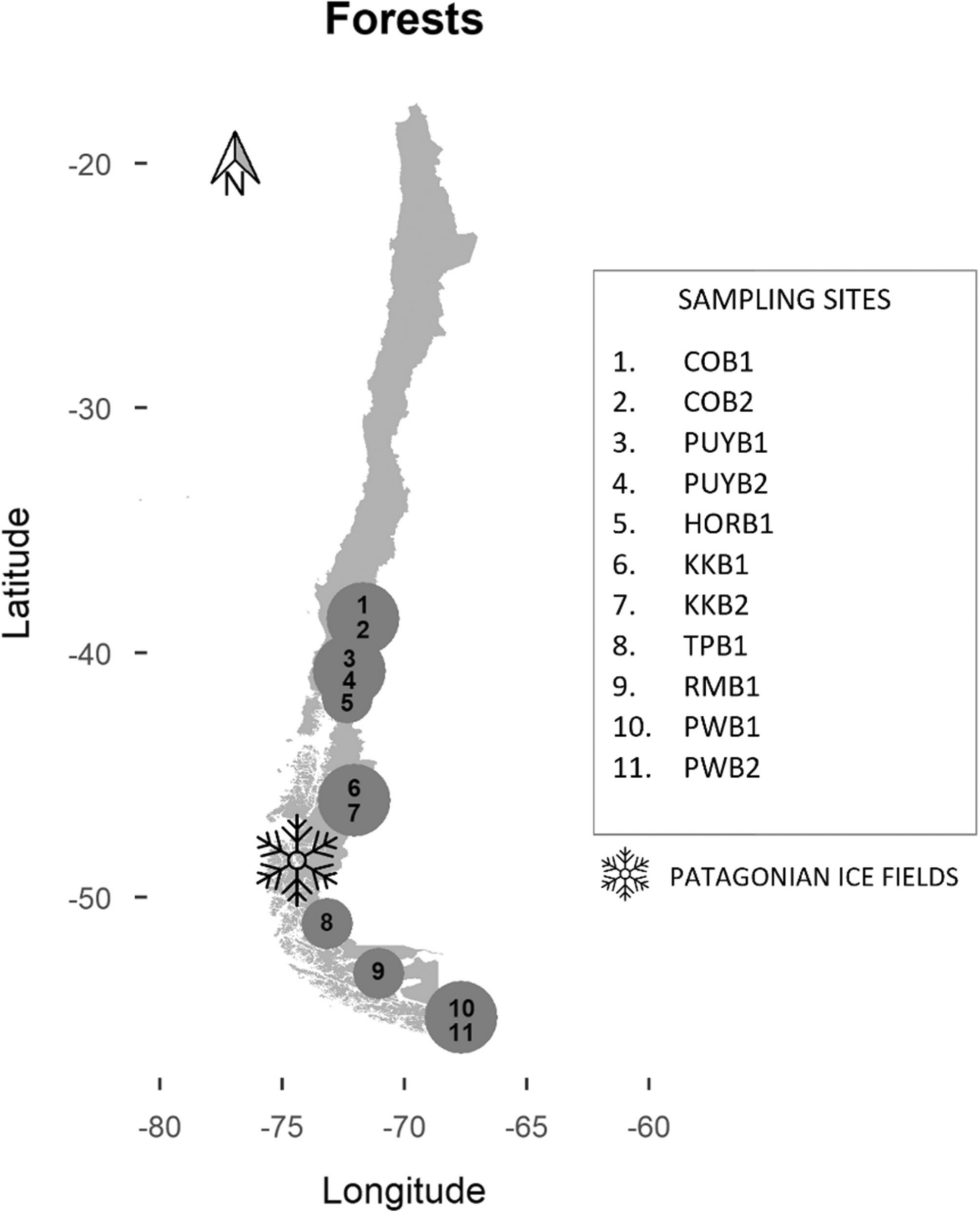
*Nothofagus pumilio* forests sampled along a latitudinal gradient. The size of the circles refers to the number of forests sampled in the same National Park or Reserve (bigger circles contain two forests named B1 and B2, and smaller circles represent one forest). Abbreviations of the areas: CO, Conguillío National Park; HOR, Hornopirén National Park; KK, Cerro Castillo National Park; PUY, Puyehue National Park; PW, Navarino Island; RM, Magallanes National Reserve; and TP, Torres del Paine National Park.

Between 2017 and 2018, 11 forest stands were sampled across a wide latitudinal gradient (from 38.63°S to 54.96°S), nine of them belonging to National Parks or Reserves (Figure 1). Forest stands were mostly formed by *N. pumilio* with over 65% of cover. Within each forest stand, we collected eight thalli of each species of cyanolichen and cephalolichen in different *N. pumilio* trees at a minimum distance to the forest edge of 100 m. Each thallus was considered as an individual. Samples were air‐dried and stored at −20°C. Forest structure and habitat quality‐related variables (elevation, orientation, slope, diameter at breast high (DBH) and canopy cover) were collected in situ in each forest. Elevation, orientation and slope (estimated by GPS, compass and clinometer, respectively) were measured at five locations in each forest, and DBH and canopy cover were quantified in 50 trees per forest. For the canopy cover, hemispherical photographs were taken with a horizontally levelled digital camera (Canon EOS 5D) aimed at the zenith, employing fish‐eye lens with a 180 field of view (SIGMA 8 mm F3.5 ex DG Fisheye). The photographs were analysed with Gap Light Analyzer v2.0 (GLA v2) (http://www.rem.sfu.ca/forestry/index.htm), which estimates the canopy openness as a percentage. Subsequently, we calculated the mean of these variables for each forest stand (Appendix S1).

A total of 1492 lichen thalli were collected belonging to 86 mycobiont species (53 cyanolichens and 33 cephalolichens). Lichen identification was based on morphological characters and followed Degelius (1974) for *Collema*, Jorgensen (2000, 2005) for *Fuscopannaria*, Galloway and Jørgensen (1995) for *Leptogium*, White and James (1988) for *Nephroma*, Passo et al. (2004), Elvebakk and Bjerke (2005), Passo and Calvelo (2006), Elvebakk (2007), Elvebakk et al. (2007), Passo and Calvelo (2011) and Elvebakk (2013) for *Pannaria*, Galloway (1985) for *Parmeliella* and *Psoroma*, Goward et al. (1995), Martínez Moreno (1999) and Vitikainen (1994) for *Peltigera*, Galloway (1992) and Lücking et al. (2017), for *Pseudocyphellaria s. lat*., Elvebakk et al. (2010) for *Psorophorus* and *Xanthopsoroma* and Galloway (1994) for *Sticta* (Appendix S2). Data from the reproductive mode (sexual and/or asexual) and the function of the cyanobiont stablishing the symbiosis (principal photobiont for cyanolichens or secondary photobiont for cephalolichens) were gathered observationally from the samples collected, as the reproductive mode could differ within the same species across the studied gradient.

### 2.2 Environmental variables

We selected 26 environmental variables related to climate, forest structure and habitat quality (Appendix S1). Climatic information was extracted from the CHELSA climate database (Karger et al., 2017), including information of 19 temperature and precipitation variables. We also considered the geographic variables of each forest (latitude and longitude). Variables related to forest structure and habitat quality comprise elevation, slope, orientation, canopy cover and tree DBH. To avoid multicollinearity, we selected variables not significantly correlated with each other (*r* > .7, *p* > .05), calculated with function *rcorr()* from package Hmisc (v.4.4‐1; Harrell & Dupont, 2018) and previously shown as being more ecologically relevant to lichen biology (Matos et al., 2015). The final variables included in the models were mean annual temperature (bio01), minimum temperature of the coldest month (bio06), precipitation of the driest month (bio14) and DBH.

### 2.3 *Nostoc* phylogroup delimitation

DNA from the cyanobionts was extracted using Chelex® 100 Chelating Resin (Bio‐Rad). Subsequently, *rbcLX* region was amplified with the primers CW and CX (Rudi et al., 1998) using the following program: 95°C 15 min; 35 cycles of 1 min at 95°C, 30 s at 54°C, 30 s at 72°C; and 10 min at 72°C. PCR products were sequenced at Macrogen Spain service (www.macrogen.com) using the same primers employed in the PCR. A total of 1120 cyanobacterial consensus sequences were obtained (Rodríguez‐Arribas et al., 2023).

The 1120 obtained sequences were edited and aligned using Geneious Prime 2021.0.1 software (https://www.geneious.com). Published sequences from Magain et al. (2017) were also added to the alignment. Ambiguous regions (i.e. two intergenic spacers) were delimited manually and excluded for the analysis using Aliview v. 1.26 (Larsson, 2014). Delimitation of *Nostoc* phylogroups was based on the ASAP method (Assemble Species by Automatic Partitioning, Puillandre et al., 2021) together with results (i.e. highly supported clades) obtained from the combined (Maximum likelihood and Bayesian) phylogenetic analysis and previous studies (Magain et al., 2017). ASAP analysis was carried out in the webserver (https://bioinfo.mnhn.fr/abi/public/asap/) applying the Jukes‐Cantor (JC69) model of substitution (split groups below 0.01 probability, keep 10 best scores and −1 as seed value). Partitions included in the 0.001–0.01 range of genetic distances were selected. Maximum likelihood (ML) analysis was conducted with RAxML v. 8.2.12 (Stamatakis, 2014) in the CIPRES Science Gateway Portal (Miller et al., 2010) with 1000 bootstrap iterations. The Bayesian analysis was run in MrBayes 3.2.7a (Huelsenbeck & Ronquist, 2001) in Agapita server (URJC, Biodiversity and Conservation Area) for 50 million generations with two runs and four chains, sampling every 1000 generations with a burning of the 25% and the GTR + I + G substitution model (Rodríguez et al., 1990).

### 2.4 Availability and composition of *Nostoc* phylogroups along the latitudinal gradient

We analyse *Nostoc* availability along the whole latitudinal gradient sampled in order to detect varying *Nostoc* diversity (i.e. richness) in the different forests with a linear model, using function *lm()* from package stats (R Core Team, 2021).

To predict which of the selected environmental variables might have influenced *Nostoc* composition, a redundancy analysis (RDA) was performed using symbiotic *Nostoc* abundances per forest and the *rda()* function in package vegan (v.2.5‐7; Oksanen et al., 2020). The variation explained by each variable was estimated with the *varpart()* function from the same package and a Venn diagram.

### 2.5 Specialization measures

We selected those mycobiont species with at least 10 sequences along the latitudinal gradient to ensure a minimum number of specimens for the following analysis. Specialization measures were calculated at the forest level for every mycobiont species from the previously selected with a minimum of four *rbcLX* sequences in that forest. In total, 37 mycobiont species met these requirements in at least one of the forests.

#### 2.5.1 Number of interacting partners and sampling efficiency

Cyanobiont richness was calculated per mycobiont species per forest as the number of interacting partners with which a mycobiont species establishes in each of the forests for the selected 37 species.

Accumulation curves were estimated per mycobiont species along the latitudinal gradient (for the selected 37 species) and per forest (including all species found in each forest) to determine the sampling efficiency (i.e. if *Nostoc* diversity within species and within forest was well determined) with the function *specaccum()* from the vegan package (v.2.5‐7; Oksanen et al., 2020). The number of potential *Nostoc* phylogroups was estimated with the Chao 1 equation using *estimateR()*. Differences between the observed cyanobiont richness and the Chao1 estimation within each mycobiont and within each forest were analysed using a *t*‐test with function *t‐test()* from the package stats (R Core Team, 2021).

#### 2.5.2 Indices: Simpson and *d*′

Two different indices were used to quantify specialization at the forest level for each of the previously selected 37 mycobiont species with at least four sequences in the forest.

Simpson's index (Simpson, 1949) was calculated as:

$$D_{i}=\sum_{j=1}^{N}p_{ij}^{2}$$

where *N* is the number of *Nostoc* phylogroups associated with mycobiont species *i*, and *p_ij_* is the frequency of association of species *i* with each phylogroup *j*. The Simpson's index considers the richness and the evenness of the partners and accentuates the contribution of common versus rare species (Magurran, 1988; Sahli & Conner, 2006), emphasizing the selectivity or preference towards certain cyanobionts.

*d*′‐index (Blüthgen et al., 2006) was calculated using the function *dfun()* from the bipartite package v.2.16 in R (Dormann et al., 2021) as:

$$d_{i}=\sum_{j=1}^{c}\left(p_{ij}\ln\frac{p_{ij}}{q_{j}}\right)$$

where c is the total number of interacting phylogroup species, $p_{ij}$ is the distribution of the interactions of species *i* with each partner *j*, and *q*_*j*_ is the relative availability of each Nostoc partner *j*. This equation is normalized to result in the *d*′‐index (see Blüthgen et al., 2006 for further details). The *d*′ index is a niche breadth index which consists of a standardized form of the Kullback–Leibler distance and considers the proportion of interactions with the different partners in relation to their relative abundance in the community. It quantifies whether a species should be considered as more opportunistic because it uses the resources in the same proportion as they are in the environment (low specialization), in comparison with a species that uses preferentially rare resources (high specialization; e.g. Blüthgen et al., 2007).

Both indices (Simpson's and *d*′‐index) range from zero to one. A Simpson's index equal to one means that a mycobiont species will always be associated with the same *Nostoc* phylogroup, while a mycobiont associating with different phylogroups in different proportions will have lower values. On the contrary, a *d*′‐index of one is achieved when reciprocal specialization is reached (i.e. both myco‐ and cyanobiont only associate between them). However, values close to zero of *d*′‐index are reached when a mycobiont is associated with *Nostoc* phylogroups in the same proportion as their availability in the forest, suggesting that the interaction is opportunistic rather than specialized (Blüthgen et al., 2006).

#### 2.5.3 Generalized linear mixed models

Factors associated with the number of interacting partners (cyanobiont richness), and both Simpson's and *d*′ indices for each mycobiont species per forest were analysed using generalized linear mixed models (GLMMs). The environmental variables selected (DBH, bio01, bio06 and bio14), the reproductive mode observed (sexual, asexual, both, or none) and the function of the cyanobiont (primary photobiont for cyanolichens or secondary photobiont for cephalolichens) were included as fixed effects, while forests and mycobiont species identity were included as random effects. We performed GLMMs with Poisson distribution for cyanobiont richness and Gaussian distribution for Simpson's and *d*′ indices with functions *glmer()* or *lmer()*, respectively from the lme4 package (v.1.1‐23; Bates et al., 2014). Explanatory variables were scaled using function *scale()* which computes the mean (centre) of a variable, sets the mean to zero and calculates the values from such a mean. The values are then standardized by the standard deviation of each variable. The response variables were transformed to accomplish comparisons and linear mixed model assumptions (ln for Simpson's index and square root for *d*′‐index). We calculated the variance inflation factor (VIF) of each model to verify the absence of multicollinearity (VIF < 4) with the function *vif()* from package psych (v.2.1.3; Revelle, 2021). We tested whether there was spatial autocorrelation using Moran's index with function *testSpatialAutocorrelation()* from package DHARMa (v.0.4.5; Hartig, 2022).

To analyse the significance of the fixed effects, we performed an ANOVA type III from package car (v.3.0‐9; Fox & Weisberg, 2019) and a post hoc Tuckey test with function *glht()* from package multcomp (v. 1.4‐18; Hothorn et al., 2008). Marginal *R* ^2^ ($R_{m}^{2}$) and conditional *R* ^2^ ($R_{c}^{2}$) were calculated with the *r.squaredGLMM()* function from package MuMIn (v.1.43.17; Bartoń, 2020) to obtain the variance explained by the fixed effects ($R_{m}^{2}$), and by the entire model (with both fixed and random effects, $R_{c}^{2}$), respectively. The residuals met all assumptions of the linear mixed models.

### 2.6 Distributional ranges and specialization patterns

We analysed the distributional range of each mycobiont species and cyanobiont phylogroup considering the distance along the studied gradient in which they were present. Then, we selected those mycobiont species with at least 10 thalli along the entire latitudinal gradient, and we performed linear models with function *lm()* from package stats (R Core Team, 2021) to observe whether generalist mycobiont species associating with many cyanobionts had broader distributional ranges as a lichen. We then determined whether lichen distributional ranges were correlated by the distribution of their *Nostoc* partners.

### 2.7 Spatial scale: Beta diversity and partner replacement

In order to analyse the specialization patterns within mycobiont species among forests and between different spatial scales (local vs. regional), we calculated cyanobiont turnover (Carvalho et al., 2013; Ventre Lespiaucq et al., 2021). We used function *beta()* from package BAT (v.2.7.1; Cardoso et al., 2015) to obtain *β*_total_, *β*_repl_ and *β*_rich_ to determine whether the differences in beta diversity within mycobiont species were due to species replacement and/or due to differences in partner richness (*β*_total_ = *β*_repl_ + *β*_rich_). Specifically, several turnover scenarios have been considered (Ventre Lespiaucq et al., 2021): (a) No turnover: the mycobiont interacts with the same cyanobiots throughout the gradient, (b) Nested loss/gain: the mycobiont maintains many of the same cyanobionts along the gradient, but either gains or loses phylogroups, (c) Partial replacement: the mycobiont loses some cyanobiont phylogroups and gains others. This process could produce the same, lower or higher cyanobiont richness among sites and (d) Total replacement: a shift in cyanobiont composition from site to site. Richness can remain the same or differ among sites.

All statistical analyses were performed in R v. 4.0.4 (http://www.rproject.org/).

## 3 RESULTS

### 3.1 *Nostoc* phylogroup delimitation

We defined a total of 64 *Nostoc* phylogroups based on the results of the supported clades obtained in the combined phylogenetic analyses (i.e. ML and Bayesian analysis which were congruent), together with the grouping generated by ASAP and previous studies (i.e. Magain et al., 2017). Only two phylogroups were shared with phylogroups found by Magain's et al. (2017) (Appendix S3). Phylogroups were named from one to 64, where phylogroups XV and VIIIb from Magain et al. (2017) corresponded to phylogroups one and two, respectively (Appendix S3).

### 3.2 Availability and composition of *Nostoc* phylogroups along the latitudinal gradient

**FIGURE 2:**
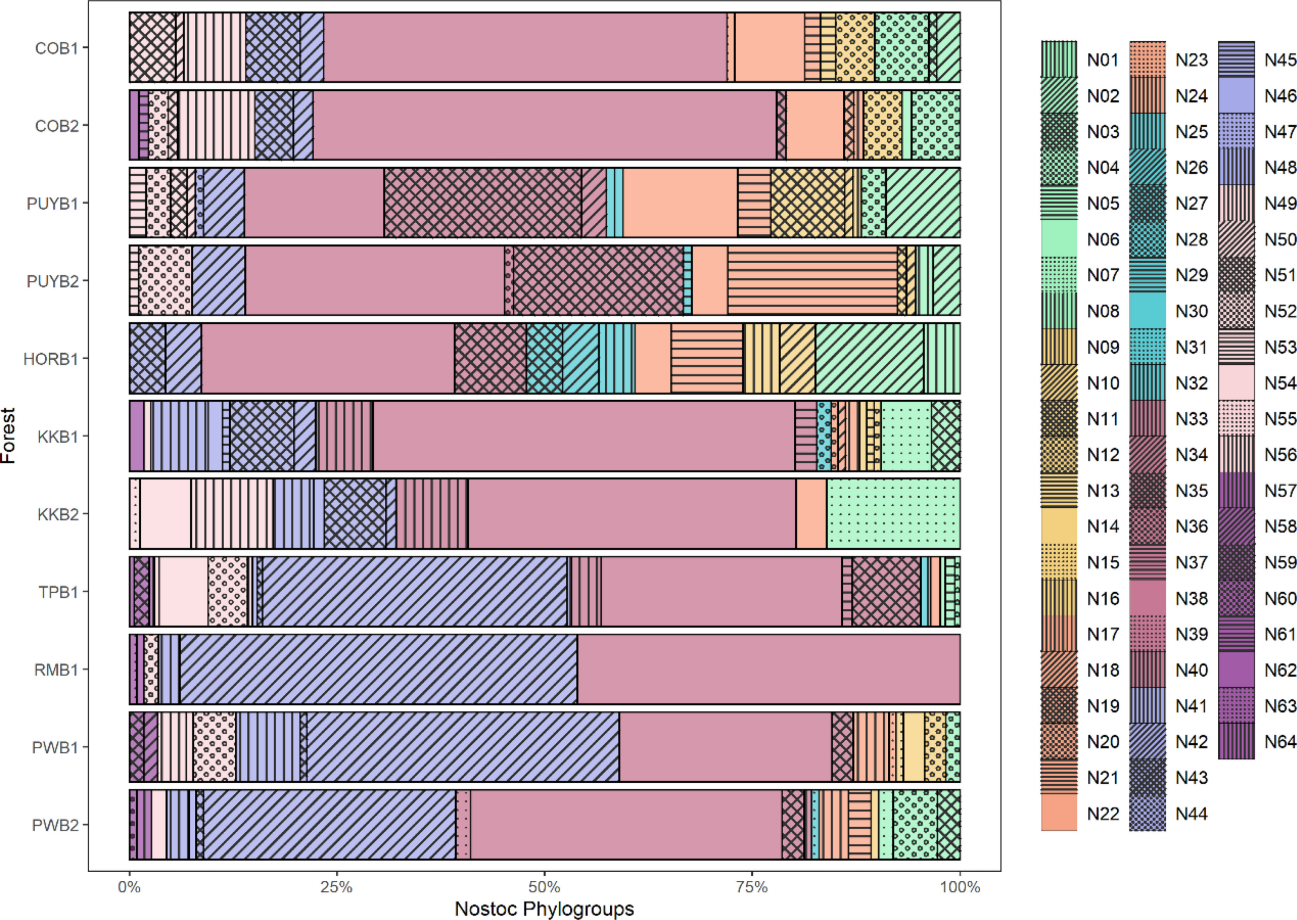
*Nostoc* phylogroups composition on the different forests. Sampling sites: (1) Conguillío National Park (COB1 and COB2), (2) Puyehue National Park (PUYB1 and PUYB2), (3) Hornopirén National Park (HORB1), (4) Cerro Castillo National Park (KKB1 and KKB2), (5) Torres del Paine National Park (TPB1), (6) Magallanes National Reserve (RMB1) and (7) Navarino Island (PWB1 and PWB2).

*Nostoc* phylogroup richness did not show a latitudinal pattern (Appendix S4). However, *Nostoc* phylogroup composition changed among forests (Figure 2). Each forest stand was dominated by a few abundant *Nostoc* phylogroups, but most phylogroups were locally rare. Across the latitudinal gradient studied, *Nostoc* phylogroup 38 was the most abundant, representing 37.32% of the sequences obtained, followed by phylogroup 42, which represented 19.29% (Appendix S5). These abundant phylogroups were shared among different and distantly related mycobiont species.

Mycobiont species differed in their patterns of cyanobiont composition within a forest. Some mycobionts tended to establish with the most abundant *Nostoc* phylogroups while others interacted with rare phylogroups (Appendix S6). Species such as *Nephroma cellulosum*, *Parmeliella nigrocinta*, *Pseudocyphellaria bartlettii*, *P. dubia*, *P. gilva*, *P*. gr. *argyracea*, *P*. gr. *citrina*, *P. lechlerii*, *P. mallota*, *P. scabrosa*, only associated with the same three *Nostoc* phylogroups (35, 38 and 42), which were the most common phylogroups in the gradient. On the contrary, species such as *Leptogium valdivianum*, *Peltigera collina*, *P. hymenina*, *Sticta fuliginosa* and *S. hypochra* were never found associating with the most abundant phylogroups. The rest of the species established, at least once, with the most common phylogroups 35, 38, and/or 42.

Cyanobiont phylogroup composition along the gradient was correlated with climate (mean annual temperature—bio01, minimum temperature of the coldest month—bio06 and precipitation of the driest month—bio14) and forest structure (tree diameter at breast height—DBH) as the RDA analyses showed (Appendix S7). The variation explained by these environmental variables was ca. 70%, with bio06 having the biggest contribution (39.2%), followed by bio01 (30.7%), DBH (20.6%) and bio14 (0.91%) (Appendix S8).

### 3.3 Specialization measures

#### 3.3.1 Number of interacting partners and sampling efficiency

Accumulation curves are shown in Appendix S9 for each mycobiont species and Appendix S10 for each forest. The estimated Chao1 revealed different levels of sampling efficiency, with 23 species (62.16%) showing a Chao1 equal to the number of phylogroups found (sampling of 100% of the phylogroups; Appendix S11). The sampling efficiencies of seven of 37 species were lower than 80%. All the species (even those with a lower sampling efficiency) have been included in subsequent analyses (Simpson's and *d*′‐index calculations, GLMMs and beta diversity), as they already associated with a high number of partners, showing a generalist pattern.

The observed cyanobiont richness within each lichen species along the gradient varied from one to 15 (Appendix S11). *Nephroma antarcticum* was the most generalist lichen species. It interacted with 15 *Nostoc* phylogroups and its Chao1 of 30 suggests that 50% of the expected richness was detected. On the contrary, *Peltigera collina, Pseudocyphellaria bartletti* and *Sticta fuliginosa* were found to be associated only with one *Nostoc* phylogroup, with an estimated sampling efficiency of 100% for each of them (Chao1 = 1).

Considering each forest, the number of cyanobionts observed did not show big differences from the Chao1 estimate, which indicates that the sample size was adequate for estimating within‐forest *Nostoc* richness (*t*(18) = −2.08; *p* > .05) (Appendix S12).

#### 3.3.2 Indices: Simpson and *d*′

**FIGURE 3:**
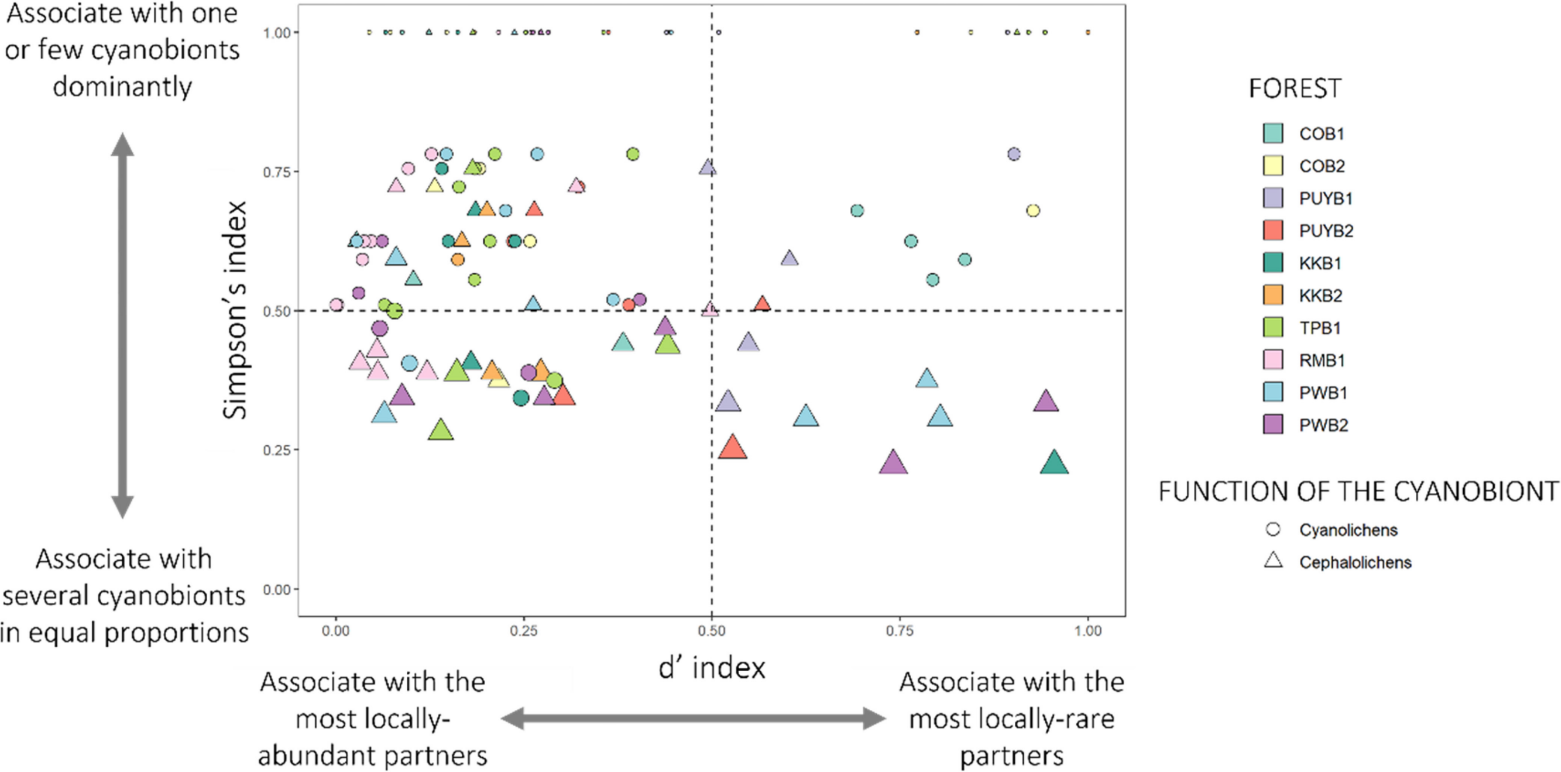
Representation of the values of the three specialization metrics obtained for each lichen species at the forest level (local scale). Circles represent cyanolichens and triangles cephalolichens. The size of circles and triangles refers to the cyanobiont richness of each lichen species, ranging from 1 to 5. The y‐axis represents the values obtained by the Simpson's index and the *x*‐axis the values of the *d*′ index. Colours refer to the studied forests.

Simpson's index and *d*′‐index varied from 0.22 to 1 and 0 to 1, respectively. In addition, most mycobiont species showed different values of each of the indices per forest indicating that the specialization pattern that a species shows in one forest may change across forests (Figure 3; Appendix S13).

Only two species, *Nephroma pseudoparile* and *Peltigera collina*, had a value equal to one in both indices in almost all forests (Figure 3; Appendix S13). Nonetheless, in certain forests, several species (e.g. *Pannaria gr. sphinctrina* (TPB1), *Peltigera collina* (TPB1), *Peltigera hymenina* (PUYB1), *Sticta fuliginosa* (TPB1) and *Sticta hypochra* (COB2)), showed high values of both indices (S‐index = 1; *d*′‐index between 0.94 and 0.84). High values of Simpson's index and low values of *d*′ index were observed in *Nephroma cellulosum* (KKB1), *Parmeliella nigrocinta* (COB2), *Pseudocyphellaria gr. citrina* (COB1) and *Pseudocyphellaria hirsuta* (COB2). The opposite pattern (i.e. a low S‐index and a high *d*′‐index) was found in fewer species, such as *Pseudocyphellaria freycinettii* (PWB2) and *Psoroma asperellum* (KKB1). In addition, examples of varying patterns along the latitudinal gradient were observed in *Pseudocyphellaria* gr. *vaccina* and *Nephroma pseudoparile*. For instance, *Nephroma pseudoparile* showed high values of both indices in the Northernmost forests, above the Patagonian ice fields, but low values (S‐index = 0.38; *d*′‐index = 0.29) in its Southern distribution, thus being more specialized towards the North.

#### 3.3.3 Generalized linear mixed models

**FIGURE 4:**
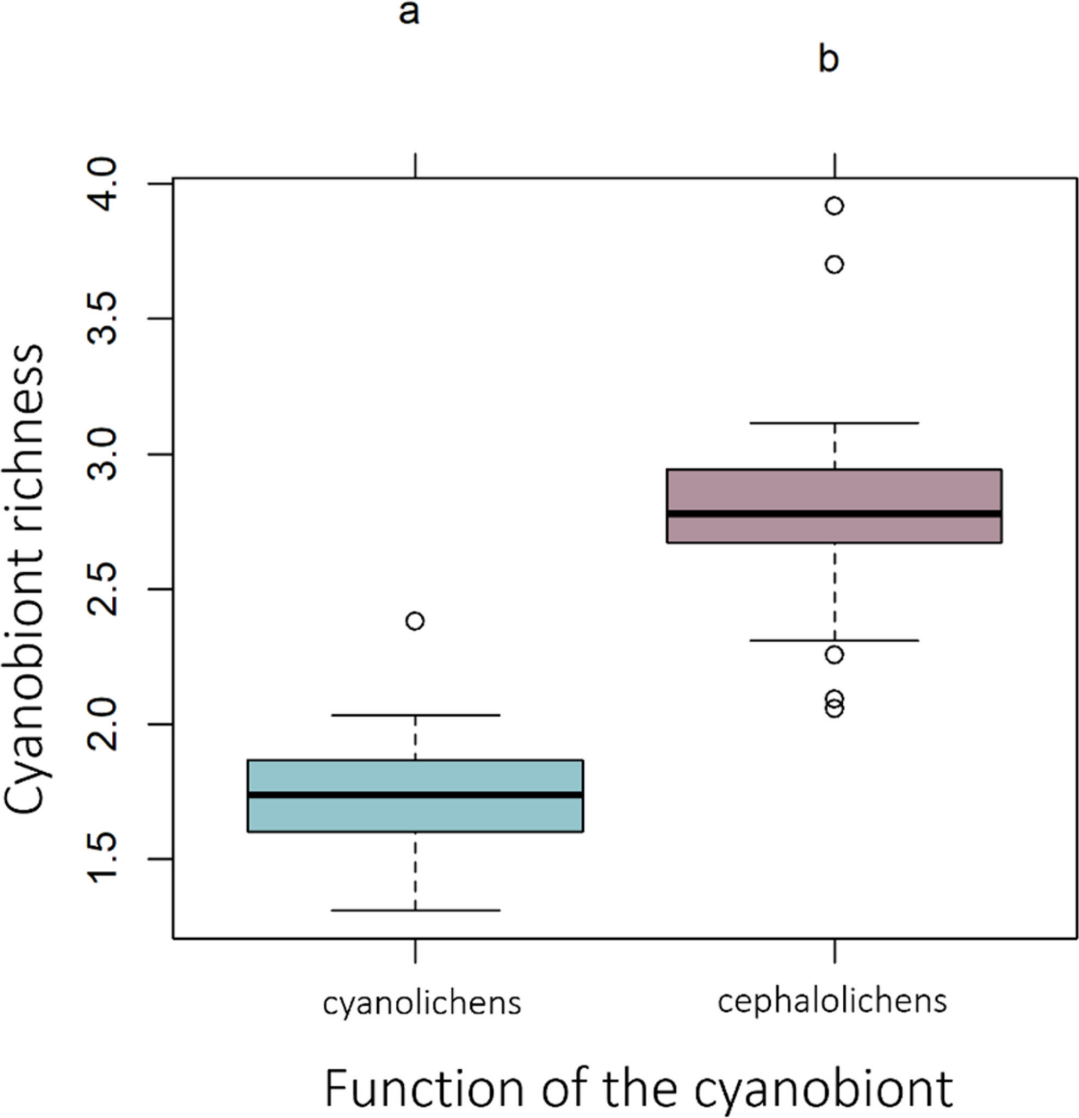
Cyanobiont richness for cyano‐ and cephalolichens. Letters inform about the significant differences after performing a Tuckey test.

**FIGURE 5:**
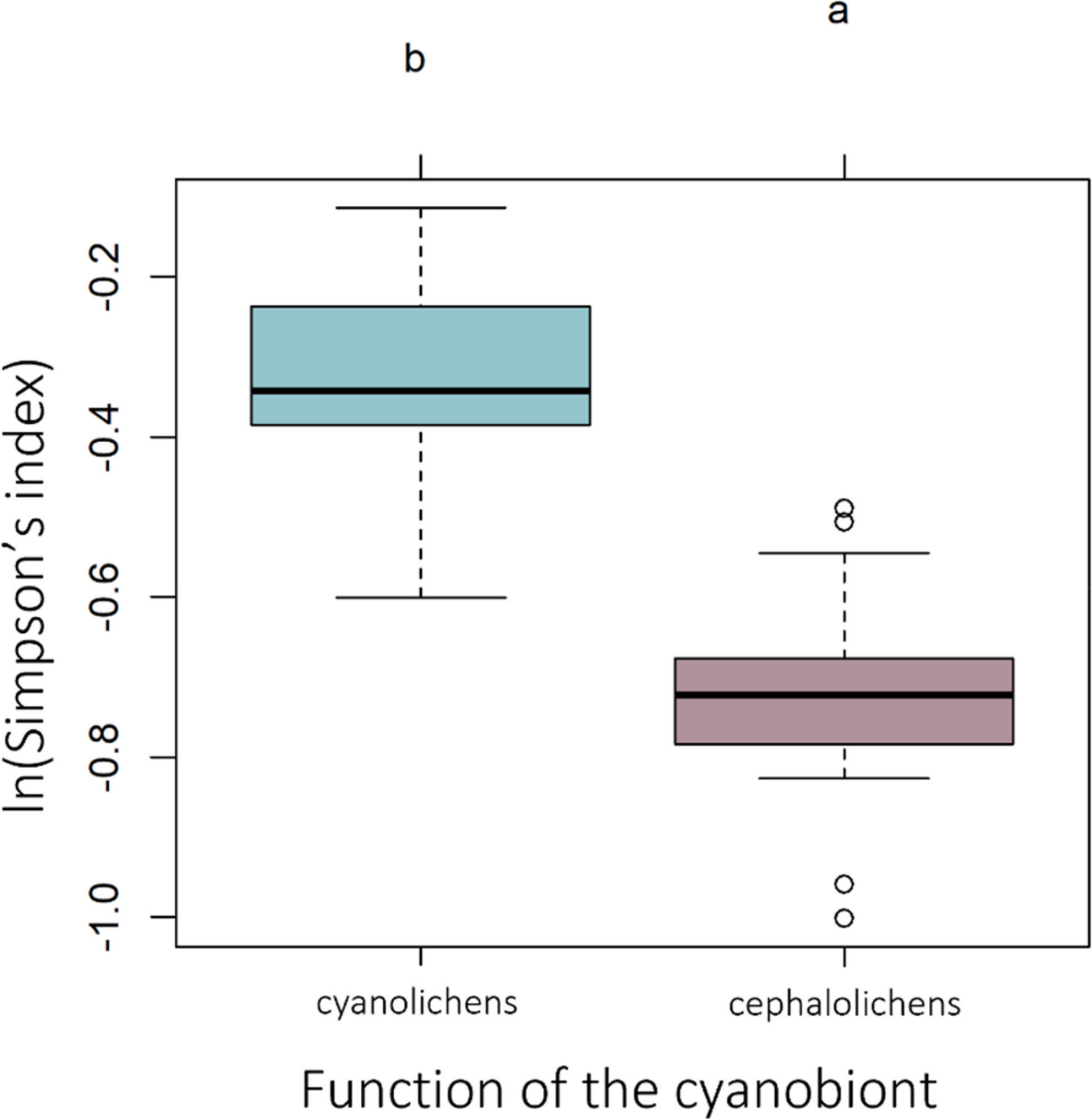
Simpson's index for cyano‐ and cephalolichens. Letters inform about the significant differences after performing a Tuckey test.

The results of the GLMMs for the three metrics are presented in Table 1. Among the fixed effects in the model, the function of the cyanobiont was the factor explaining the highest proportion of variation for cyanobiont richness (*X* ^2^ (1, *N* = 120) = 12.10, *p* > .05) and Simpson's index (*X* ^2^ (1, *N* = 120) = 30.53, *p* > .05). Cephalolichens tend to interact with a higher number of cyanobionts than cyanolichens (Figure 4). In addition, cephalolichens show lower values of Simpson's index than cyanolichens, which means they interact with the different partners in more equal proportions, not showing preferential interactions with any of them (Figure 5). Random effects (mycobiont species identity and forest) explained little to no variance in cyanobiont richness and Simpson's index (cyanobiont richness: $R_{m}^{2}$ = $R_{c}^{2}$ = 0.13; S‐index: $R_{m}^{2}$ = $R_{c}^{2}$ = 0.28). On the contrary, the variation of the *d*′‐index was explained mostly by the random effects ($R_{m}^{2}$ = 0.13; $R_{c}^{2}$ = 0.75), being the largest part of the variance explained by the mycobiont species identity. The environmental variables (bio01, bio06, bio14 and DBH) and the reproductive mode (sexual vs. asexual) had little effect in all the specialization measures.

**TABLE 1:** Results of the GLMMs. Bold values refer to a significant effect of the fixed effects.

Random effects	Partner richness		Simpson index		*d*′ index	
	Variance	SD	Variance	SD	Variance	SD
Mycobiont species	0	0	0	0	0.04	0.2
Forest	0	0	0	0	0	0.05
Residual			0.12	0.35	0.02	0.13

Fixed effects	Estimate	SD	*z*	Chisq	df	*p*	Estimate	SD	*t*	Chisq	df	*p*	Estimate	SD	*t*	Chisq	df	*p*
Intercept	0.49	0.11	4.43	19.64	1	9.38e‐06 ***	−0.29	0.05	−5.51	30.38	1	3.58e‐08 ***	0.61	0.05	11.2	125.38	1	<2e‐16 ***
Scale (DBH)	0.01	0.12	0.49	0	1	.96	−0.01	0.06	−0.12	0.01	1	.91	−0.04	0.04	−1.22	1.48	1	.22
Scale (bio01)	−0.12	0.12	−1.03	1.05	1	.31	0.1	0.06	1.82	3.31	1	.07	0.05	0.04	1.54	2.36	1	.12
Scale (bio06)	0.07	0.1	0.67	0.45	1	.5	−0.05	0.05	−1.12	1.269	1	.26	−0.02	0.03	−0.66	0.44	1	.51
Scale (bio14)	0.07	0.11	0.68	0.46	1	.5	−0.05	0.05	−0.89	0.8	1	.37	0.03	0.03	1.04	1.08	1	.3
Reproduction				1.02	3	.8				1.8	3	.61				3.39	3	.34
Reproduction: both	0.1	0.18	0.55				−0.04	0.09	−0.42				0.02	0.05	0.41			
Reproduction: none	0.3	0.34	0.89				−0.24	0.21	−1.13				0.25	0.14	1.84			
Reproduction: sexual	0.07	0.14	0.51				−0.07	0.07	−0.89				0.03	0.06	0.44			
Cyanobiont				12.1	1	5.04e‐04 ***				30.53	1	3.28e‐08 ***				2.1	1	.15
Cyanobiont: cephalolichen	0.45	0.13	3.48				−0.38	0.07	−5.53				−0.11	0.08	−1.45			

*Note*: Random and fixed effects estimates and type III ANOVA for partner richness, Simpson index and *d*′ index of specialization.

### 3.4 Distributional ranges and specialization patterns

The relationship between the number of partners of each mycobiont species and the geographic distance did not appear to be linear (Appendix S14). Those lichen species with broad distributional ranges are either associated with a high number of interacting partners or (most frequently) associated with a low number of interacting partners with a broad distributional range (e.g. *Pseudocyphellaria citrina*). Most mycobiont species established at least once, with widely distributed *Nostoc* phylogroups; thus, cyanobionts were not limiting lichen distributions (Appendices S15 and S16). On the contrary, examples of limitations in the distributional range of a lichen species due to the distributional range of the *Nostoc* partner are rare (besides the cases in which there are few observations). For instance, *Nostoc* phylogroup 02 has a small distributional range (361.24 km). This phylogroup only associates with *Peltigera praetextata* and *Peltigera hymenina*. *Peltigera praetextata* (with five thalli) only associates with phylogroup 02 and shares its distributional range. On the contrary, *Peltigera hymenina*, also sharing the same distributional range, associates likewise with phylogroup 43, which has a distributional range of 1841.91 km. Thus, *Peltigera hymenina* distributional range is not limited by its cyanobionts (Appendices S15 and S16).

Only one case with enough number of samples was found in which both partners matched their distributional ranges. Moreover, this case showed reciprocal specialization (*Peltigera collina* with *Nostoc* phylogroup 40).

### 3.5 Spatial scale: Beta diversity and partner replacement

We found that most mycobiont species increased their number of partners when increasing the spatial scale following; mostly, a nested turnover scenario when comparing each forest with the regional gradient (Appendix S17). This increase in generalization in larger spatial scales is due to differences in the frequency and the identity of the interactions between different forests (Appendices S6 and S18). Only four species (*Peltigera collina*, *Pseudocyphellaria bartlettii*, *Pseudocyphellaria mallota* and *Sticta fuliginosa*) were found to show an exclusive pattern of no turnover and, thus, maintaining their specialization pattern across spatial scales. The resting 33 species toggled between no turnover (15 observations) to nested turnover (98 observations) between each forest and the regional scale.

## 4 DISCUSSION

Our study highlights the varying levels of specialization in epiphytic lichen communities along a wide latitudinal gradient in Chile. Environmental factors may influence specialization by determining the composition and availability of *Nostoc* communities in the studied forests, therefore conditioning the potential interactions. However, a decrease in *Nostoc* phylogroup richness was not seen towards the south, suggesting that specialization does not seem to be influenced by a lower availability of potential partners in the Southernmost part of the gradient. Moreover, we did not find a relation between the distributional range of lichen species and the distributional range of their cyanobionts. Specialization depends on the analysed index and the spatial scale considered. The functional contribution of the cyanobiont to the lichen association influenced specialization measured as cyanobiont richness and Simpson's index, but with the *d*′‐index, the mycobiont identity was the factor explaining most of the variance. On the contrary, the specification of the spatial scale is essential as specialization patterns changed in most species when considering each forest separately (local scale) or the whole latitudinal gradient (regional scale), showing a tendency towards generalism when the spatial scale increased. Differences in the number and identity of cyanobionts associated with lichen species can be assessed by different turnover scenarios, in which *Nostoc* phylogroups are added or replaced between forests. Therefore, considering the relevance of environmental variables influencing *Nostoc* pool composition, mycobionts are expected to establish with locally adapted cyanobionts.

In contrast with what we expected from the latitudinal diversity gradient hypothesis (Hillebrand, 2004), we did not find a latitudinal diversity pattern for *Nostoc* phylogroup richness along the gradient studied. However, in agreement with other studies, environmental drivers influenced *Nostoc* composition on the different forests (Fernández‐Mendoza et al., 2011; Muggia et al., 2013, 2014; Nadyeina et al., 2014; Vargas Castillo & Beck, 2012). Our results showed that temperature‐related variables (bio06 and bio01) explained most of the observed composition of *Nostoc* phylogroups along the gradient, followed by the DBH, which informs about the forest structure. These results suggest that differences in cyanobiont pools found along the gradient could be due to environmental filtering leading to the prevalence of better locally adapted phylogroups to these environmental conditions (Batstone et al., 2018; Nelsen et al., 2021; Rolshausen et al., 2020). In accordance with previous studies, this fact (i.e. establishing an interaction with locally adapted phylogroups) may benefit the fitness of the association (Batstone et al., 2018; Magain et al., 2017; Thompson, 2005). However, these assumptions should be tested experimentally through physiological or demographical approaches (e.g. photosynthesis performance or growth rate under different environmental conditions of the same mycobiont associating with different partners, among others). Nonetheless, the factors determining the composition of the *Nostoc* pool may be different in other habitats, such as soil or rocks, as in the current study we focused on epiphytic lichen communities growing on *Nothofagus pumilio*.

Mycobiont species showed variable values of the three metrics used to quantify specialization (cyanobiont richness, Simpson's and *d*′ index) depending on the forest considered. These changes in the specialization metrics across different forests inform about a dynamic pattern of specialization, in which species can change from a specialized to a more generalized pattern in different localities, probably related to the local adaptation of the partners. As a result, the specialization pattern will differ when considering different spatial scales (local vs. regional). Nonetheless, the factors influencing specialization measures were different depending on the specialization index considered. While the function of the cyanobiont partly explained cyanobiont richness and Simpson's index, mycobiont species identity mostly affected the *d*′‐index. Thus, cephalolichens showed a less specialized pattern (higher number of interacting cyanobionts and lower values of Simpson's index) than cyanolichens. Little is known about specialization in cyanolichens and cephalolichens, and in contrast to our results, some studies have found either a high specialization in cephalolichens (Paulsrud et al., 2001) or no differences between both (Pardo‐De la Hoz et al., 2018; Wirtz et al., 2003). Differences with previous studies may be due to the different sampling designs, as no other study has considered the whole lichen community as we do here or may be due to the lower number of cephalolichens species found in other regions (i.e. Europe). The lower specialization found in cephalolichens may be related to the relative importance of the cyanobiont to fulfil the requirements in the lichen symbiosis in bi‐ and tripartite lichens (Palmqvist, 2002; Rai, 2002). In cyanolichens, the cyanobionts have the photosynthetic function, and the interaction is obliged to result in the lichen symbiosis; meanwhile, in cephalolichens, the main photosynthetic partner is a green alga and the cyanobiont's main function is nitrogen fixation (Paulsrud et al., 2001; Rai et al., 2000). This is translated in a smaller dependency for the cyanobiont in cephalolichens, explaining their lower specialization. On the contrary, the *d*′‐index is influenced by mycobiont species identity, being the main factor conditioning the interactions. Previous studies (Dal Grande et al., 2018; Fedrowitz et al., 2011; Jüriado et al., 2019; Leavitt et al., 2015) also found that the mycobiont identity determined specialization with little influence of the environment or other factors. Thus, considering the whole *Nostoc* pool, the association with more abundant (opportunism) or rare phylogroups is determined by the fungus identity, which may be genetically constrained. Nonetheless, we observed a tendency in which those cephalolichens establishing with a larger number of partners, interacted with rather rare cyanobionts, showing higher values of the *d*′‐index. Thus, increasing the flexibility to associate with higher number of partners could allow interacting with low abundant partners in the community. Environmental drivers did not explain any of the specialization metrics directly, but as previously described, may influence specialization through their effect in the composition and potential availability of the *Nostoc* community within a forest. This may be related to the small variation in the temperature‐related variables along the gradient.

Surprisingly and contrary to previous studies (Cao et al., 2015; Dal Grande et al., 2014, 2018; Fedrowitz et al., 2011, 2012; Otálora et al., 2010), lichens' reproductive mode did not drive specialization in the studied communities. Cyanobint switching or turnover in asexually reproducing lichens could be the explanation of the lack of relationship between the reproductive mode and specialization. This cyanobiont switching would lead to a shift from a vertical transmission of the cyanobiont (in which both symbionts are dispersed together) to a horizontal transmission by replacement of the previous cyanobiont (Ertz et al., 2018; Ohmura et al., 2019; Piercey‐Normore & DePriest, 2001; Rolshausen et al., 2018; Vidal‐Russell & Messuti, 2017). As a result, in both reproductive modes (sexual and asexual), the cyanobiont can be newly acquired (horizontally transmitted), explaining the absence of a differential specialization pattern between sexual and asexually reproducing lichens. These findings are in agreement with the observed changing pools of *Nostoc* cyanobionts along the gradient as mycobionts can switch to a locally adapted cyanobiont. This might contribute to different geographic mosaics of symbiotic interactions (Fedrowitz et al., 2012; Magain et al., 2017; Thompson, 2005).

When considering the distribution of the lichen, we found that lichens with wide distributional ranges, could either belong to mycobiont species that associate with a high number of *Nostoc* phylogroups in the entire gradient by replacing their partners along their distributional range, or with a low number of widely distributed *Nostoc* phylogroups, as phylogroup 38 (Magain et al., 2017; Rolshausen et al., 2020; Ventre Lespiaucq et al., 2021). Thus, cyanobiont switches to locally adapted partners could be a mechanism to increase the geographic and ecological niche of a given lichen species (Fernández‐Mendoza et al., 2011; Muggia et al., 2014; Peksa & Škaloud, 2011). However, partner availability is not necessarily the unique limiting factor for lichens' distributions and other variables should be taken into account, as several species have narrow distributional ranges, but associate with wide‐distributed cyanobionts (e.g. *Pseudocyphellaria mallota*; Lu et al., 2018).

Our results highlight the importance of defining the spatial scale in the study of specialization patterns in symbiotic organisms, as other studies have previously shown, in order to understand the relationship between cyanobiont pools and environmental filtering (Rolshausen et al., 2018, 2020). As expected, the studied lichen species showed a tendency to increase the number of interacting partners, and a decline of specialization when the spatial scale was increased, instead of maintaining the same number of partners along their distribution range. The decrease in specialization at larger scales is in agreement with studies proposing that partner replacement could occur in order to establish with better adapted partners (e.g. Fernández‐Mendoza et al., 2011). A transplantation experiment could be useful to test this turnover of partners under different environments (see Williams et al., 2017).

Not considering the complete community may limit the consideration of specialization. Most studies are focused on low biological ranks, thus missing the photobiont pool of the local community (e.g. Dal Grande et al., 2018; Leavitt et al., 2015; Magain et al., 2017; Pino‐Bodas & Stenroos, 2021; Werth & Sork, 2010). The consideration of the total community allows to determine the pool of photobionts and their proportions in the community, which are paramount to consider specialization (Blüthgen et al., 2006; Vázquez et al., 2007). With this information, specialization can be measured not only in terms of the number and frequency of associations (partner richness and Simpson's index, respectively), but also considering the opportunism of those interactions (*d*′ index). The combination of the three metrics offers an integrative approach of the consideration of specialization within its continuum nature. The community context, together with the wide latitudinal gradient studied, provided results that could be extrapolated to other lichen communities from other habitat types, helping and improving our knowledge about specialization in this intimate symbiotic system.

In summary, our study shows how specialization varies within the axis of partner availability in the hypervolume of the niche in lichenized symbiotic associations. Mycobiont species showed a variable number of cyanobionts and different values of Simpson and *d*′ indices. Therefore, the metric used to quantify specialization influences the understanding of specialization in mutualistic interactions. Cyanolichens were more specialized when considering the cyanobiont richness and Simpson index than cephalolichens, probably due to the relative obliged interaction with the cyanobiont to stablish the symbiosis. However, when considering the whole pool of *Nostoc* symbionts when measuring the *d*′‐index for each mycobiont species, is the mycobiont identity what mostly affects the specialization pattern acquired. As well, we found that specialization can be considered differently depending on the spatial scale, with a general pattern of changes in the constancy of associations by adding or replacing cyanobionts between different forests. Because of the relevance of environmental factors shaping the composition of *Nostoc* cyanobiont communities, mycobionts may interact with different cyanobionts across their distributional range, which could enhance the chances of finding better locally adapted phylogroups. Thus, mycobionts distributional ranges are barely affected by cyanobiont availability. As a result, we found a tendency to increase partner richness when increasing the spatial scale, with a dominant nested turnover scenario produced by the addition or loss of some cyanobionts and the maintenance of certain cyanobionts among forests. As a whole, our results encourage to consider the whole community and the relative abundance of available partners, as well as the proportion of interactions with each partner to be able to quantify specialization more informatively with complementary metrics. Nonetheless, we also promote the importance of considering specialization as a scale‐dependent concept.

## AUTHOR CONTRIBUTIONS

**Clara Rodríguez‐Arribas:** Conceptualization (equal); data curation (lead); formal analysis (lead); investigation (equal); methodology (equal); writing – original draft (lead); writing – review and editing (equal). **Isabel Martínez:** Conceptualization (equal); funding acquisition (lead); investigation (equal); methodology (equal); project administration (lead); resources (equal); supervision (lead); validation (equal); writing – original draft (equal); writing – review and editing (equal). **Gregorio Aragón:** Conceptualization (equal); data curation (equal); funding acquisition (lead); project administration (lead); supervision (equal). **Carlos Zamorano‐Elgueta:** Conceptualization (equal); funding acquisition (equal); investigation (equal); resources (equal); validation (equal). **Lohengrin Cavieres:** Conceptualization (equal); funding acquisition (equal); project administration (equal); resources (equal). **María Prieto:** Conceptualization (equal); data curation (equal); funding acquisition (lead); investigation (equal); methodology (equal); project administration (lead); supervision (lead); validation (equal); writing – original draft (equal); writing – review and editing (equal).

## Supporting information


Appendix S1

## Data Availability

All the data from the current study are contained in the Supporting Information or have been uploaded into the Zenodo repository as: Rodríguez‐Arribas et al. (2023). Data from: Specialization patterns in symbiotic associations: a community perspective over spatial scales. [Data set]. Zenodo. https://doi.org/10.5281/zenodo.7674732.
