# Supplementary material for: Specialization patterns in symbiotic associations: A community perspective over spatial scales
Source: Ecol Evol. 2023 Jul 10;13(7):e10296. doi: 10.1002/ece3.10296 (PMC10333671; doi:10.1002/ece3.10296)
Supplement: Supplementary file 1 — Appendix S1 [file ECE3-13-e10296-s001.docx]

**Supplemental Information for:**

**Specialization patterns in symbiotic associations: a community perspective over spatial scales.**

Rodríguez-Arribas, C., Martínez, I., Aragón, G., Zamorano-Elgueta, C., Cavieres, L. & Prieto, M.

**Table of Contents:**

| **Appendix S1** | Page 1 |
| --- | --- |
| **Appendix S2** | Page 2-3 |
| **Appendix S3** | Page 4 |
| **Appendix S4** | Page 5 |
| **Appendix S5** | Page 6-7 |
| **Appendix S6** | Page 8-12 |
| **Appendix S7** | Page 13 |
| **Appendix S8** | Page 13 |
| **Appendix S9** | Page 14-15 |
| **Appendix S10** | Page 16 |
| **Appendix S11** | Page 17 |
| **Appendix S12** | Page 18 |
| **Appendix S13** | Page 19-21 |
| **Appendix S14** | Page 22 |
| **Appendix S15** | Page 23-24 |
| **Appendix S16** | Page 25-27 |
| **Appendix S17** | Page 28-31 |
| **Appendix S18** | Page 32-37 |

Appendix S1 Environmental variables: 1) Geographic variables: Latitude and Longitude (º), 2) Forest structure and habitat quality related variables: Altitude (mlsa), Orientation (º), Inclination (º), DBH (m) and canopy cover (%), 3)Climate related variables extracted from CHELSA climate database (Karger et al. 2017): Annual Mean Temperature (bio1), Mean Diurnal Range (BIO2), Isothermality (BIO3), Temperature Seasonality (BIO4), Max Temperature of Warmest Month (BIO5), Min Temperature of Coldest Month (BIO6), Temperature Annual Range (BIO7), Mean Temperature of Wettest Quarter (BIO8), Mean Temperature of Driest Quarter (BIO9), Mean Temperature of Warmest Quarter (bio10), Mean Temperature of Coldest Quarter (bio11), Annual Precipitation (bio12), Precipitation of Wettest Month (bio13), Precipitation of Driest Month (bio14), Precipitation Seasonality (bio15), Precipitation of Wettest Quarter (bio16), Precipitation of Driest Quarter (bio17), Precipitation of Warmest Quarter (bio18), and Precipitation of Coldest Quarter (bio19). Temperature in ºC, precipitation in mm.

Sampling sites: 1) Conguillío National Park (COB1 and COB2), 2) Puyehue National Park (PUYB1 and PUYB2), 3) Hornopirén National Park (HORB1), 4) Cerro Castillo National Park (KKB1 and KKB2), 5) Torres del Paine National Park (TPB1), 6) Magallanes National Reserve (RMB1) and 7) Navarino Island (PWB1 and PWB2).

|  | GEOGRAPHIC  VARIABLES | | FOREST STRUCTURE AND  HABITAT QUALITY VARIABLES | | | | | CLIMATE | | | | | | | | | | | | | | | | | | |
| --- | --- | --- | --- | --- | --- | --- | --- | --- | --- | --- | --- | --- | --- | --- | --- | --- | --- | --- | --- | --- | --- | --- | --- | --- | --- | --- |
|  |  |  |  | | | | | TEMPERATURE RELATED VARIABLES | | | | | | | | | | | PRECIPITATION RELATED VARIABLES | | | | | | | |
| BOSQUE | Longitude | Latitude | Altitude | Orientation | Inclination | DBH | Canopy | bio01 | bio02 | bio03 | bio04 | bio05 | bio06 | bio07 | bio08 | bio09 | bio10 | bio11 | bio12 | bio13 | bio14 | bio15 | bio16 | bio17 | bio18 | bio19 |
| COB1 | -71.7 | -38.6 | 1373.2 | 117.2 | 11 | 1.92 | 77.60 | 6.1 | 9.1 | 40 | 438.9 | 19.2 | -3.5 | 22.7 | 1.6 | 12.5 | 12.5 | 0.4 | 1611 | 271 | 36 | 60 | 788 | 114 | 114 | 767 |
| COB2 | -71.6 | -38.6 | 1487.2 | 185.6 | 18.3 | 1.49 | 84.00 | 6 | 9.1 | 39.9 | 443.2 | 19.1 | -3.7 | 22.8 | 1.4 | 12.4 | 12.4 | 0.2 | 1459 | 247 | 34 | 60 | 718 | 107 | 107 | 685 |
| PUYB1 | -72.2 | -40.8 | 978.4 | 75.6 | 9 | 1.21 | 69.50 | 7 | 7.2 | 37.9 | 380.4 | 17.8 | -1.2 | 19.1 | 3.1 | 12.5 | 12.5 | 2 | 2370 | 323 | 96 | 43 | 965 | 292 | 292 | 931 |
| PUYB2 | -72.2 | -40.8 | 1237.6 | 288 | 28.7 | 1.25 | 73.40 | 5.9 | 7.2 | 38 | 381 | 16.8 | -2.3 | 19.1 | 2 | 11.4 | 11.4 | 0.9 | 2388 | 320 | 103 | 41 | 958 | 312 | 312 | 923 |
| HORB1 | -72.34 | -41.84 | 913 | 64.8 | 12.8 | 1.15 | 71.72 | 6.7 | 6.1 | 36.7 | 336.4 | 16 | -0.5 | 16.5 | 3.4 | 10.2 | 11.6 | 2.3 | 1680 | 226 | 86 | 35 | 664 | 270 | 288 | 638 |
| KKB1 | -72.0 | -46.1 | 1092.6 | 196.4 | 16 | 1.41 | 86.00 | 2.9 | 5.8 | 34.5 | 353.9 | 11.9 | -5 | 16.9 | -2.1 | 7.8 | 7.8 | -2.1 | 778 | 117 | 31 | 47 | 321 | 100 | 100 | 321 |
| KKB2 | -72.2 | -46.1 | 857.2 | 119.2 | 17.1 | 1.43 | 85.60 | 5.1 | 5.8 | 34.5 | 349.3 | 14 | -2.8 | 16.8 | 0.2 | 10 | 10 | 0.2 | 722 | 104 | 32 | 42 | 282 | 103 | 103 | 282 |
| TPB1 | -73.2 | -51.1 | 340.4 | 142.6 | 14.8 | 1.45 | 84.80 | 5.1 | 4.7 | 33.9 | 315.6 | 12 | -1.8 | 13.8 | 3.8 | 6.4 | 9.2 | 0.5 | 853 | 98 | 51 | 20 | 284 | 163 | 164 | 232 |
| RMB1 | -71.0 | -53.1 | 387 | 189.6 | 8.9 | 1.35 | 86.50 | 4.2 | 4.3 | 34 | 297.6 | 10.7 | -2 | 12.7 | 3 | 3.7 | 8.2 | 0 | 444 | 47 | 27 | 17 | 140 | 86 | 112 | 96 |
| PWB1 | -67.7 | -55.0 | 294 | 288.8 | 17.4 | 1.29 | 85.00 | 4.9 | 4.1 | 35.2 | 268.9 | 11 | -0.7 | 11.7 | 8.5 | 5.9 | 8.6 | 1.2 | 528 | 53 | 34 | 14 | 156 | 104 | 139 | 119 |
| PWB2 | -67.6 | -55.0 | 407 | 328.4 | 25.6 | 1.20 | 81.50 | 4.4 | 4.1 | 35.2 | 269.5 | 10.4 | -1.2 | 11.7 | 7.9 | 5.4 | 8 | 0.6 | 648 | 68 | 42 | 15 | 196 | 129 | 178 | 138 |

Appendix S2 Lichen species found along the gradient with the number of cyanobiont sequences per species (N sequences), the number of forest (N forests) in which the species is present and the number of interacting phylogroups (partner richness).

| **Specie** | **N sequences** | **N forests** | **Partner richness** |
| --- | --- | --- | --- |
| *Collema flaccidum* (Ach.) Ach. | 18 | 7 | 7 |
| *Collema glaucophthalmum* Nyl. | 4 | 1 | 3 |
| *Crocodia guilleminii* (Mont.) Nyl. | 26 | 6 | 4 |
| *Cyanisticta obvoluta* (Sw.) C.W. Dodge | 37 | 8 | 6 |
| *Fuscopannaria mediterranea* (Tav.) P.M. Jørg. | 4 | 1 | 1 |
| *Fuscopannaria minor* (Darb.) P.M. Jørg. | 13 | 5 | 3 |
| *Fuscopannaria* sp. *1* | 3 | 2 | 2 |
| *Leciophysma* sp. *1* | 1 | 1 | 1 |
| *Leptogium* aff. *tenuissimum* | 7 | 1 | 6 |
| *Leptogium azureum* (Sw.) Mont. | 3 | 2 | 2 |
| *Leptogium cochleatum* (Dicks.) P.M. Jørg. & P. James | 1 | 1 | 1 |
| *Leptogium decipiens* P.M. Jørg. | 15 | 5 | 5 |
| *Leptogium laceroides* B. de Lesd. | 9 | 3 | 3 |
| *Leptogium menziesii* (Sm.) Mont. | 6 | 3 | 2 |
| *Leptogium patagonicum* Zahlbr. | 5 | 1 | 2 |
| *Leptogium* sp. *1* | 1 | 1 | 1 |
| *Leptogium valdivianum* M. Lindstr. | 12 | 4 | 4 |
| *Nephroma analogicum* Nyl. *(+ N. chubutense* I.M. Lamb*)* | 11 | 3 | 6 |
| *Nephroma antarcticum* (Wulfen) Nyl. | 61 | 11 | 15 |
| *Nephroma cellulosum* (Ach.) Ach. | 49 | 10 | 3 |
| *Nephroma kuehnemannii* I.M. Lamb *(+ N. microphyllum* Henssen*)* | 2 | 2 | 2 |
| *Nephroma parile* (Ach.) Ach. | 28 | 4 | 3 |
| *Nephroma plumbeum* (Mont.) Mont. | 2 | 2 | 2 |
| *Nephroma pseudoparile* (Räsänen) Zahlbr. | 4 | 2 | 1 |
| *Nephroma skottsbergii* F.J. White & P. James *(+ N. papillosum* F.J. White & P. James*)* | 20 | 4 | 7 |
| *Pannaria* aff. *implexa* | 5 | 1 | 2 |
| *Pannaria* aff. *patagonica* | 2 | 1 | 1 |
| *Pannaria arthroophylla* (Stirt.) Elvebakk & D.J.Galloway | 3 | 1 | 2 |
| *Pannaria byssoidea* Passo & Calvelo | 7 | 1 | 2 |
| *Pannaria contorta* (Müll. Arg.) Passo & Calvelo | 1 | 1 | 1 |
| *Pannaria farinose* Elvebakk & Fritt-Rasm | 23 | 5 | 4 |
| *Pannaria* gr. *sphinctrina* | 20 | 5 | 6 |
| *Pannaria pallida* (Nyl.) Hue | 24 | 8 | 3 |
| *Pannaria pulverulacea* Elvebakk | 20 | 7 | 8 |
| *Pannaria* sp. *1* | 2 | 1 | 1 |
| *Parmeliella nigrata* (Müll. Arg.) P.M. Jørg. & D.J. Galloway | 4 | 3 | 2 |
| *Parmeliella nigrocinta* (Mont.) Müll. Arg*.* | 18 | 6 | 3 |
| *Parmeliella* sp. *1* | 1 | 1 | 1 |
| *Phormopsora isabellina* (Vain.) Elvebakk, S.G. Hong & C.H. Park | 1 | 1 | 1 |
| *Peltigera canina* (L.) Willd. | 6 | 2 | 2 |
| *Peltigera collina* (Ach.) Schrad. | 20 | 3 | 1 |
| *Peltigera degenii* Gyeln. | 2 | 1 | 1 |
| *Peltigera hymenina* (Ach.) Delise | 20 | 5 | 2 |
| *Peltigera membranacea* (Ach.) Nyl. | 8 | 3 | 1 |
| *Peltigera polydactylon* (Neck.) Hoffm. | 5 | 3 | 1 |
| *Peltigera praetextata* (Flörke ex Sommerf.) Zopf | 4 | 4 | 1 |
| *Peltigera rufescens* (Weiss) Humb. | 4 | 1 | 1 |
| *Peltigera* sp. *1* | 2 | 1 | 1 |
| *Podostictina berberina* (G. Forst.) B. Moncada & Lücking | 3 | 3 | 1 |
| *Podostictina encoensis* (R. Sant.) D.J. Galloway & de Lange | 1 | 1 | 1 |
| *Podostictina flavicans* (Hook. f. & Taylor) B. Moncada & Lücking | 8 | 3 | 3 |
| *Pseudocyphellaria bartlettii* D.J. Galloway | 10 | 3 | 1 |
| *Pseudocyphellaria coppinsii* D.J. Galloway | 7 | 2 | 3 |
| *Pseudocyphellaria divulsa* (Taylor) Imshaug | 1 | 1 | 1 |
| *Pseudocyphellaria dubia* Du Rietz | 23 | 4 | 2 |
| *Pseudocyphellaria faveolate* (Delise) Malme | 17 | 5 | 3 |
| *Pseudocyphellaria freycinetti* (Delise) Malme | 15 | 3 | 8 |
| *Pseudocyphellaria gilva* (Ach.) Malme | 34 | 6 | 3 |
| *Pseudocyphellaria glabra* (Hook. f. & Taylor) C.W. Dodge | 21 | 5 | 4 |
| *Pseudocyphellaria* gr. *argyracea* | 58 | 10 | 2 |
| *Pseudocyphellaria* gr. *citrina* | 67 | 11 | 3 |
| *Pseudocyphellaria* gr. *vaccina* | 36 | 7 | 13 |
| *Pseudocyphellaria granulate* (C. Bab.) Malme | 45 | 8 | 7 |
| *Pseudocyphellaria hirsuta* (Mont.) Malme | 40 | 9 | 5 |
| *Pseudocyphellaria intricata* (Delise) Vain. | 22 | 3 | 3 |
| *Pseudocyphellaria lechleri* (Müll. Arg.) Du Rietz | 32 | 6 | 2 |
| *Pseudocyphellaria mallota* (Tuck.) H. Magn. | 12 | 4 | 2 |
| *Pseudocyphellaria norvegica* (Gyeln.) P. James | 13 | 5 | 4 |
| *Pseudocyphellaria nudata* (Zahlbr.) D.J. Galloway | 2 | 1 | 1 |
| *Pseudocyphellaria piloselloides* (Räsänen) H. Magn. | 6 | 6 | 1 |
| *Pseudocyphellaria scabrosa* (R. Sant.) D.J. Galloway & de Lange | 34 | 7 | 3 |
| *Pseudocyphellaria* sp. *1* | 1 | 1 | 1 |
| *Pseudocyphellaria valdiviana* (Nyl.) Follmann | 7 | 2 | 4 |
| *Pseudocyphellaria wandae* D.J. Galloway | 1 | 1 | 1 |
| *Psoroma asperellum* Nyl. | 11 | 3 | 7 |
| *Psoroma hirsutulum* Nyl. | 2 | 2 | 2 |
| *Psoroma hypnorum* (Vahl) Gray | 3 | 1 | 2 |
| *Psoroma polychidioides* (Zahlbr.) P.M. Jørg. | 3 | 2 | 1 |
| *Psorophorus pholidotus* (Mont.) Elvebakk & S.G. Hong | 1 | 1 | 1 |
| *Sticta ainoae* D.J. Galloway & J. Pickering | 1 | 1 | 1 |
| *Sticta caulescens* De Not. | 5 | 1 | 1 |
| *Sticta fuliginosa* (With.) Ach. | 12 | 4 | 1 |
| *Sticta* gr. *sublimbata* | 5 | 3 | 3 |
| *Sticta hypochra* Vain. | 15 | 5 | 2 |
| *Xanthopsoroma contextum* (Stirt.) Elvebakk & S.G. Hong | 1 | 1 | 1 |
| *Xanthopsoroma soccatum* (R. Br. ex Cromb.) Elvebakk | 1 | 1 | 1 |


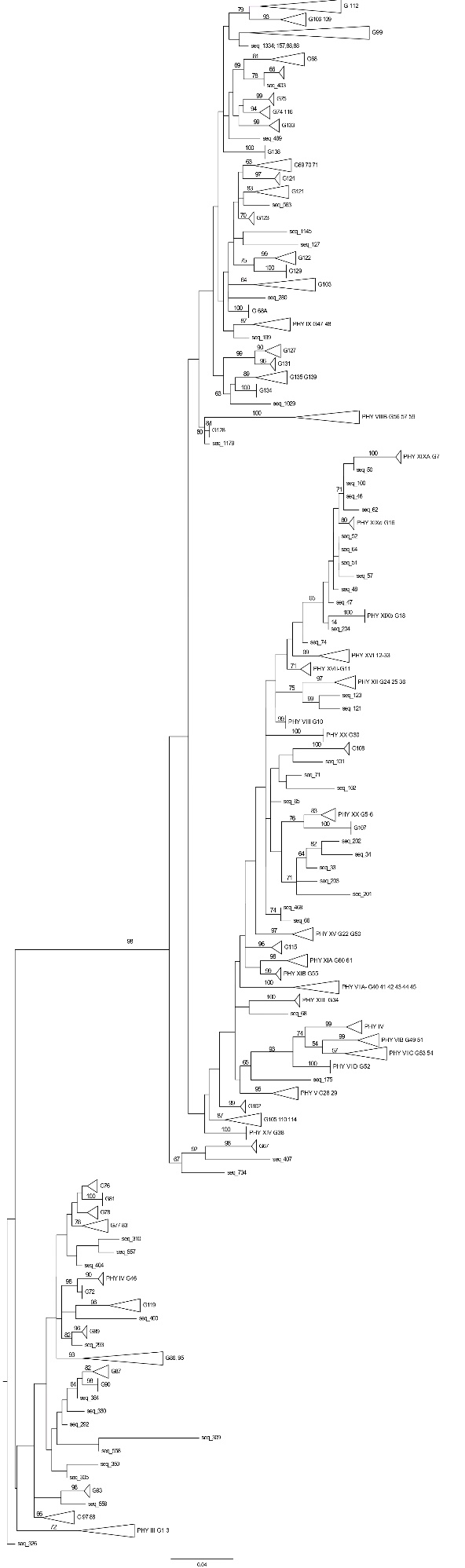


Appendix S3 Best tree obtained from Maximum likelihood analysis (with RAxML) with the definition of phylogroups. Bootstrap values are shown in the nodes. PHY: phylogroups defined by Magain et al. (2017), G: groups defined by ASAP, seq: name of sequences belonging to different phylogroups.


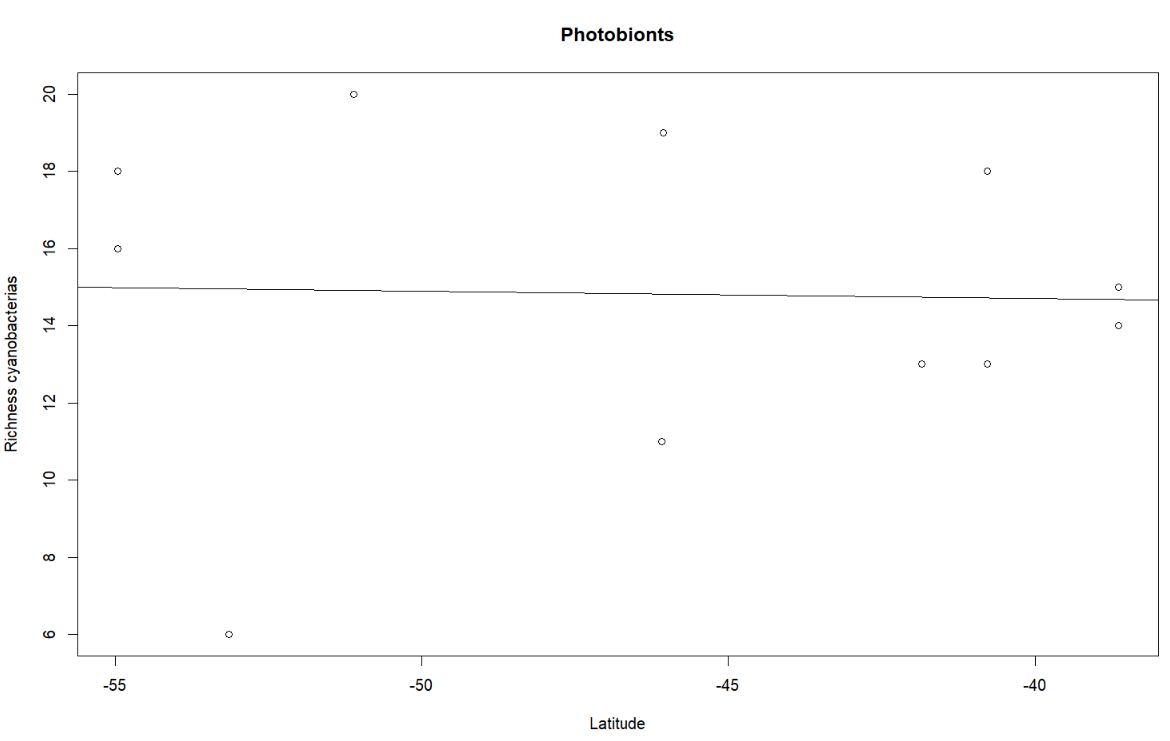


Appendix S4 Photobiont richness along the latitudinal gradient studied (R^2^ = -0.11; P = 0.93).

Appendix S5 Nostoc phylogroups, number of sequences of each phylogroups and percentage of the different phylogroups in the whole latitudinal gradient.

| **PHYLOGROUP IDENTITY** | **NUMBER OF SEQUENCES** | **PERCENTAGE** |
| --- | --- | --- |
| 1 | 1 | 0.09 |
| 2 | 18 | 1.61 |
| 3 | 8 | 0.71 |
| 4 | 24 | 2.14 |
| 5 | 2 | 0.18 |
| 6 | 1 | 0.09 |
| 7 | 23 | 2.05 |
| 8 | 2 | 0.18 |
| 9 | 1 | 0.09 |
| 10 | 3 | 0.27 |
| 11 | 10 | 0.89 |
| 12 | 13 | 1.16 |
| 13 | 3 | 0.27 |
| 14 | 5 | 0.45 |
| 15 | 1 | 0.09 |
| 16 | 1 | 0.09 |
| 17 | 3 | 0.27 |
| 18 | 1 | 0.09 |
| 19 | 1 | 0.09 |
| 20 | 1 | 0.09 |
| 21 | 30 | 2.68 |
| 22 | 39 | 3.48 |
| 23 | 2 | 0.18 |
| 24 | 9 | 0.80 |
| 25 | 2 | 0.18 |
| 26 | 1 | 0.09 |
| 27 | 1 | 0.09 |
| 28 | 2 | 0.18 |
| 29 | 1 | 0.09 |
| 30 | 1 | 0.09 |
| 31 | 1 | 0.09 |
| 32 | 2 | 0.18 |
| 33 | 1 | 0.09 |
| 34 | 3 | 0.27 |
| 35 | 66 | 5.89 |
| 36 | 1 | 0.09 |
| 37 | 5 | 0.45 |
| 38 | 418 | 37.32 |
| 39 | 2 | 0.18 |
| 40 | 21 | 1.88 |
| 41 | 1 | 0.09 |
| 42 | 216 | 19.29 |
| 43 | 30 | 2.68 |
| 44 | 1 | 0.09 |
| 45 | 1 | 0.09 |
| 46 | 3 | 0.27 |
| 47 | 1 | 0.09 |
| 48 | 28 | 2.50 |
| 49 | 25 | 2.23 |
| 50 | 2 | 0.18 |
| 51 | 9 | 0.80 |
| 52 | 27 | 2.41 |
| 53 | 3 | 0.27 |
| 54 | 19 | 1.70 |
| 55 | 1 | 0.09 |
| 56 | 5 | 0.45 |
| 57 | 4 | 0.36 |
| 58 | 2 | 0.18 |
| 59 | 5 | 0.45 |
| 60 | 1 | 0.09 |
| 61 | 1 | 0.09 |
| 62 | 3 | 0.27 |
| 63 | 1 | 0.09 |
| 64 | 1 | 0.09 |


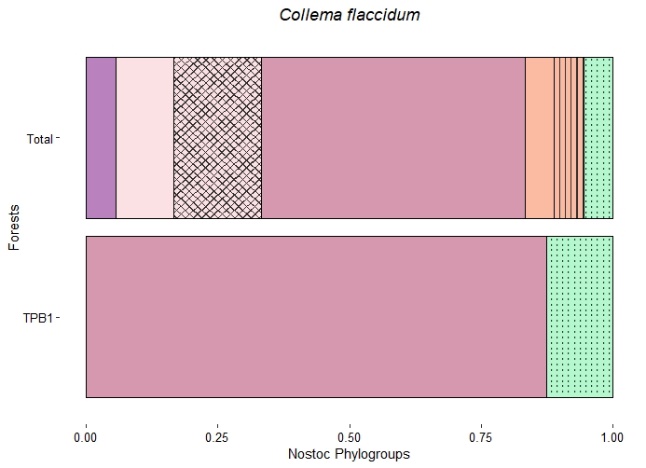

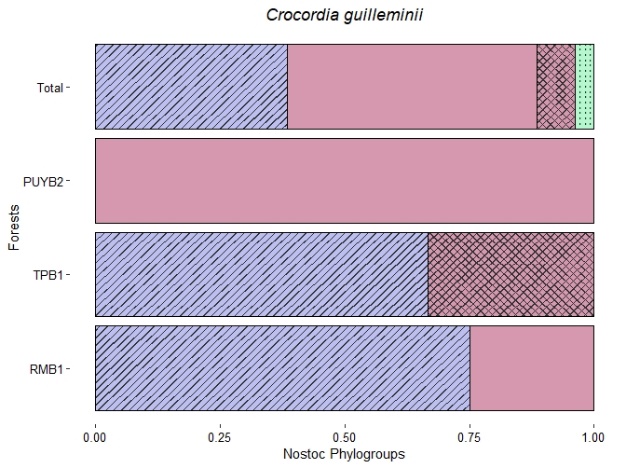

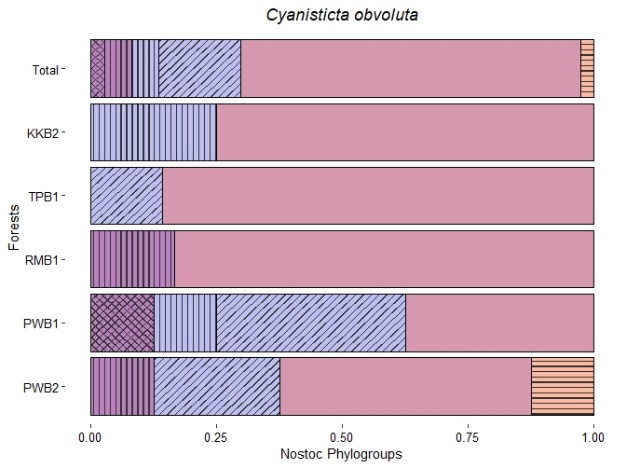

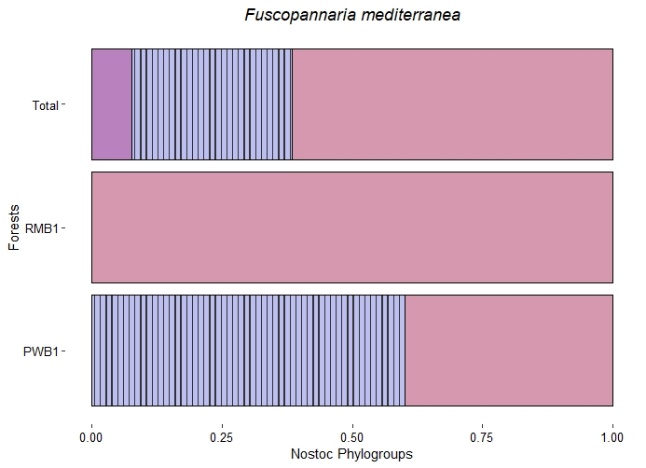


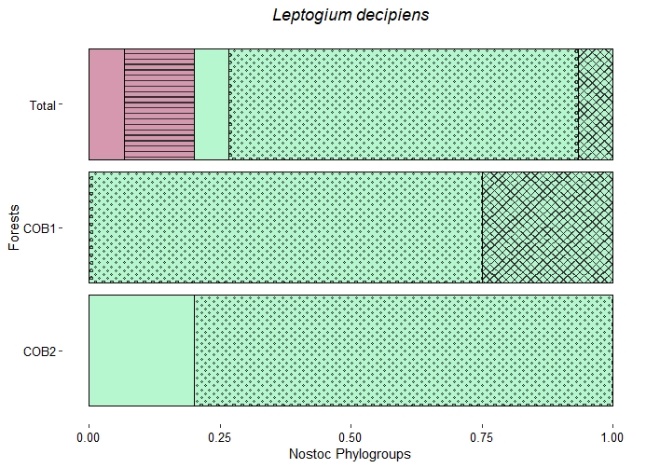

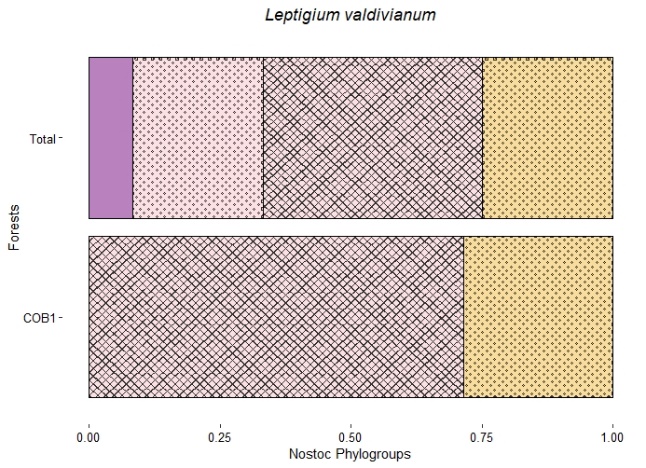

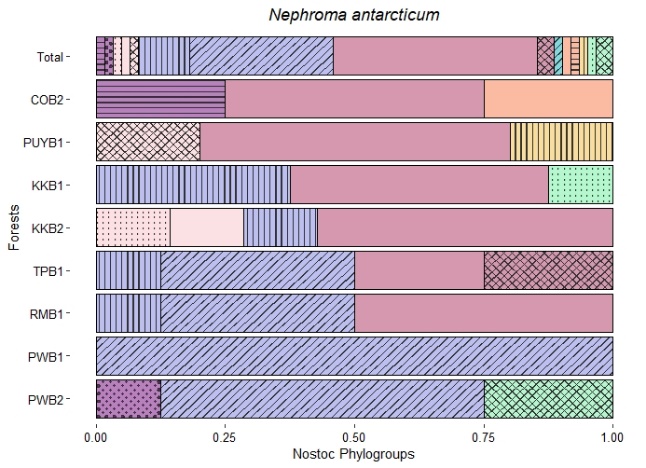

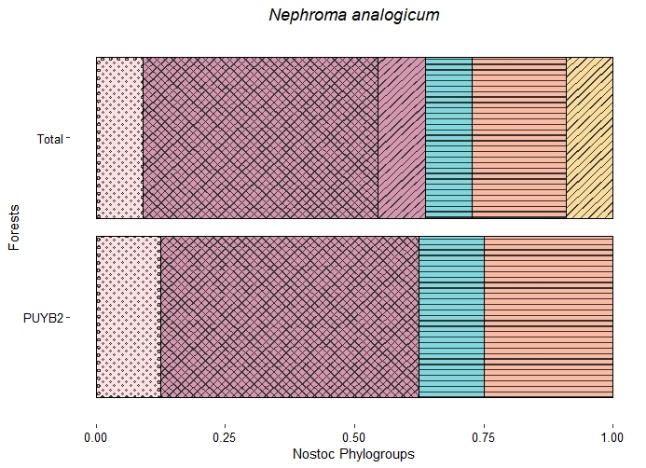


Appendix S6 Nostoc phylogroups composition for each mycobiont species in the studied forests and the total latitudinal gradient, from North (top of the graphs) to South (bottom of the graphs).


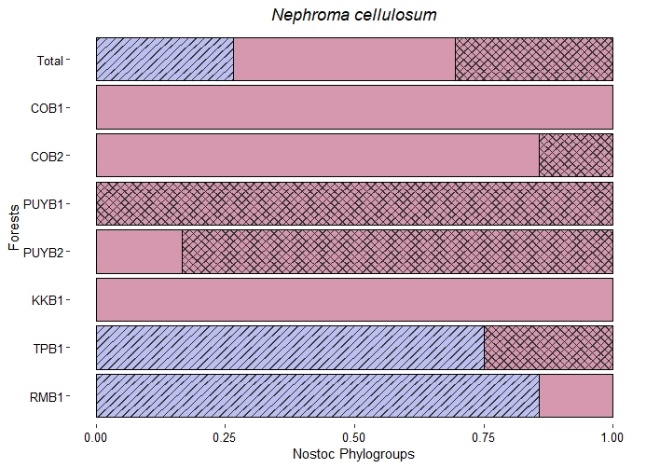

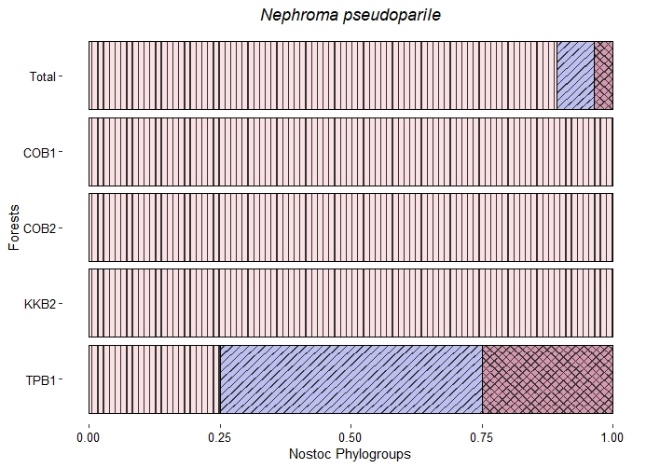


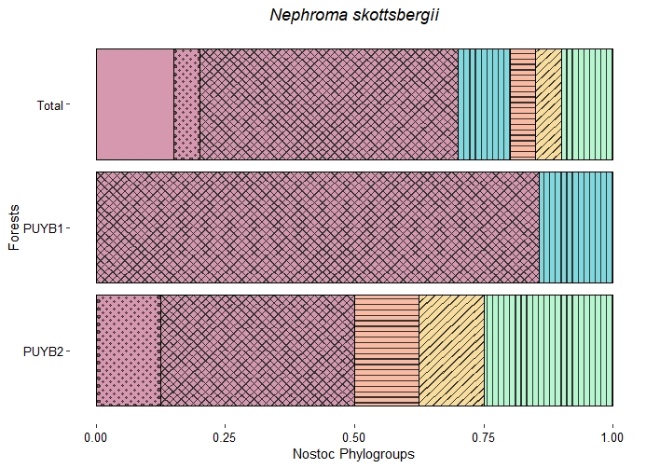

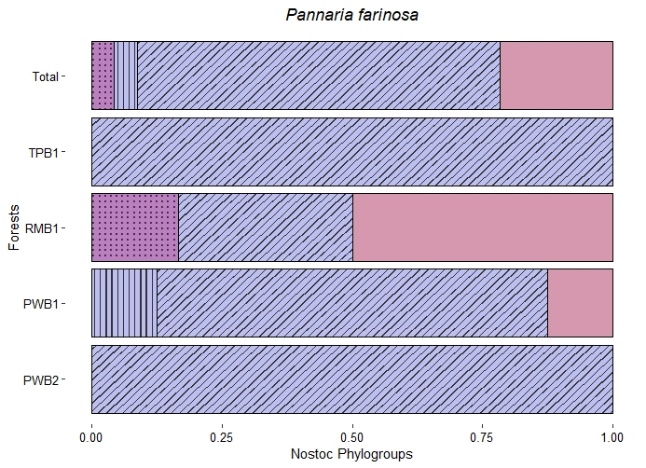

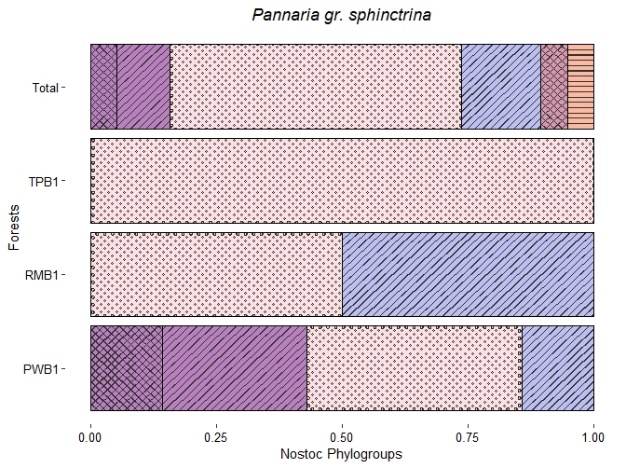

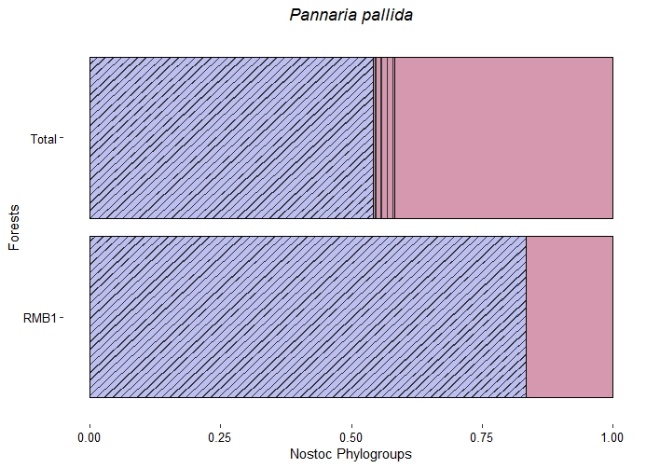

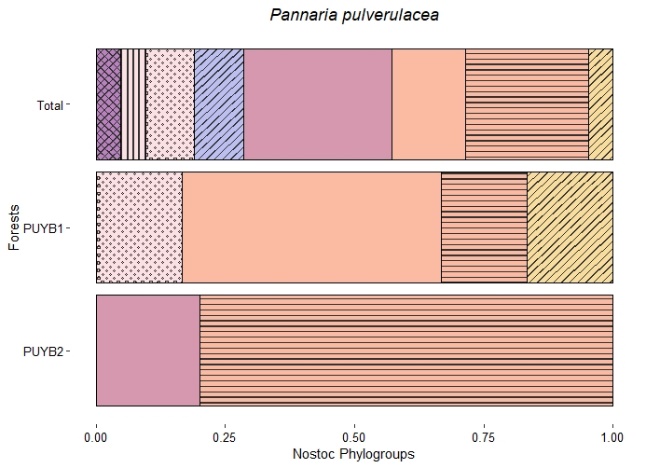

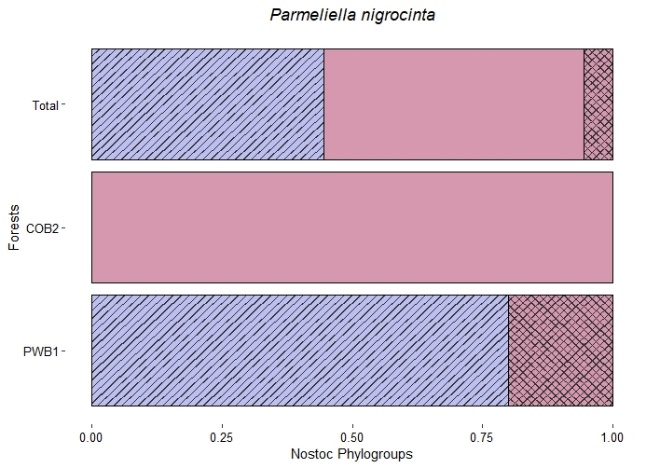


Appendix S6 Nostoc phylogroups composition for each mycobiont species in the studied forests and the total latitudinal gradient, from North (top of the graphs) to South (bottom of the graphs) (cont.).


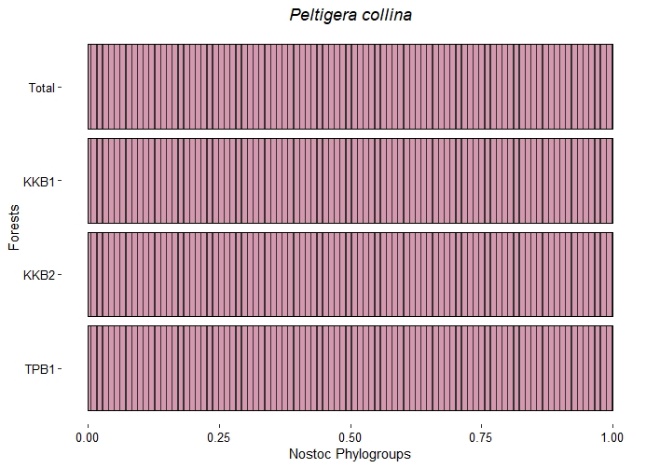

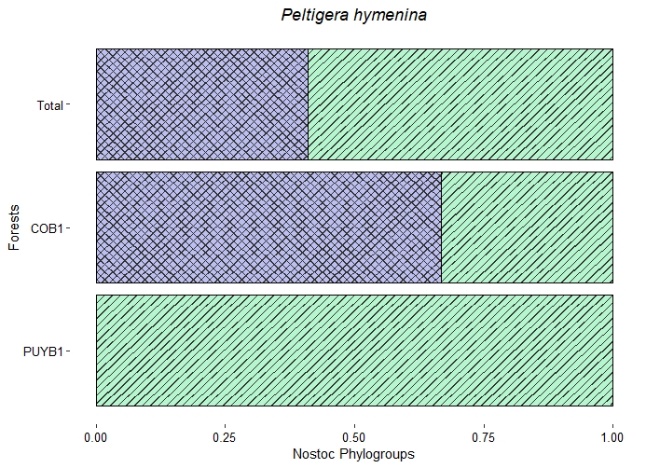

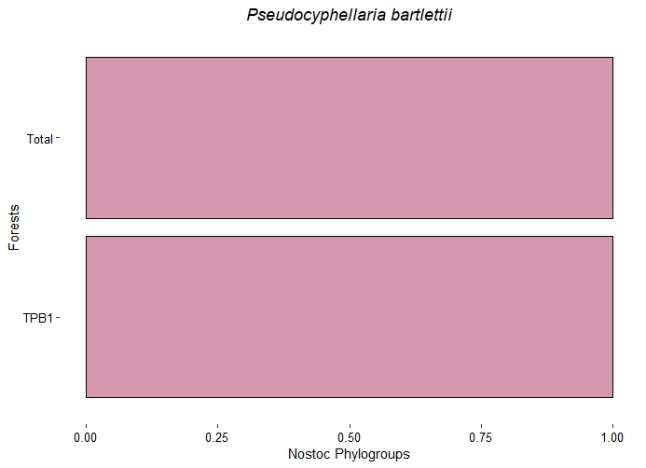

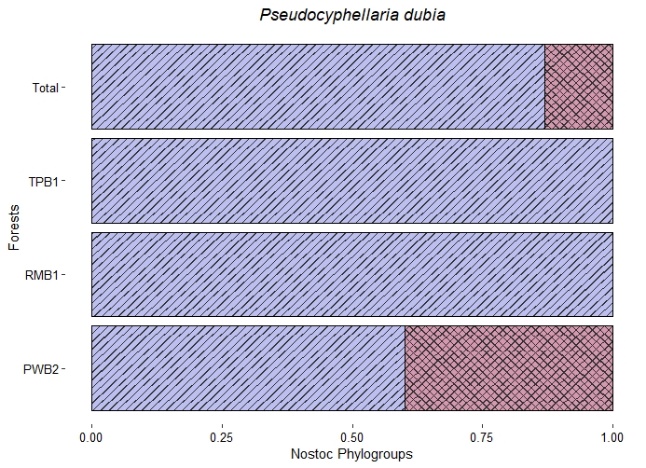


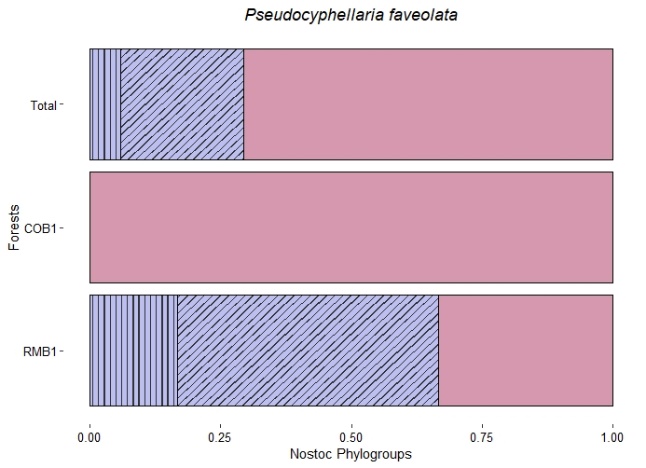

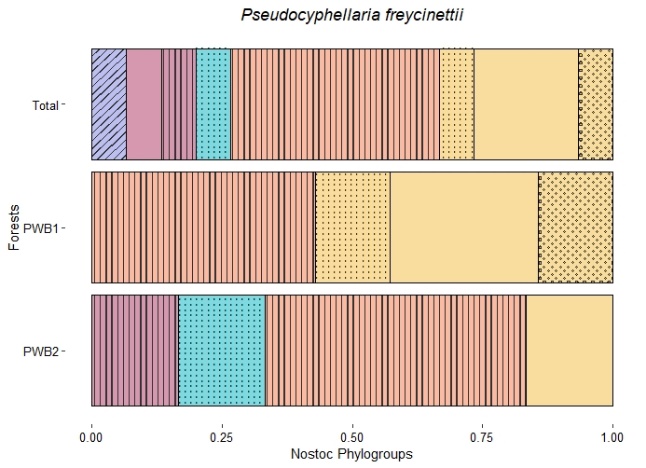

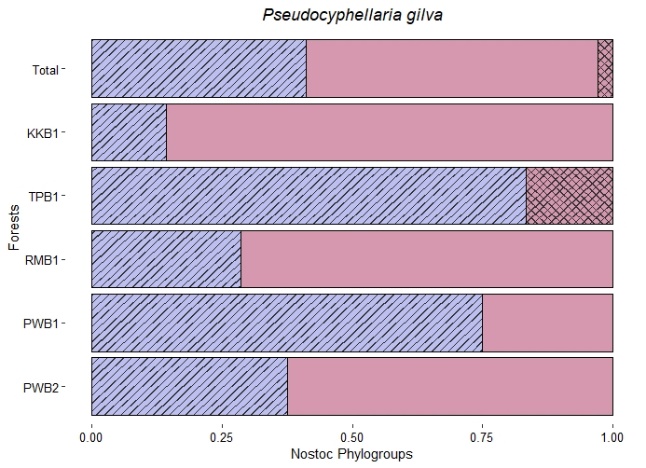

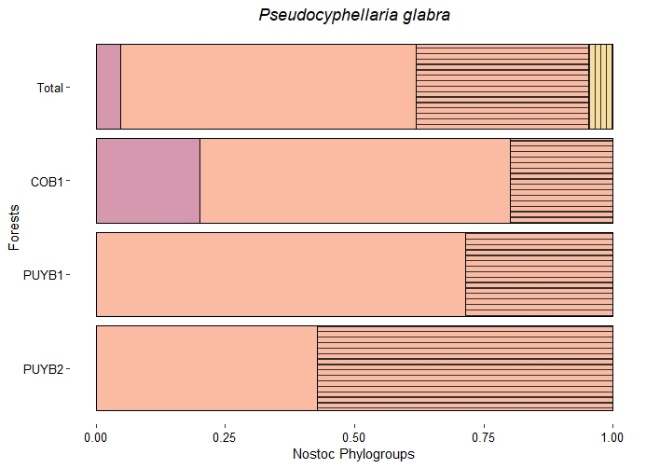


Appendix S6 Nostoc phylogroups composition for each mycobiont species in the studied forests and the total latitudinal gradient, from North (top of the graphs) to South (bottom of the graphs) (cont.).


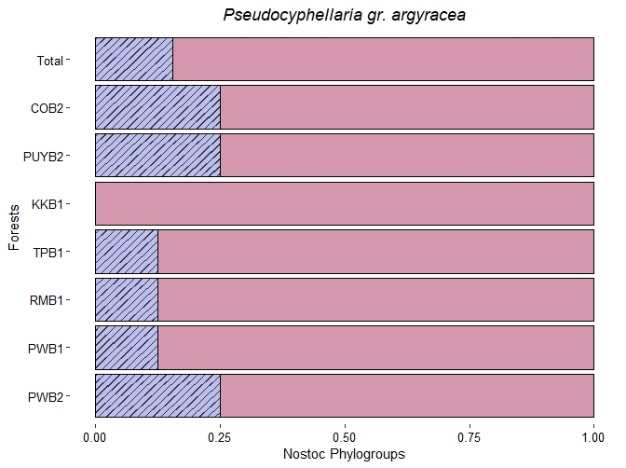

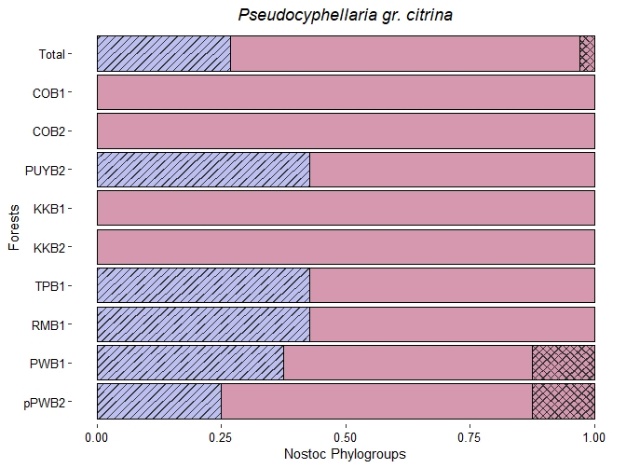

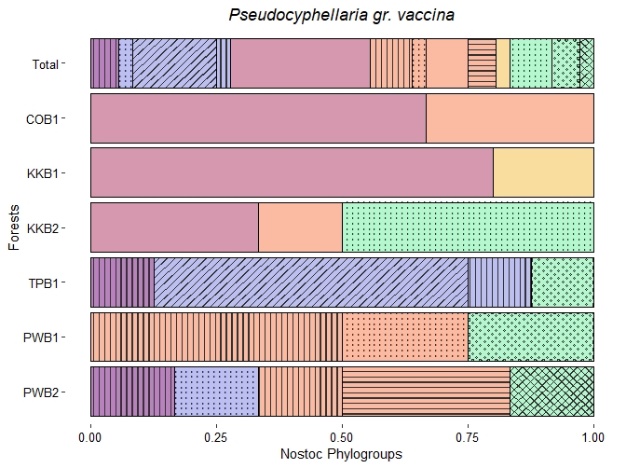

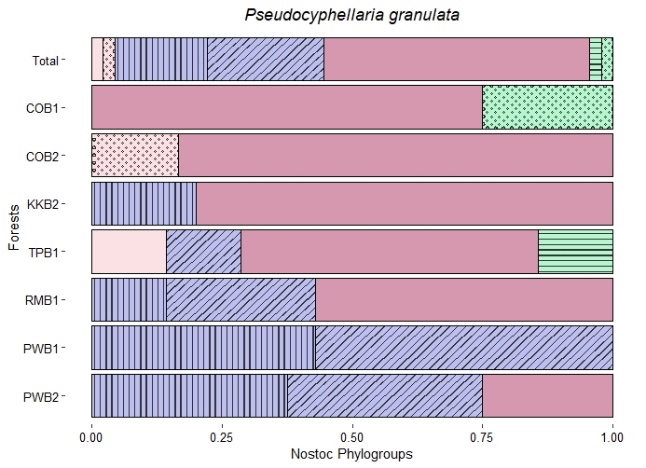

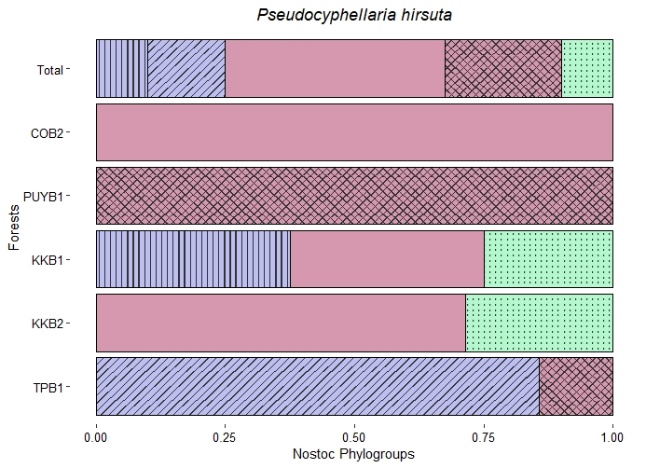

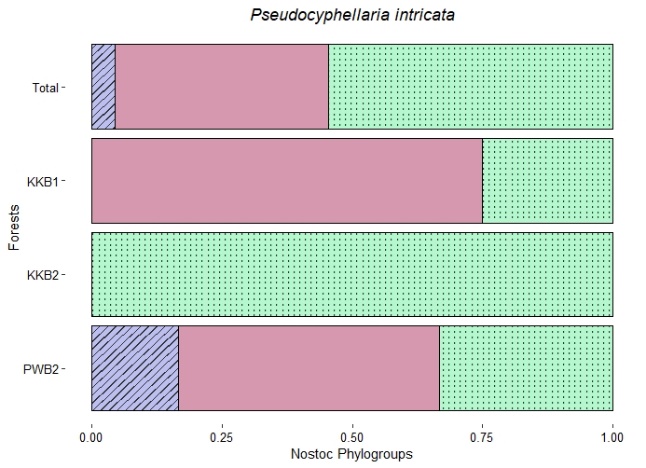

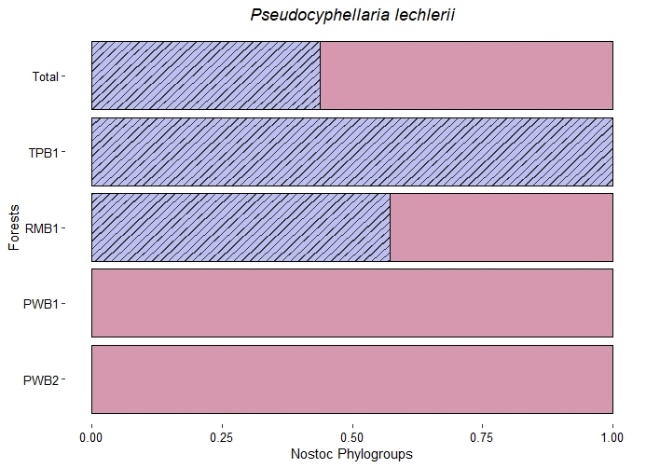

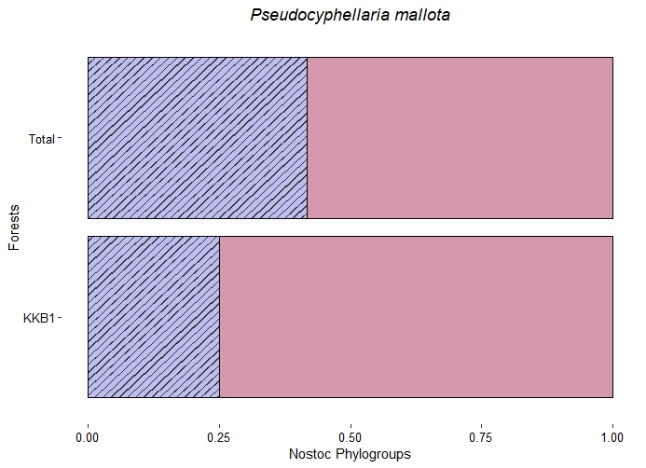


Appendix S6 Nostoc phylogroups composition for each mycobiont species in the studied forests and the total latitudinal gradient, from North (top of the graphs) to South (bottom of the graphs) (cont.).


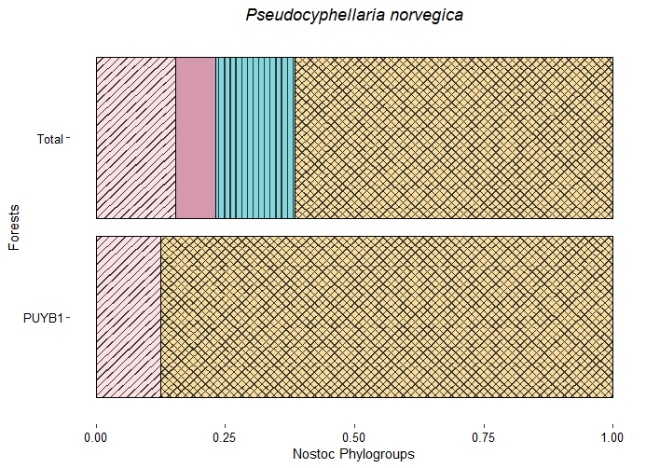

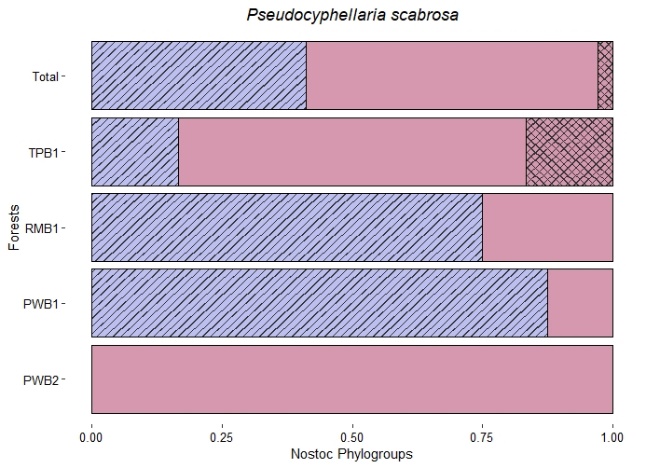


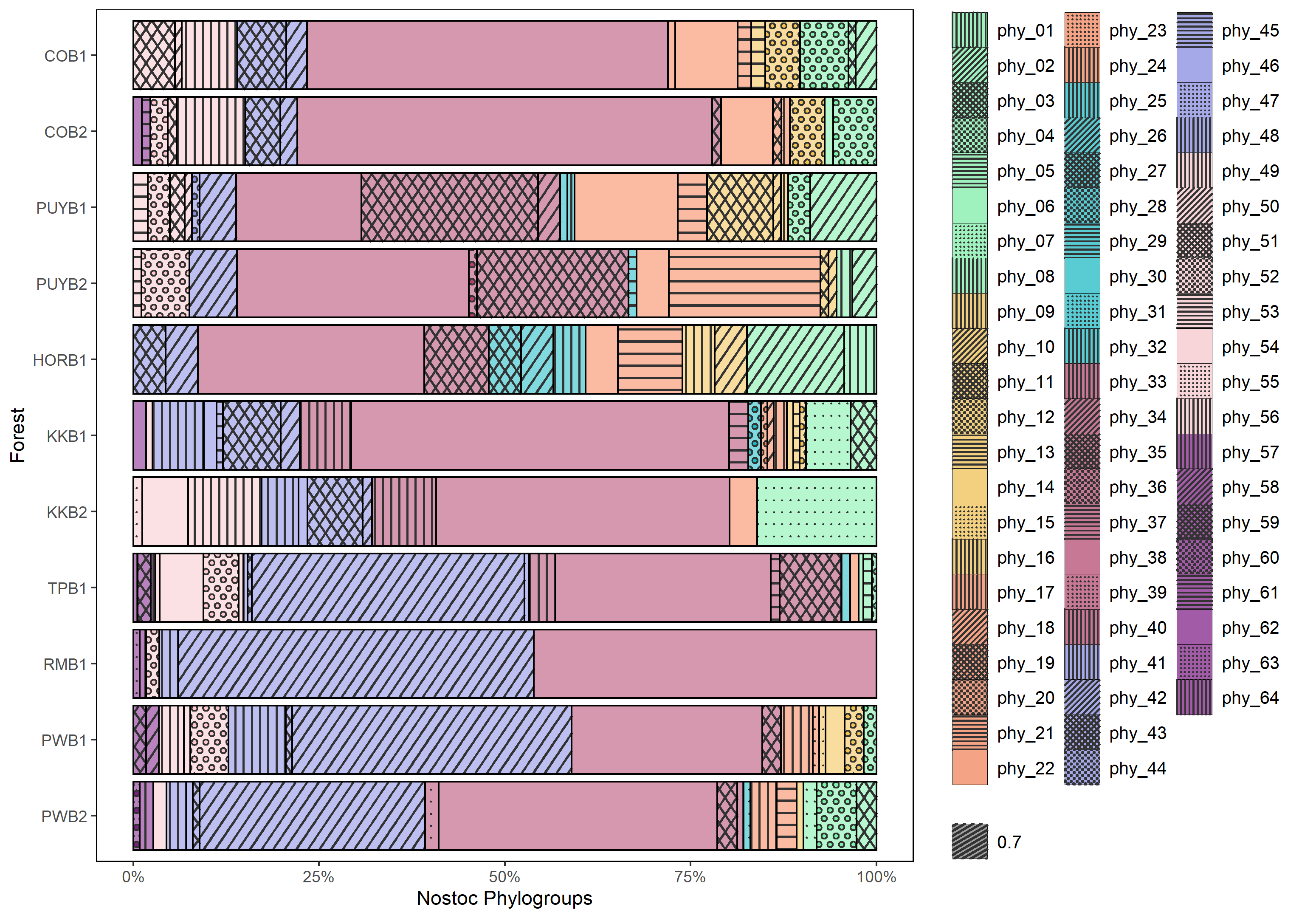

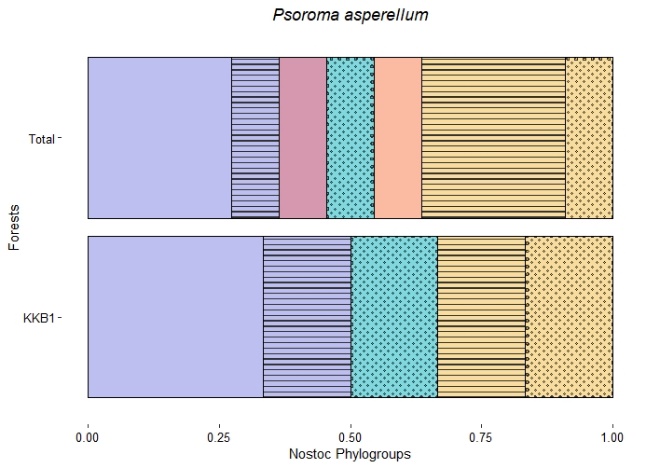

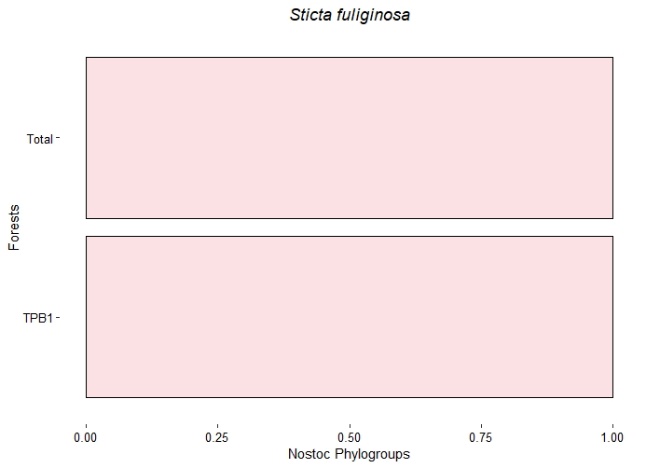

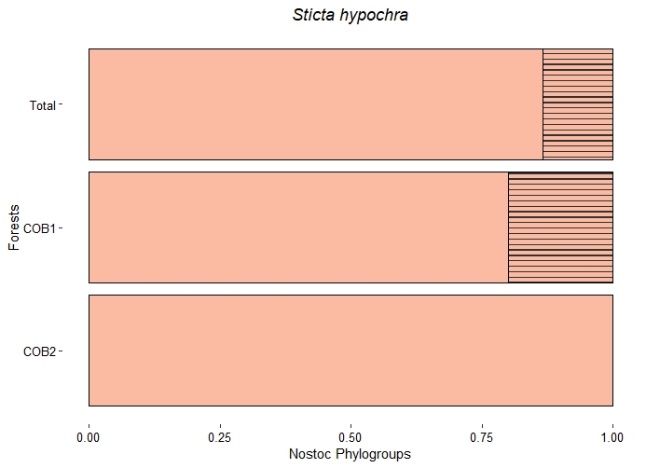


Appendix S6 Nostoc phylogroups composition for each mycobiont species in the studied forests and the total latitudinal gradient, from North (top of the graphs) to South (bottom of the graphs) (cont.).


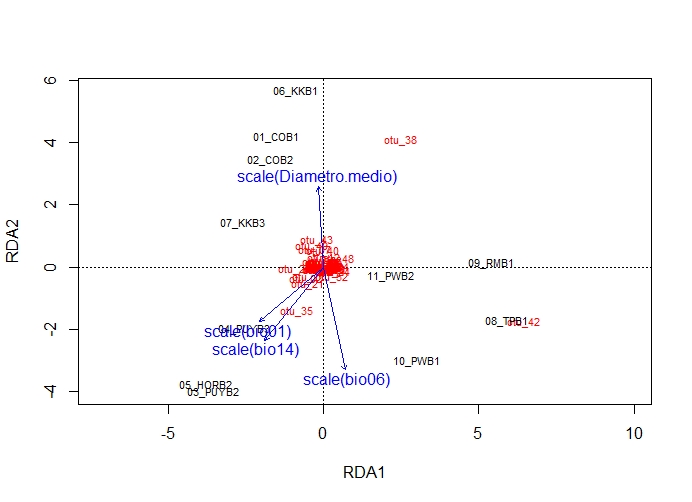


Appendix S7 Redundancy Analysis (RDA) of Nostoc phylogroups composition and environmental variables ((DBH, Mean Annual Temperature - bio01, Minimum Temperature of the Coldest Month - bio06, and Precipitation of the Driest Month - bio14).


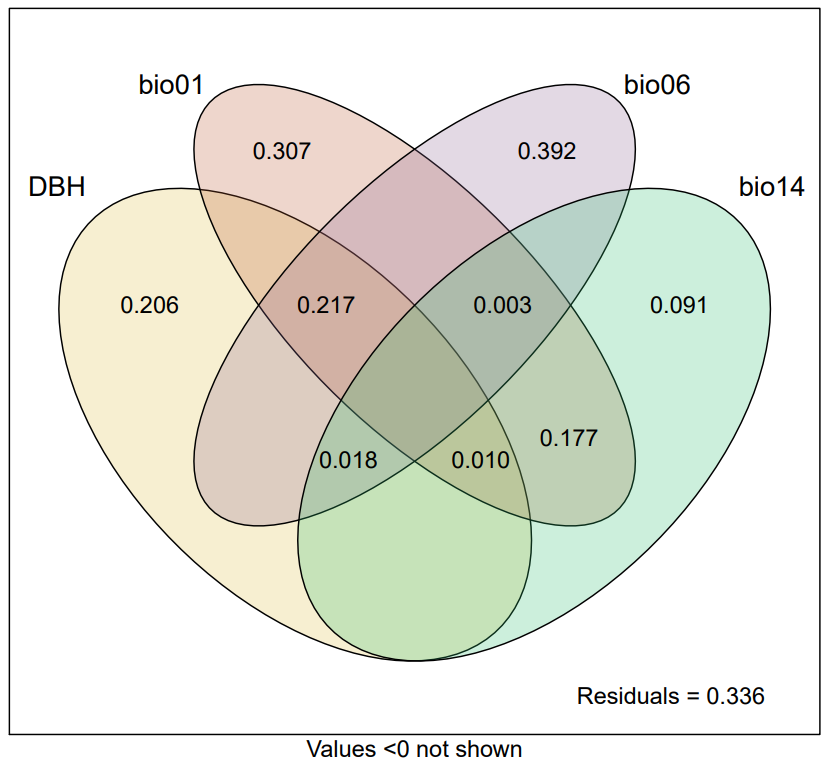


Appendix S8 Venn diagrams showing the variation partitioning of the photobiont composition explained by each group of explanatory variables (DBH, Mean Annual Temperature - bio01, Minimum Temperature of the Coldest Month - bio06, and Precipitation of the Driest Month - bio14).

Suppl. 9 Mycobiont species accumulation curves for the number of interacting cyanobionts (partner richness).


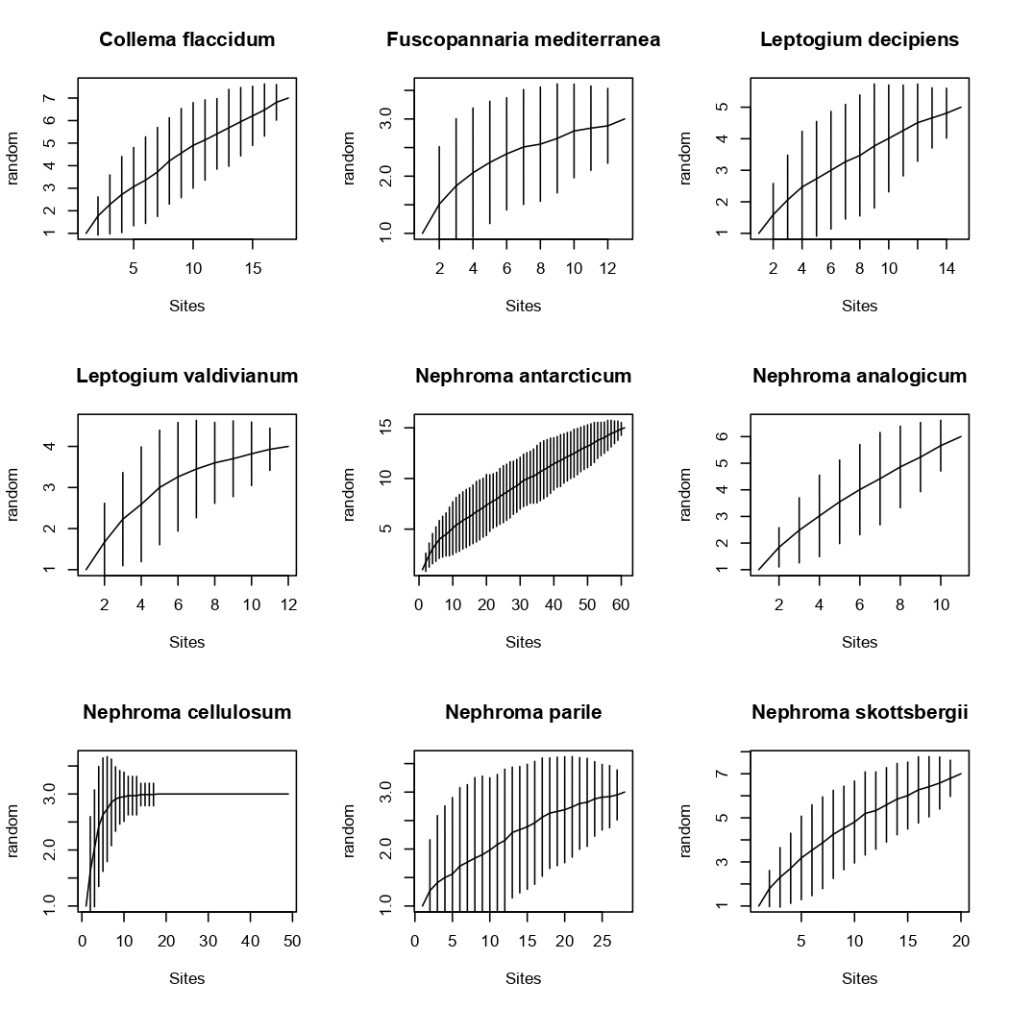

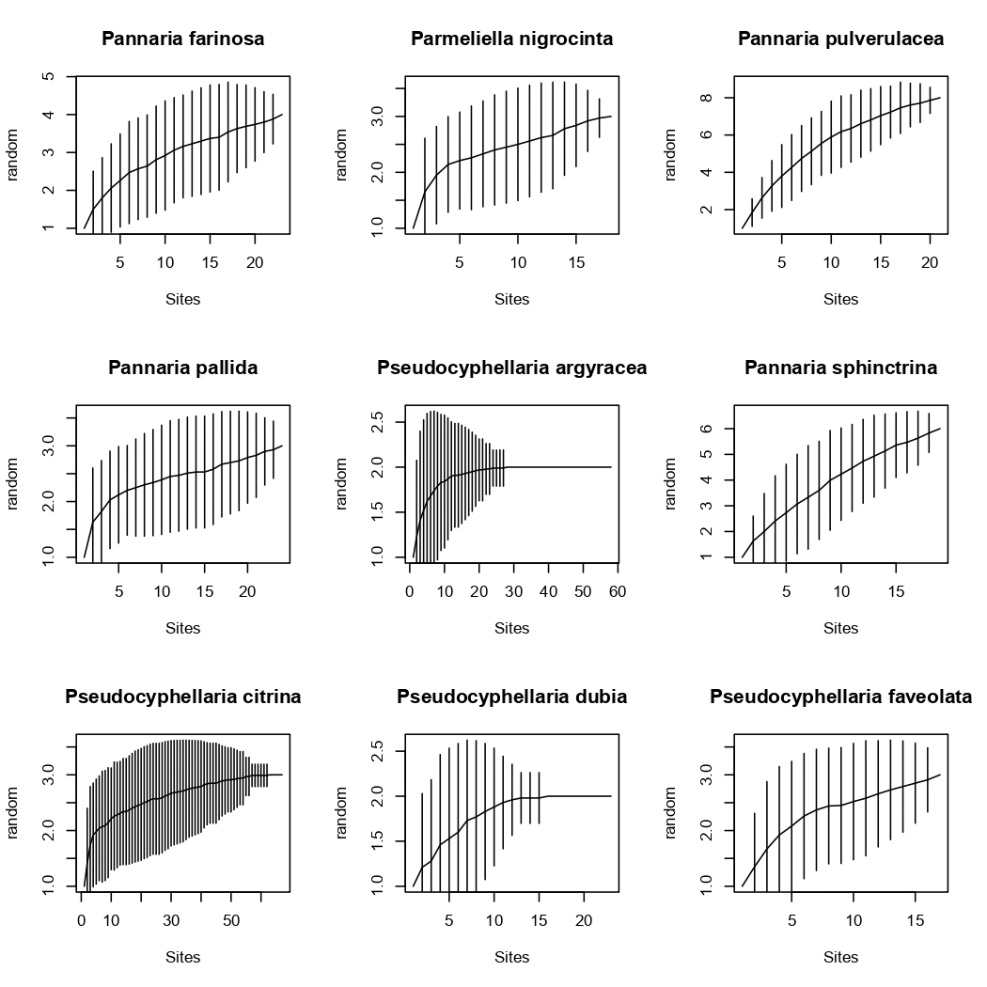

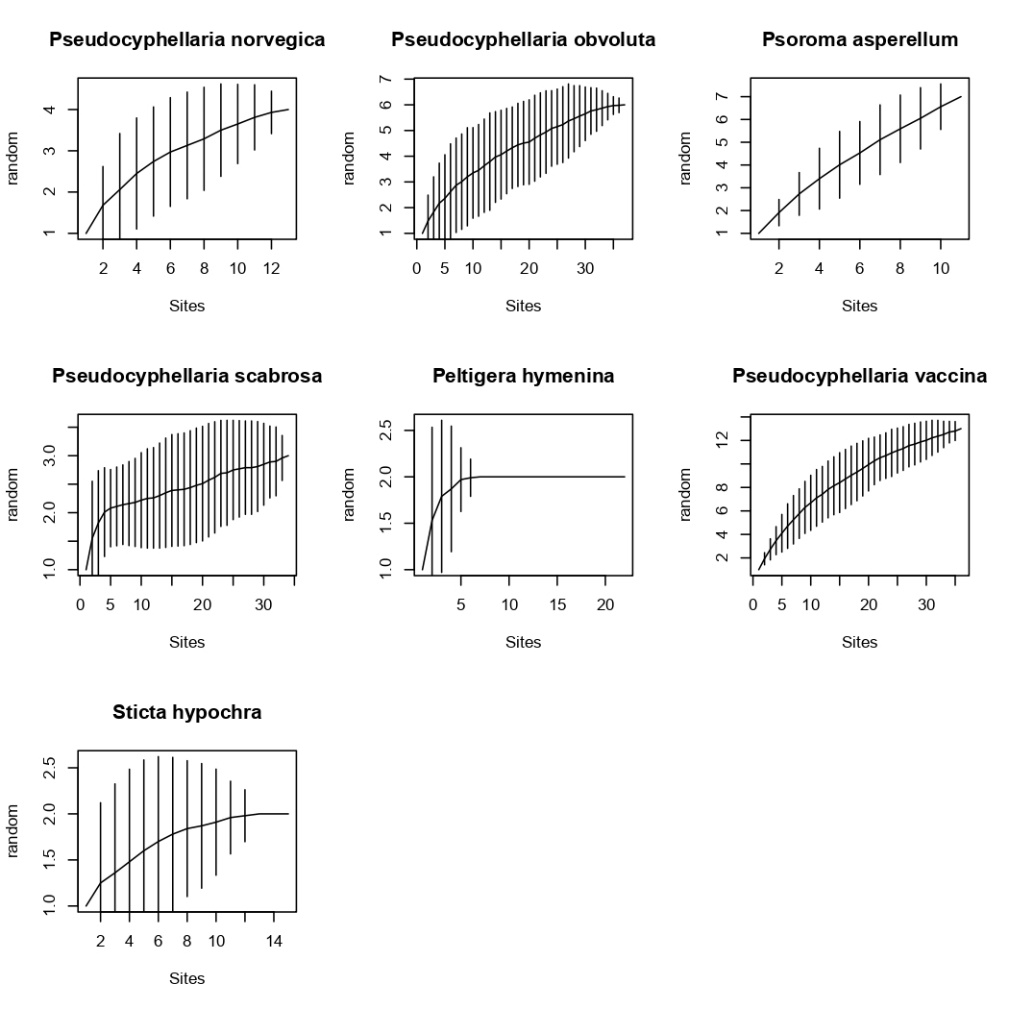

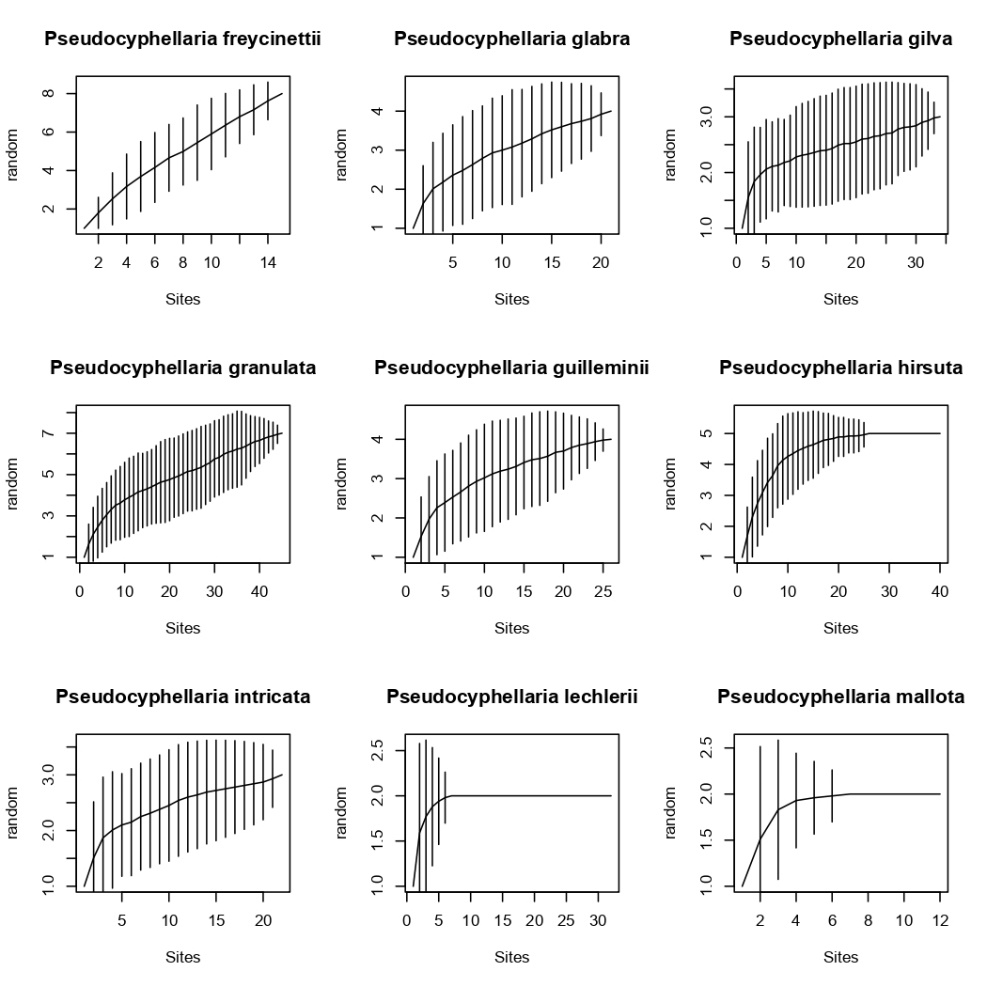


Suppl. 9 Mycobiont species accumulation curves for the number of interacting cyanobionts (partner richness) (cont.).


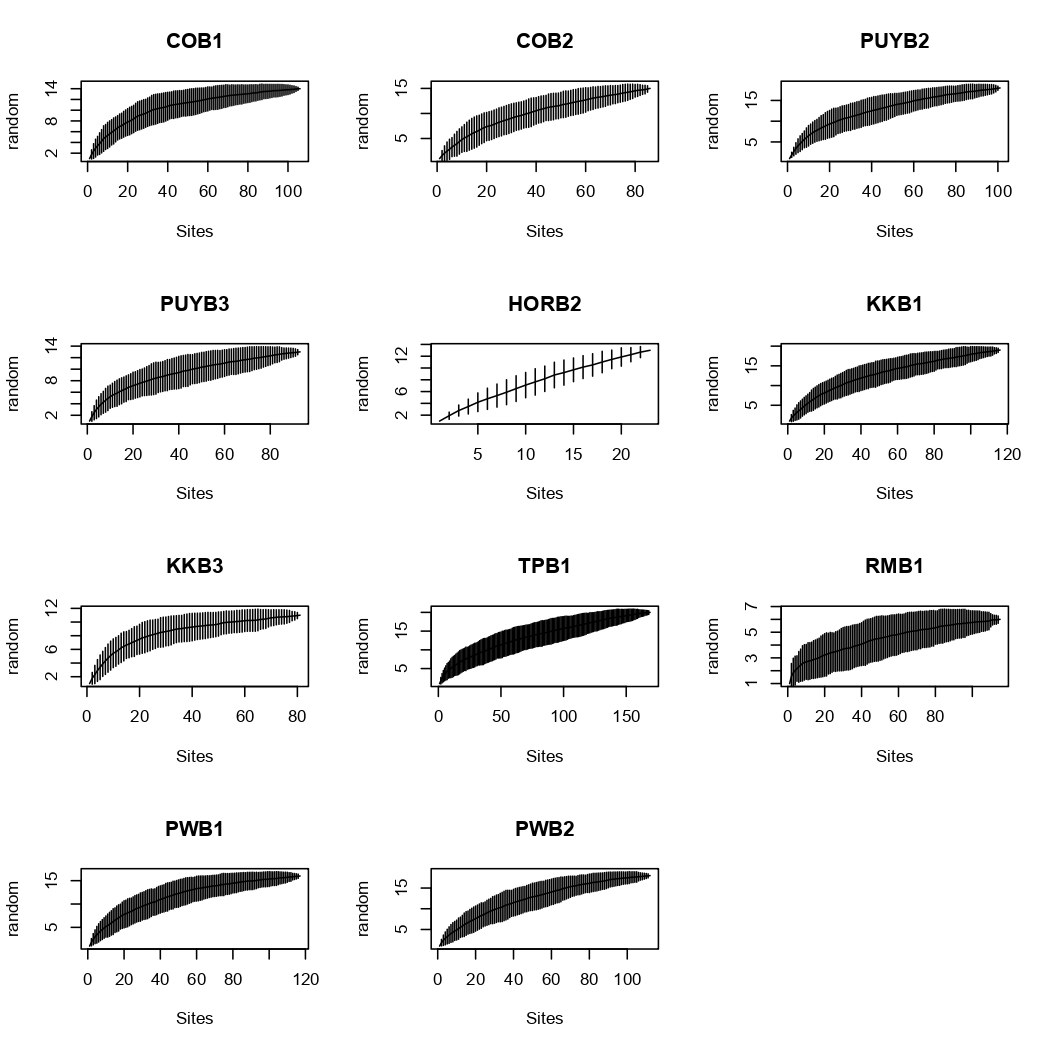


Appendix S10 Forest accumulation curves for the Nostoc phylogroups.

Appendix S11 Number of Nostoc phylogroups observed for those mycobiont species with at least 10 sequenced thalli (PARTNER RICHNESS), richness estimated with Chao1 (S.CHAO1), standard deviation of Chao1 (SE.CHAO1), estimated percentage sampled comparing S.OBS and S. CHAO1 (% SAMPLED).

| **SPECIE** | **PARTNER RICHNESS** | **S.CHAO1** | **SE.CHAO1** | **% SAMPLED** |
| --- | --- | --- | --- | --- |
| *Collema flaccidum* | 7 | 10 | 4.11 | 70 |
| *Crocodia guilleminii* | 4 | 4 | 0.41 | 100 |
| *Cyanisticta obvoluta* | 6 | 6.33 | 0.41 | 94.74 |
| *Fuscopannaria mediterranea* | 3 | 3 | 0.41 | 100 |
| *Leptogium decipiens* | 5 | 6.5 | 2.53 | 76.92 |
| *Leptogium valdivianum* | 4 | 4 | 0.43 | 100 |
| *Nephroma analogicum* | 6 | 9 | 12.80 | 66.67 |
| *Nephroma antarcticum* | 15 | 30 | 4.10 | 50 |
| *Nephroma cellulosum* | 3 | 3 | 0.00 | 100 |
| *Nephroma pseudoparile* | 3 | 3 | 0.20 | 100 |
| *Nephroma skottsbergii* | 7 | 8 | 1.79 | 87.50 |
| *Pannaria farinosa* | 4 | 5 | 2.17 | 80 |
| *Pannaria gr. sphinctrina* | 6 | 7.5 | 0.41 | 80 |
| *Pannaria pallida* | 3 | 3 | 0.41 | 100 |
| *Pannaria pulverulacea* | 8 | 9 | 1.80 | 88.89 |
| *Parmeliella nigrocinta* | 3 | 3 | 0.00 | 100 |
| *Peltigera collina* | 1 | 1 | 2.54 | 100 |
| *Peltigera hymenina* | 2 | 2 | 0.00 | 100 |
| *Pseudocyphellaria bartlettii* | 1 | 1 | 0.00 | 100 |
| *Pseudocyphellaria dubia* | 2 | 2 | 13.51 | 100 |
| *Pseudocyphellaria faveolata* | 3 | 3 | 2.17 | 100 |
| *Pseudocyphellaria freycinettii* | 8 | 23 | 0.41 | 34.78 |
| *Pseudocyphellaria gilva* | 3 | 3 | 7.08 | 100 |
| *Pseudocyphellaria glabra* | 4 | 5 | 0.22 | 80 |
| *Pseudocyphellaria* gr. *argyracea* | 2 | 2 | 0.00 | 100 |
| *Pseudocyphellaria* gr. *citrina* | 3 | 3 | 0.41 | 100 |
| *Pseudocyphellaria* gr. *vaccina* | 13 | 15.5 | 0.00 | 83.87 |
| *Pseudocyphellaria granulata* | 7 | 13 | 0.00 | 53.85 |
| *Pseudocyphellaria hirsuta* | 5 | 5 | 0.00 | 100 |
| *Pseudocyphellaria intricata* | 3 | 3 | 0.00 | 100 |
| *Pseudocyphellaria lechlerii* | 2 | 2 | 0.14 | 100 |
| *Pseudocyphellaria mallota* | 2 | 2 | 0.91 | 100 |
| *Pseudocyphellaria norvegica* | 4 | 4 | 10.10 | 100 |
| *Pseudocyphellaria scabrosa* | 3 | 3 | 0.00 | 100 |
| *Psoroma asperellum* | 7 | 17 | 3.15 | 41.18 |
| *Sticta fuliginosa* | 1 | 1 | 0.00 | 100 |
| *Sticta hypochra* | 2 | 2 | 0 | 100 |

Appendix S12 Mycobiont species richness per forest (MYCOBIONT SPS.), Nostoc phylogroup richness observed per forest (S.OBS), richness estimated with Chao1 per forest (S.CHAO1), standard deviation of Chao1 (SE.CHAO1).

| **BOSQUE** | **MYCOBIONT SPS** | **S.OBS** | **S.CHAO1** | **SE.CHAO1** |
| --- | --- | --- | --- | --- |
| COB1 | 37 | 14 | 15 | 1.81 |
| COB2 | 24 | 15 | 22 | 7.08 |
| PUYB1 | 35 | 18 | 23 | 5.51 |
| PUYB2 | 25 | 13 | 18 | 5.98 |
| HORB1 | 16 | 13 | 25 | 10.73 |
| KKB1 | 30 | 19 | 23.2 | 4.33 |
| KKB2 | 22 | 11 | 14 | 4.51 |
| TPB1 | 36 | 20 | 27.2 | 6.43 |
| RMB1 | 24 | 6 | 6.5 | 1.27 |
| PWB1 | 25 | 16 | 17.5 | 2.22 |
| PWB2 | 23 | 18 | 21 | 3.41 |

Appendix S13 Mycobiont specialization measures, reproductive mode and function of cyanobiont per forest. Specialization metrics were estimated for those mycobiont species with at least ten thalli along the latitudinal gradient and in those forests in which they had a minimum of four thalli. N thalli: Number of thalli sequenced per species and forest, Partner richness: number of Nostoc phylogroups found per species and forest, Simpson index, d' index, reproductive mode, and function of the cyanobiont. Abbreviations of the forests: CO, Conguillío National Park; PUY, Puyehue National Park; HOR, Hornopirén National Park; KK, Cerro Castillo National Park; TP, Torres del Paine National Park; RM, Magallanes National Reserve; and PW, Navarino Island. B1 and B2 refers to forest 1 or 2, respectively.

| **SPECIES** | **FOREST** | **N THALLI** | **PARTNER RICHNESS** | **SIMPSON INDEX** | **d’ INDEX** | **REPRODUCTIVE MODE** | **FUNCTION OF THE CYANOBIONT** |
| --- | --- | --- | --- | --- | --- | --- | --- |
| ***Collema*** | | | | | | | |
| *C. flaccidum* | TPB1 | 8 | 2 | 0.78 | 0.39 | asexual | cyanolichen |
| ***Crocordia*** |  |  |  |  |  |  |  |
| *C. guilleminii* | PUYB2 | 6 | 1 | 1.00 | 0.36 | Sexual | cyanolichen |
| *C. guilleminii* | TPB1 | 6 | 2 | 0.56 | 0.18 | Sexual | cyanolichen |
| *C. guilleminii* | RMB1 | 8 | 2 | 0.63 | 0.05 | Sexual | cyanolichen |
| ***Cyanisticta*** |  |  |  |  |  |  |  |
| *C. obvoluta* | KKB2 | 4 | 2 | 0.63 | 0.17 | Sexual | cephalolichen |
| *C. obvoluta* | TPB1 | 7 | 2 | 0.76 | 0.18 | Sexual | cephalolichen |
| *C. obvoluta* | RMB1 | 6 | 2 | 0.72 | 0.32 | Sexual | cephalolichen |
| *C. obvoluta* | PWB1 | 8 | 4 | 0.31 | 0.06 | Sexual | cephalolichen |
| *C. obvoluta* | PWB2 | 8 | 4 | 0.34 | 0.09 | Sexual | cephalolichen |
| ***Fuscopannaria*** | | | | | | | |
| *F. mediterranea* | RMB1 | 4 | 1 | 1.00 | 0.22 | sexual | cyanolichen |
| *F. mediterranea* | PWB1 | 5 | 2 | 0.52 | 0.37 | sexual | cyanolichen |
| ***Leptogium*** | | | | | | | |
| *L. decipiens* | COB1 | 4 | 2 | 0.63 | 0.76 | asexual | cyanolichen |
| *L. decipiens* | COB2 | 5 | 2 | 0.68 | 0.93 | asexual | cyanolichen |
| *L. valdivianum* | COB1 | 7 | 2 | 0.59 | 0.84 | sexual | cyanolichen |
| ***Nephroma*** | | | | | | | |
| *N. analogicum* | PUYB2 | 8 | 4 | 0.34 | 0.30 | asexual | cephalolichen |
| *N. antarcticum* | COB2 | 4 | 3 | 0.38 | 0.22 | asexual | cephalolichen |
| *N. antarcticum* | PUYB1 | 5 | 3 | 0.44 | 0.55 | sexual | cephalolichen |
| *N. antarcticum* | KKB1 | 8 | 3 | 0.41 | 0.18 | both | cephalolichen |
| *N. antarcticum* | KKB2 | 7 | 4 | 0.39 | 0.27 | both | cephalolichen |
| *N. antarcticum* | TPB1 | 8 | 4 | 0.28 | 0.14 | both | cephalolichen |
| *N. antarcticum* | RMB1 | 8 | 3 | 0.41 | 0.03 | sexual | cephalolichen |
| *N. antarcticum* | PWB1 | 6 | 1 | 1.00 | 0.24 | sexual | cephalolichen |
| *N. antarcticum* | PWB2 | 8 | 3 | 0.47 | 0.44 | sexual | cephalolichen |
| *N. cellulosum* | COB1 | 6 | 1 | 1.00 | 0.12 | sexual | cyanolichen |
| *N. cellulosum* | COB2 | 7 | 2 | 0.76 | 0.19 | both | cyanolichen |
| *N. cellulosum* | PUYB1 | 6 | 1 | 1.00 | 0.44 | both | cyanolichen |
| *N. cellulosum* | PUYB2 | 6 | 2 | 0.72 | 0.32 | both | cyanolichen |
| *N. cellulosum* | KKB1 | 5 | 1 | 1.00 | 0.07 | both | cyanolichen |
| *N. cellulosum* | TPB1 | 8 | 2 | 0.63 | 0.20 | sexual | cyanolichen |
| *N. cellulosum* | RMB1 | 7 | 2 | 0.76 | 0.10 | sexual | cyanolichen |
| *N. pseudoparile* | COB1 | 8 | 1 | 1.00 | 1.00 | both | cyanolichen |
| *N. pseudoparile* | COB2 | 8 | 1 | 1.00 | 1.00 | asexual | cyanolichen |
| *N. pseudoparile* | KKB2 | 8 | 1 | 1.00 | 1.00 | asexual | cyanolichen |
| *N. pseudoparile* | TPB1 | 4 | 3 | 0.38 | 0.29 | asexual | cyanolichen |
| *N. skottsbergii* | PUYB1 | 7 | 2 | 0.76 | 0.49 | asexual | cephalolichen |
| *N. skottsbergii* | PUYB2 | 8 | 5 | 0.25 | 0.53 | both | cephalolichen |
| ***Pannaria*** | | | | | | | |
| *P. farinosa* | TPB1 | 4 | 1 | 1.00 | 0.18 | asexual | cephalolichen |
| *P. farinosa* | RMB1 | 6 | 3 | 0.39 | 0.12 | asexual | cephalolichen |
| *P. farinosa* | PWB1 | 8 | 3 | 0.59 | 0.08 | asexual | cephalolichen |
| *P. farinosa* | PWB2 | 4 | 1 | 1.00 | 0.27 | asexual | cephalolichen |
| *P. gr. sphinctrina* | TPB1 | 6 | 1 | 1.00 | 0.91 | sexual | cephalolichen |
| *P. gr. sphinctrina* | RMB1 | 4 | 2 | 0.50 | 0.50 | sexual | cephalolichen |
| *P. gr. sphinctrina* | PWB1 | 7 | 4 | 0.31 | 0.62 | sexual | cephalolichen |
| *P. pallida* | RMB1 | 6 | 2 | 0.72 | 0.08 | sexual | cephalolichen |
| *P. pulverulacea* | PUYB1 | 6 | 4 | 0.33 | 0.52 | both | cephalolichen |
| *P. pulverulacea* | PUYB2 | 5 | 2 | 0.68 | 0.26 | asexual | cephalolichen |
| ***Parmeliella*** | | | | | | | |
| *P. nigrocinta* | COB2 | 4 | 1 | 1.00 | 0.04 | sexual | cyanolichen |
| *P. nigrocinta* | PWB1 | 5 | 2 | 0.68 | 0.23 | sexual | cyanolichen |
| ***Peltigera*** | | | | | | | |
| *P. collina* | KKB1 | 8 | 1 | 1.00 | 1.00 | asexual | cyanolichen |
| *P. collina* | KKB2 | 7 | 1 | 1.00 | 1.00 | asexual | cyanolichen |
| *P. collina* | TPB1 | 5 | 1 | 1.00 | 0.94 | Asexual | cyanolichen |
| *P. hymenina* | COB1 | 6 | 2 | 0.56 | 0.79 | Sexual | cyanolichen |
| *P. hymenina* | PUYB1 | 7 | 1 | 1.00 | 0.89 | Sexual | cyanolichen |
| ***Pseudocyphellaria*** | | | | | | | |
| *P. bartlettii* | TPB1 | 8 | 1 | 1.00 | 0.36 | Asexual | cyanolichen |
| *P. dubia* | TPB1 | 7 | 1 | 1.00 | 0.25 | Both | cyanolichen |
| *P. dubia* | RMB1 | 8 | 1 | 1.00 | 0.26 | Asexual | cyanolichen |
| *P. dubia* | PWB2 | 5 | 2 | 0.52 | 0.40 | Asexual | cyanolichen |
| *P. faveolata* | COB1 | 6 | 1 | 1.00 | 0.12 | Both | cephalolichen |
| *P. faveolata* | RMB1 | 6 | 3 | 0.39 | 0.06 | Asexual | cephalolichen |
| *P. freycinettii* | PWB1 | 7 | 4 | 0.31 | 0.80 | None | cephalolichen |
| *P. freycinettii* | PWB2 | 6 | 4 | 0.33 | 0.94 | None | cephalolichen |
| *P. gilva* | KKB1 | 7 | 2 | 0.76 | 0.14 | None | cyanolichen |
| *P. gilva* | TPB1 | 6 | 2 | 0.72 | 0.16 | Sexual | cyanolichen |
| *P. gilva* | RMB1 | 7 | 2 | 0.59 | 0.03 | Both | cyanolichen |
| *P. gilva* | PWB1 | 4 | 2 | 0.63 | 0.03 | Sexual | cyanolichen |
| *P. gilva* | PWB2 | 8 | 2 | 0.53 | 0.03 | Sexual | cyanolichen |
| *P. glabra* | COB1 | 5 | 3 | 0.44 | 0.38 | Asexual | cephalolichen |
| *P. glabra* | PUYB1 | 7 | 2 | 0.59 | 0.60 | Both | cephalolichen |
| *P. glabra* | PUYB2 | 7 | 2 | 0.51 | 0.57 | Asexual | cephalolichen |
| *P.* gr. *argyracea* | COB2 | 8 | 2 | 0.63 | 0.26 | Asexual | cyanolichen |
| *P.* gr. *argyracea* | PUYB2 | 4 | 2 | 0.63 | 0.23 | Asexual | cyanolichen |
| *P.* gr. *argyracea* | KKB1 | 8 | 1 | 1.00 | 0.16 | Both | cyanolichen |
| *P.* gr. *argyracea* | TPB1 | 8 | 2 | 0.78 | 0.21 | Both | cyanolichen |
| *P.* gr. *argyracea* | RMB1 | 8 | 2 | 0.78 | 0.13 | Asexual | cyanolichen |
| *P.* gr. *argyracea* | PWB1 | 8 | 2 | 0.78 | 0.27 | Asexual | cyanolichen |
| *P.* gr. *argyracea* | PWB2 | 8 | 2 | 0.63 | 0.06 | Both | cyanolichen |
| *P.* gr. *citrina* | COB1 | 5 | 1 | 1.00 | 0.09 | Asexual | cyanolichen |
| *P.* gr. *citrina* | COB2 | 8 | 1 | 1.00 | 0.15 | Asexual | cyanolichen |
| *P.* gr. *citrina* | PUYB2 | 7 | 2 | 0.51 | 0.39 | Asexual | cyanolichen |
| *P.* gr. *citrina* | KKB1 | 8 | 1 | 1.00 | 0.16 | Asexual | cyanolichen |
| *P.* gr. *citrina* | KKB2 | 4 | 1 | 1.00 | 0.18 | Asexual | cyanolichen |
| *P.* gr. *citrina* | TPB1 | 7 | 2 | 0.51 | 0.06 | Asexual | cyanolichen |
| *P.* gr. *citrina* | RMB1 | 7 | 2 | 0.51 | 0.00 | Asexual | cyanolichen |
| *P.* gr. *citrina* | PWB1 | 8 | 3 | 0.41 | 0.10 | Asexual | cyanolichen |
| *P.* gr. *citrina* | PWB2 | 8 | 3 | 0.47 | 0.06 | Asexual | cyanolichen |
| *P.* gr. *vaccina* | COB1 | 6 | 2 | 0.56 | 0.10 | Sexual | cephalolichen |
| *P.* gr. *vaccina* | KKB1 | 5 | 2 | 0.68 | 0.19 | Sexual | cephalolichen |
| *P.* gr. *vaccina* | KKB2 | 6 | 3 | 0.39 | 0.21 | Sexual | cephalolichen |
| *P.* gr. *vaccina* | TPB1 | 8 | 4 | 0.44 | 0.44 | Sexual | cephalolichen |
| *P.* gr. *vaccina* | PWB1 | 4 | 3 | 0.38 | 0.79 | Sexual | cephalolichen |
| *P.* gr. *vaccina* | PWB2 | 6 | 5 | 0.22 | 0.74 | Sexual | cephalolichen |
| *P. granulata* | COB1 | 4 | 2 | 0.63 | 0.03 | Asexual | cephalolichen |
| *P. granulata* | COB2 | 6 | 2 | 0.72 | 0.13 | Asexual | cephalolichen |
| *P. granulata* | KKB2 | 5 | 2 | 0.68 | 0.20 | Asexual | cephalolichen |
| *P. granulata* | TPB1 | 7 | 4 | 0.39 | 0.16 | Asexual | cephalolichen |
| *P. granulata* | RMB1 | 7 | 3 | 0.43 | 0.05 | Asexual | cephalolichen |
| *P. granulata* | PWB1 | 7 | 2 | 0.51 | 0.26 | Asexual | cephalolichen |
| *P. granulata* | PWB2 | 8 | 3 | 0.34 | 0.28 | Asexual | cephalolichen |
| *P. hirsuta* | COB2 | 5 | 1 | 1.00 | 0.07 | Sexual | cyanolichen |
| *P. hirsuta* | PUYB1 | 8 | 1 | 1.00 | 0.51 | Sexual | cyanolichen |
| *P. hirsuta* | KKB1 | 8 | 3 | 0.34 | 0.25 | Sexual | cyanolichen |
| *P. hirsuta* | KKB2 | 7 | 2 | 0.59 | 0.16 | Sexual | cyanolichen |
| *P. hirsuta* | TPB1 | 7 | 2 | 0.76 | 0.18 | Sexual | cyanolichen |
| *P. intricata* | KKB1 | 8 | 2 | 0.63 | 0.15 | Asexual | cyanolichen |
| *P. intricata* | KKB2 | 8 | 1 | 1.00 | 0.77 | Both | cyanolichen |
| *P. intricata* | PWB2 | 6 | 3 | 0.39 | 0.26 | Both | cyanolichen |
| *P. lechlerii* | TPB1 | 8 | 1 | 1.00 | 0.27 | Asexual | cyanolichen |
| *P. lechlerii* | RMB1 | 7 | 2 | 0.51 | 0.00 | Asexual | cyanolichen |
| *P. lechlerii* | PWB1 | 8 | 1 | 1.00 | 0.45 | Asexual | cyanolichen |
| *P. lechlerii* | PWB2 | 7 | 1 | 1.00 | 0.26 | Both | cyanolichen |
| *P. mallota* | KKB1 | 8 | 2 | 0.63 | 0.24 | Asexual | cyanolichen |
| *P. norvegica* | PUYB1 | 8 | 2 | 0.78 | 0.90 | Asexual | cyanolichen |
| *P. scabrosa* | TPB1 | 6 | 3 | 0.50 | 0.08 | Asexual | cyanolichen |
| *P. scabrosa* | RMB1 | 4 | 2 | 0.63 | 0.04 | Asexual | cyanolichen |
| *P. scabrosa* | PWB1 | 8 | 2 | 0.78 | 0.15 | Asexual | cyanolichen |
| *P. scabrosa* | PWB2 | 8 | 1 | 1.00 | 0.28 | Asexual | cyanolichen |
| ***Psoroma*** | | | | | | | |
| *P. asperellum* | KKB1 | 6 | 5 | 0.22 | 0.95 | Sexual | cephalolichen |
| ***Sticta*** | | | | | | | |
| *S. fuliginosa* | TPB1 | 8 | 1 | 1.00 | 0.92 | Asexual | cyanolichen |
| *S. hypochra* | COB1 | 5 | 2 | 0.68 | 0.69 | Both | cyanolichen |
| *S. hypochra* | COB2 | 4 | 1 | 1.00 | 0.84 | Asexual | cyanolichen |


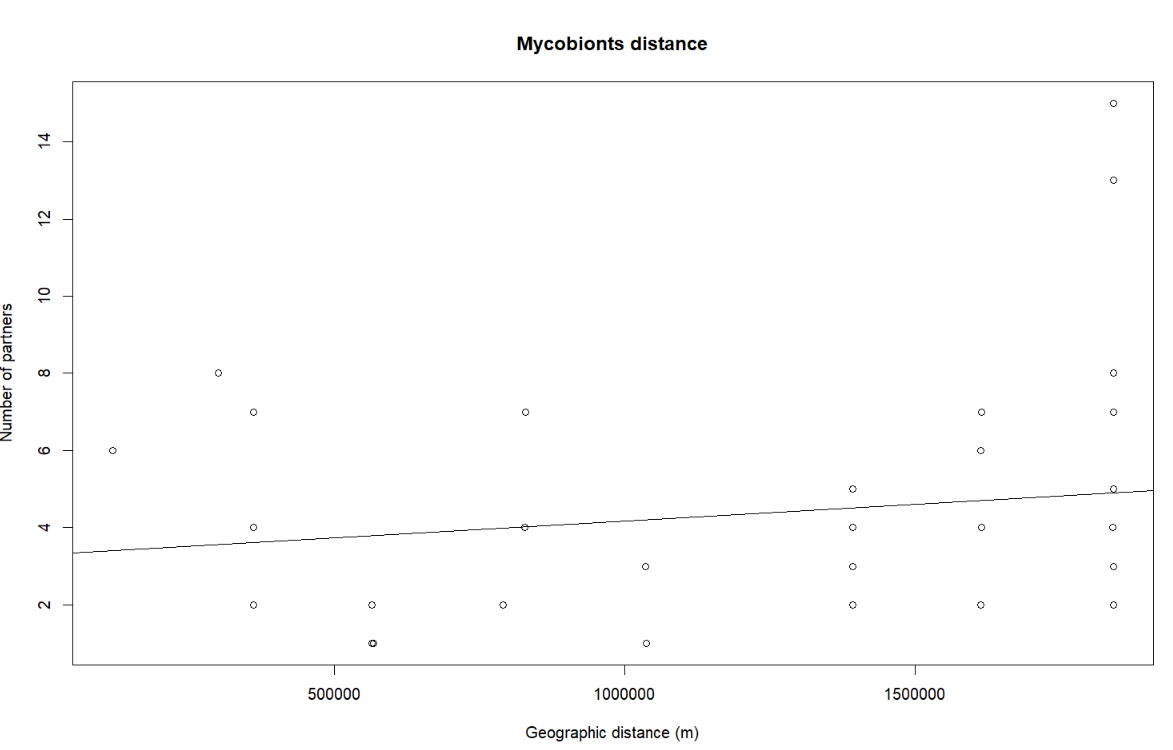


Appendix S14 Number of partners per species of mycobiont with at least 10 thalli along the latitudinal gradient with the geographic distance (m) (R^2^ = -0.0008, P = 0.33).

Appendix S15 Nostoc phylogroups and geographic distance (ordered by distance in km, followed by number of forest).

| **Nostoc** | **Thalli** | **Richness of partners** | **Distance**  **(km)** | **Number of forest** |
| --- | --- | --- | --- | --- |
| phylogroup_01 | 1 | 1 | 0 | 1 |
| phylogroup_06 | 1 | 1 | 0 | 1 |
| phylogroup_09 | 1 | 1 | 0 | 1 |
| phylogroup_15 | 1 | 1 | 0 | 1 |
| phylogroup_16 | 1 | 1 | 0 | 1 |
| phylogroup_18 | 1 | 1 | 0 | 1 |
| phylogroup_19 | 1 | 1 | 0 | 1 |
| phylogroup_20 | 1 | 1 | 0 | 1 |
| phylogroup_26 | 1 | 1 | 0 | 1 |
| phylogroup_27 | 1 | 1 | 0 | 1 |
| phylogroup_29 | 1 | 1 | 0 | 1 |
| phylogroup_30 | 1 | 1 | 0 | 1 |
| phylogroup_31 | 1 | 1 | 0 | 1 |
| phylogroup_33 | 1 | 1 | 0 | 1 |
| phylogroup_36 | 1 | 1 | 0 | 1 |
| phylogroup_41 | 1 | 1 | 0 | 1 |
| phylogroup_44 | 1 | 1 | 0 | 1 |
| phylogroup_45 | 1 | 1 | 0 | 1 |
| phylogroup_47 | 1 | 1 | 0 | 1 |
| phylogroup_55 | 1 | 1 | 0 | 1 |
| phylogroup_60 | 1 | 1 | 0 | 1 |
| phylogroup_61 | 1 | 1 | 0 | 1 |
| phylogroup_63 | 1 | 1 | 0 | 1 |
| phylogroup_64 | 1 | 1 | 0 | 1 |
| phylogroup_08 | 2 | 1 | 0 | 1 |
| phylogroup_32 | 2 | 1 | 0 | 1 |
| phylogroup_39 | 2 | 1 | 0 | 1 |
| phylogroup_58 | 2 | 1 | 0 | 1 |
| phylogroup_05 | 2 | 2 | 0 | 1 |
| phylogroup_28 | 2 | 2 | 0 | 1 |
| phylogroup_34 | 3 | 3 | 0 | 1 |
| phylogroup_24 | 9 | 2 | 1.65 | 2 |
| phylogroup_53 | 3 | 1 | 2.21 | 2 |
| phylogroup_11 | 10 | 3 | 2.21 | 2 |
| phylogroup_46 | 3 | 1 | 10.87 | 2 |
| phylogroup_25 | 2 | 1 | 118.49 | 2 |
| phylogroup_10 | 3 | 3 | 118.49 | 3 |
| phylogroup_50 | 2 | 1 | 243.20 | 2 |
| phylogroup_51 | 9 | 3 | 24320 | 3 |
| phylogroup_02 | 18 | 2 | 361.24 | 4 |
| phylogroup_56 | 5 | 3 | 565.13 | 2 |
| phylogroup_59 | 5 | 4 | 565.13 | 2 |
| phylogroup_57 | 4 | 2 | 565.61 | 3 |
| phylogroup_37 | 5 | 3 | 568.19 | 2 |
| phylogroup_40 | 21 | 1 | 568.19 | 3 |
| phylogroup_13 | 3 | 1 | 827.51 | 2 |
| phylogroup_17 | 3 | 2 | 827.78 | 2 |
| phylogroup_62 | 3 | 3 | 827.78 | 2 |
| phylogroup_14 | 5 | 3 | 1036.64 | 3 |
| phylogroup_07 | 23 | 7 | 1036.64 | 4 |
| phylogroup_54 | 19 | 5 | 1036.64 | 5 |
| phylogroup_48 | 28 | 8 | 1036.64 | 6 |
| phylogroup_49 | 25 | 1 | 1393.77 | 4 |
| phylogroup_22 | 39 | 9 | 1393.77 | 7 |
| phylogroup_35 | 66 | 17 | 1840.97 | 7 |
| phylogroup_52 | 27 | 10 | 1841.41 | 6 |
| phylogroup_03 | 8 | 4 | 1841.91 | 3 |
| phylogroup_21 | 30 | 12 | 1841.91 | 5 |
| phylogroup_04 | 24 | 8 | 1841.91 | 6 |
| phylogroup_43 | 30 | 7 | 1841.91 | 8 |
| phylogroup_42 | 216 | 27 | 1841.91 | 11 |
| phylogroup_38 | 418 | 47 | 1841.91 | 11 |
| phylogroup_23 | 2 | 2 | 1842.35 | 2 |
| phylogroup_12 | 13 | 5 | 1842.35 | 4 |

Appendix S16 Mycobiont species and geographic distance (ordered by distance in km, followed by number of forest).

| **Mycobionts** | **Thalli** | **Richness of partners** | ***Nostoc* phylogroup identity** | **Distance**  **(km)** | **Number of forests** |
| --- | --- | --- | --- | --- | --- |
| *Leciophysma sp. 1* | 1 | 1 | 19 | 0 | 1 |
| *Leptogium cochleatum* | 1 | 1 | 44 | 0 | 1 |
| *Leptogium sp. 1* | 1 | 1 | 4 | 0 | 1 |
| *Pannaria contorta* | 1 | 1 | 42 | 0 | 1 |
| *Pannaria isabellina* | 1 | 1 | 35 | 0 | 1 |
| *Parmeliella sp. 1* | 1 | 1 | 38 | 0 | 1 |
| *Pseudocyphellaria encoensis* | 1 | 1 | 42 | 0 | 1 |
| *Pseudocyphellaria sp1* | 1 | 1 | 11 | 0 | 1 |
| *Pseudocyphellaria divulsa* | 1 | 1 | 34 | 0 | 1 |
| *Psorophorus pholidotus* | 1 | 1 | 38 | 0 | 1 |
| *Pseudocyphellaria wandae* | 1 | 1 | 38 | 0 | 1 |
| *Sticta ainoae* | 1 | 1 | 30 | 0 | 1 |
| *Xanthopsoroma contextum* | 1 | 1 | 42 | 0 | 1 |
| *Xanthopsoroma soccatum* | 1 | 1 | 38 | 0 | 1 |
| *Pannaria sp1* | 2 | 1 | 52 | 0 | 1 |
| *Pannaria aff patagonica* | 2 | 1 | 52 | 0 | 1 |
| *Pseudocyphellaria nudata* | 2 | 1 | 38 | 0 | 1 |
| *Peltigera degenii* | 2 | 1 | 43 | 0 | 1 |
| *Pannaria arthroophylla* | 3 | 2 | 59, 64 | 0 | 1 |
| *Psoroma hypnorum* | 3 | 2 | 12, 14 | 0 | 1 |
| *Fuscopannaria mediterranea* | 4 | 1 | 35 | 0 | 1 |
| *Peltigera rufescens* | 4 | 1 | 43 | 0 | 1 |
| *Collema glaucophthalmum* | 4 | 3 | 37, 52, 56 | 0 | 1 |
| *Sticta caulescens* | 5 | 1 | 21 | 0 | 1 |
| *Leptogium patagonicum* | 5 | 2 | 04, 39 | 0 | 1 |
| *Pannaria aff implexa* | 5 | 2 | 52, 56 | 0 | 1 |
| *Pannaria byssoidea* | 7 | 2 | 35, 38 | 0 | 1 |
| *Leptogium aff. tenuissimum* | 7 | 6 | 07, 18, 20, 28, 37, 38 | 0 | 1 |
| *Psoroma polychinoides (=Santessoniella polychinoides)* | 3 | 1 | 53 | 2.21 | 2 |
| *Pseudocyphellaria coppinsii* | 7 | 3 | 35, 38, 52 | 2.21 | 2 |
| *Fuscopannaria sp. 1* | 3 | 2 | 38, 48 | 10.87 | 2 |
| *Nephroma kuehenmanii (+ N. microphyllum)* | 2 | 2 | 22, 38 | 118.49 | 2 |
| *Nephroma plumbeum* | 2 | 2 | 01, 38 | 118.49 | 2 |
| *Pseudocyphellaria valdiviana* | 7 | 4 | 21, 22, 27, 34 | 118.49 | 2 |
| *Nephroma analogicum (+ N. chubutense)* | 11 | 6 | 10, 21, 29, 34, 35, 52 | 118.49 | 3 |
| *Pseudocyphellaria flavicans* | 8 | 3 | 04, 21, 38 | 242.50 | 3 |
| *Leptogium azureum* | 3 | 2 | 23, 38 | 243.20 | 2 |
| *Pseudocyphellaria freycinetti* | 15 | 8 | 12, 14, 15, 24, 31, 33, 38, 42 | 300.33 | 3 |
| *Pseudocyphellaria berberina (+ P. coerulescens)* | 3 | 1 | 38 | 361.24 | 3 |
| *Peltigera polydactylon* | 5 | 1 | 2 | 361.24 | 3 |
| *Nephroma skottsbergii (+ N. papillosum)* | 20 | 7 | 08, 10, 21, 25, 35, 36, 38 | 361.24 | 4 |
| *Peltigera hymenina* | 20 | 2 | 02, 43 | 361.24 | 5 |
| *Pseudocyphellaria glabra* | 21 | 4 | 16, 21, 22, 38 | 361.24 | 5 |
| *Psoroma hirsutulum* | 2 | 2 | 05, 38 | 565.13 | 2 |
| *Pseudocyphellaria bartlettii* | 10 | 1 | 38 | 565.13 | 3 |
| *Pseudocyphellaria dubia* | 23 | 2 | 35, 42 | 565.61 | 4 |
| *Peltigera collina* | 21 | 1 | 40 | 568.19 | 3 |
| *Pseudocyphellaria mallota* | 12 | 2 | 38, 42 | 790.86 | 4 |
| *Leptogium laceroides* | 9 | 3 | 04, 12, 17 | 827.51 | 3 |
| *Leptogium valdivianum* | 12 | 4 | 12, 51, 52, 62 | 827.51 | 4 |
| *Nephroma pseudoparile* | 4 | 1 | 38 | 830.08 | 2 |
| *Peltigera membranacea* | 8 | 1 | 43 | 830.08 | 3 |
| *Psoroma asperellum* | 11 | 7 | 12, 13, 22, 28, 38, 45, 46 | 830.08 | 3 |
| *Peltigera canina* | 6 | 2 | 03, 43 | 1036.64 | 2 |
| *Pseudocyphellaria intricata* | 22 | 3 | 07, 38, 42 | 1036.64 | 3 |
| *Pseudocyphellaria gilva* | 34 | 3 | 35, 38, 42 | 1036.64 | 6 |
| *Sticta fuliginosa* | 12 | 1 | 54 | 1037.54 | 4 |
| *Sticta gr. weigelii* | 5 | 3 | 11, 38, 54 | 1152.41 | 3 |
| *Pseudocyphellaria piloselloides* | 6 | 1 | 38 | 1378.82 | 6 |
| *Parmeliella nigrata* | 4 | 2 | 38, 42 | 1393.77 | 3 |
| *Nephroma parile* | 28 | 3 | 35, 42, 49 | 1393.77 | 4 |
| *Pseudocyphellaria norvegica* | 13 | 4 | 11, 32, 38, 50 | 1393.77 | 5 |
| *Sticta hypochra* | 15 | 2 | 21, 22 | 1393.77 | 5 |
| *Leptogium decipiens* | 15 | 5 | 03, 04, 06, 37, 38 | 1393.77 | 5 |
| *Peltigera praetextata* | 4 | 1 | 43 | 1394.34 | 4 |
| *Pseudocyphellaria lechleri* | 32 | 2 | 38, 42 | 1613.61 | 6 |
| *Pseudocyphellaria obvoluta* | 37 | 6 | 21, 38, 42, 48, 57, 59 | 1613.73 | 8 |
| *Pannaria gr. Sphinctrina* | 20 | 6 | 21, 35, 42, 52, 58, 59 | 1614.08 | 5 |
| *Pseudocyphellaria guillemini* | 26 | 4 | 07, 35, 38, 42 | 1615.51 | 6 |
| *Collema flaccidum* | 18 | 7 | 07, 17, 22, 38, 51, 54, 62 | 1615.51 | 7 |
| *Pannaria farinosa* | 23 | 4 | 38, 42, 48, 63 | 1840.97 | 5 |
| *Leptogium meziensii* | 6 | 2 | 04, 43 | 1841.91 | 3 |
| *Pseudocyphellaria faveolata* | 17 | 3 | 38, 42, 48 | 1841.91 | 5 |
| *Parmeliella nigrocinta* | 18 | 3 | 35, 38, 42 | 1841.91 | 6 |
| *Pannaria pulverulacea* | 21 | 8 | 10, 21, 22, 38, 42, 52, 56, 59 | 1841.91 | 7 |
| *Pseudocyphellaria scabrosa* | 34 | 3 | 35, 38, 42 | 1841.91 | 7 |
| *Pseudocyphellaria gr. vaccina* | 36 | 13 | 03, 04, 07, 14, 21, 22, 23, 24, 38, 41, 42, 47, 57 | 1841.91 | 7 |
| *Pannaria pallida* | 23 | 2 | 38, 42 | 1841.91 | 8 |
| *Pseudocyphellaria granulata* | 45 | 7 | 04, 05, 38, 42, 48, 52, 54 | 1841.91 | 8 |
| *Pseudocyphellaria hirsuta* | 40 | 5 | 07, 35, 38, 42, 48 | 1841.91 | 9 |
| *Nephroma cellulosum* | 49 | 3 | 35, 38, 42 | 1841.91 | 10 |
| *Pseudocyphellaria gr. argyracea* | 57 | 2 | 38, 42 | 1841.91 | 10 |
| *Nephroma antarcticum* | 61 | 15 | 03, 07, 09, 21, 22, 26, 35, 38, 42, 48, 51, 54, 55, 60, 61 | 1841.91 | 11 |
| *Pseudocyphellaria gr. citrina* | 67 | 3 | 35, 38, 42 | 1841.91 | 11 |
| *Fuscopannaria minor* | 13 | 3 | 38, 48, 62 | 1842.35 | 5 |

Appendix S17 Beta diversity between forests and the regional scale. Comparisons are made within species for each the forest the species is present with the regional scale. β_tot_: total beta diversity, reflecting both species replacement and loss/gain (β_total_ = β_repl_ + β_rich_). β_repl_: beta diversity explained by replacement of species alone. β_rich_: beta diversity explained by species loss/gain (richness differences) alone. Description: turnover strategy following Ventre Lespiaucq et al., 2021.

| **Regional** | **Local** | **β_tot_** | **Description** |
| --- | --- | --- | --- |
| *Collema flaccidum* |  |  |  |
|  | TPB1 | 0.71 | Nested turnover, variable richness |
| *Crocodia guilleminii* |  |  |  |
|  | PUYB2 | 0.75 | Nested turnover, variable richness |
|  | TPB1 | 0.50 | Nested turnover, variable richness |
|  | RMB1 | 0.50 | Nested turnover, variable richness |
| *Cyanisticta obvoluta* |  |  |  |
|  | KKB2 | 0.67 | Nested turnover, variable richness |
|  | TPB1 | 0.67 | Nested turnover, variable richness |
|  | RMB1 | 0.67 | Nested turnover, variable richness |
|  | PWB1 | 0.33 | Nested turnover, variable richness |
|  | PWB2 | 0.33 | Nested turnover, variable richness |
| *Fuscopannaria mediterranea* |  |  |  |
|  | RMB1 | 0.67 | Nested turnover, variable richness |
|  | PWB1 | 0.33 | Nested turnover, variable richness |
| *Leptogium decipiens* |  |  |  |
|  | COB1 | 0.60 | Nested turnover, variable richness |
|  | COB2 | 0.60 | Nested turnover, variable richness |
| *Leptogium valdivianum* |  |  |  |
|  | COB1 | 0.50 | Nested turnover, variable richness |
| *Nephroma analogicum* |  |  |  |
|  | PUYB2 | 0.33 | Nested turnover, variable richness |
| *Nephroma antarcticum* |  |  |  |
|  | COB2 | 0.80 | Nested turnover, variable richness |
|  | PUYB1 | 0.80 | Nested turnover, variable richness |
|  | KKB1 | 0.80 | Nested turnover, variable richness |
|  | KKB2 | 0.73 | Nested turnover, variable richness |
|  | TPB1 | 0.73 | Nested turnover, variable richness |
|  | RMB1 | 0.80 | Nested turnover, variable richness |
|  | PWB1 | 0.93 | Nested turnover, variable richness |
|  | PWB2 | 0.80 | Nested turnover, variable richness |
| *Nephroma cellulosum* |  |  |  |
|  | COB1 | 0.67 | Nested turnover, variable richness |
|  | COB2 | 0.33 | Nested turnover, variable richness |
|  | PUYB1 | 0.67 | Nested turnover, variable richness |
|  | PUYB2 | 0.33 | Nested turnover, variable richness |
|  | KKB1 | 0.67 | Nested turnover, variable richness |
|  | TPB1 | 0.33 | Nested turnover, variable richness |
|  | RMB1 | 0.33 | Nested turnover, variable richness |
| *Nephroma pseudoparile* |  |  |  |
|  | COB1 | 0.67 | Nested turnover, variable richness |
|  | COB2 | 0.67 | Nested turnover, variable richness |
|  | KKB2 | 0.67 | Nested turnover, variable richness |
|  | TPB1 | 0.00 | No turnover, constant richness |
| *Nephroma skottsbergii* |  |  |  |
|  | PUYB1 | 0.71 | Nested turnover, variable richness |
|  | PUYB2 | 0.29 | Nested turnover, variable richness |
| *Pannaria farinosa* |  |  |  |
|  | TPB1 | 0.75 | Nested turnover, variable richness |
|  | RMB1 | 0.25 | Nested turnover, variable richness |
|  | PWB1 | 0.25 | Nested turnover, variable richness |
|  | PWB2 | 0.75 | Nested turnover, variable richness |
| *Pannaria gr. sphinctrina* |  |  |  |
|  | TPB1 | 0.83 | Nested turnover, variable richness |
|  | RMB1 | 0.67 | Nested turnover, variable richness |
|  | PWB1 | 0.33 | Nested turnover, variable richness |
| *Pannaria pallida* |  |  |  |
|  | RMB1 | 0.33 | Nested turnover, variable richness |
| *Pannaria pulverulacea* |  |  |  |
|  | PUYB1 | 0.50 | Nested turnover, variable richness |
|  | PUYB2 | 0.75 | Nested turnover, variable richness |
| *Parmeliella nigrocinta* |  |  |  |
|  | COB2 | 0.67 | Nested turnover, variable richness |
|  | PWB1 | 0.33 | Nested turnover, variable richness |
| *Peltigera collina* |  |  |  |
|  | KKB1 | 0.00 | No turnover, constant richness |
|  | KKB2 | 0.00 | No turnover, constant richness |
|  | TPB1 | 0.00 | No turnover, constant richness |
| *Peltigera hymenina* |  |  |  |
|  | COB1 | 0.00 | No turnover, constant richness |
|  | PUYB1 | 0.50 | Nested turnover, variable richness |
| *Pseudocyphellaria bartlettii* |  |  |  |
|  | TPB1 | 0.00 | No turnover, constant richness |
| *Pseudocyphellaria dubia* |  |  |  |
|  | TPB1 | 0.50 | Nested turnover, variable richness |
|  | RMB1 | 0.50 | Nested turnover, variable richness |
|  | PWB2 | 0.00 | No turnover, constant richness |
| *Pseudocyphellaria faveolata* |  |  |  |
|  | COB1 | 0.67 | Nested turnover, variable richness |
|  | RMB1 | 0.00 | No turnover, constant richness |
| *Pseudocyphellaria freycinettii* |  |  |  |
|  | PWB1 | 0.50 | Nested turnover, variable richness |
|  | PWB2 | 0.50 | Nested turnover, variable richness |
| *Pseudocyphellaria gilva* |  |  |  |
|  | KKB1 | 0.33 | Nested turnover, variable richness |
|  | TPB1 | 0.33 | Nested turnover, variable richness |
|  | RMB1 | 0.33 | Nested turnover, variable richness |
|  | PWB1 | 0.33 | Nested turnover, variable richness |
|  | PWB2 | 0.33 | Nested turnover, variable richness |
| *Pseudocyphellaria glabra* |  |  |  |
|  | COB1 | 0.25 | Nested turnover, variable richness |
|  | PUYB1 | 0.50 | Nested turnover, variable richness |
|  | PUYB2 | 0.50 | Nested turnover, variable richness |
| *Pseudocyphellaria* gr. *argyracea* |  |  |  |
|  | COB2 | 0.00 | No turnover, constant richness |
|  | PUYB2 | 0.00 | No turnover, constant richness |
|  | KKB1 | 0.50 | Nested turnover, variable richness |
|  | TPB1 | 0.00 | No turnover, constant richness |
|  | RMB1 | 0.00 | No turnover, constant richness |
|  | PWB1 | 0.00 | No turnover, constant richness |
|  | PWB2 | 0.00 | No turnover, constant richness |
| *Pseudocyphellaria* gr. *citrina* |  |  |  |
|  | COB1 | 0.67 | Nested turnover, variable richness |
|  | COB2 | 0.67 | Nested turnover, variable richness |
|  | PUYB2 | 0.33 | Nested turnover, variable richness |
|  | KKB1 | 0.67 | Nested turnover, variable richness |
|  | KKB2 | 0.67 | Nested turnover, variable richness |
|  | TPB1 | 0.33 | Nested turnover, variable richness |
|  | RMB1 | 0.33 | Nested turnover, variable richness |
|  | PWB1 | 0.00 | No turnover, constant richness |
|  | PWB2 | 0.00 | No turnover, constant richness |
| *Pseudocyphellaria* gr. *vaccina* |  |  |  |
|  | COB1 | 0.85 | Nested turnover, variable richness |
|  | KKB1 | 0.85 | Nested turnover, variable richness |
|  | KKB2 | 0.77 | Nested turnover, variable richness |
|  | TPB1 | 0.69 | Nested turnover, variable richness |
|  | PWB1 | 0.77 | Nested turnover, variable richness |
|  | PWB2 | 0.62 | Nested turnover, variable richness |
| *Pseudocyphellaria granulata* |  |  |  |
|  | COB1 | 0.71 | Nested turnover, variable richness |
|  | COB2 | 0.71 | Nested turnover, variable richness |
|  | KKB2 | 0.71 | Nested turnover, variable richness |
|  | TPB1 | 0.43 | Nested turnover, variable richness |
|  | RMB1 | 0.57 | Nested turnover, variable richness |
|  | PWB1 | 0.71 | Nested turnover, variable richness |
|  | PWB2 | 0.57 | Nested turnover, variable richness |
| *Pseudocyphellaria hirsuta* |  |  |  |
|  | COB2 | 0.80 | Nested turnover, variable richness |
|  | PUYB1 | 0.80 | Nested turnover, variable richness |
|  | KKB1 | 0.40 | Nested turnover, variable richness |
|  | KKB2 | 0.60 | Nested turnover, variable richness |
|  | TPB1 | 0.60 | Nested turnover, variable richness |
| *Pseudocyphellaria intricata* |  |  |  |
|  | KKB1 | 0.33 | Nested turnover, variable richness |
|  | KKB2 | 0.67 | Nested turnover, variable richness |
|  | PWB2 | 0.00 | No turnover, constant richness |
| *Pseudocyphellaria lechlerii* |  |  |  |
|  | TPB1 | 0.50 | Nested turnover, variable richness |
|  | RMB1 | 0.00 | No turnover, constant richness |
|  | PWB1 | 0.50 | Nested turnover, variable richness |
|  | PWB2 | 0.50 | Nested turnover, variable richness |
| *Pseudocyphellaria mallota* |  |  |  |
|  | KKB1 | 0.00 | No turnover, constant richness |
| *Pseudocyphellaria norvegica* |  |  |  |
|  | PUYB1 | 0.50 | Nested turnover, variable richness |
| *Pseudocyphellaria scabrosa* |  |  |  |
|  | TPB1 | 0.00 | No turnover, constant richness |
|  | RMB1 | 0.33 | Nested turnover, variable richness |
|  | PWB1 | 0.33 | Nested turnover, variable richness |
|  | PWB2 | 0.67 | Nested turnover, variable richness |
| *Psoroma asperellum* |  |  |  |
|  | KKB1 | 0.29 | Nested turnover, variable richness |
| *Sticta fuliginosa* |  |  |  |
|  | TPB1 | 0.00 | No turnover, constant richness |
| *Sticta hypochra* |  |  |  |
|  | COB1 | 0.00 | No turnover, constant richness |
|  | COB2 | 0.50 | Nested turnover, variable richness |

Appendix S18 Beta diversity between forests. Comparisons are made within species forest by forests in each the forest the species is present. β_tot_: total beta diversity, reflecting both species replacement and loss/gain (β_total_ = β_repl_ + β_rich_). β_repl_: beta diversity explained by replacement of species alone. β_rich_: beta diversity explained by species loss/gain (richness differences) alone. Description: turnover strategy following Ventre Lespiaucq et al., 2021.

| **Specie** | **Forest 1** | **Forest 2** | **β_tot_** | **β_repl_** | **β_rich_** | **Description** |
| --- | --- | --- | --- | --- | --- | --- |
| *Crocodia guilleminii* |  |  |  |  |  |  |
|  | PUYB2 | TPB1 | 1.00 | 0.67 | 0.33 | Total replacement, variable richness |
|  | PUYB2 | RMB1 | 0.50 | 0.00 | 0.50 | Nested turnover, variable richness |
|  | TPB1 | RMB1 | 0.67 | 0.67 | 0.00 | Partial replacement, constant richness |
| *Cyanisticta obvoluta* |  |  |  |  |  |  |
|  | KKB2 | TPB1 | 0.67 | 0.67 | 0.00 | Partial replacement, constant richness |
|  | KKB2 | RMB1 | 0.67 | 0.67 | 0.00 | Partial replacement, constant richness |
|  | KKB2 | PWB1 | 0.50 | 0.00 | 0.50 | Nested turnover, variable richness |
|  | KKB2 | PWB2 | 0.80 | 0.40 | 0.40 | Partial replacement, variable richness |
|  | TPB1 | RMB1 | 0.67 | 0.67 | 0.00 | Partial replacement, constant richness |
|  | TPB1 | PWB1 | 0.50 | 0.00 | 0.50 | Nested turnover, variable richness |
|  | TPB1 | PWB2 | 0.50 | 0.00 | 0.50 | Nested turnover, variable richness |
|  | RMB1 | PWB1 | 0.80 | 0.40 | 0.40 | Partial replacement, variable richness |
|  | RMB1 | PWB2 | 0.50 | 0.00 | 0.50 | Nested turnover, variable richness |
|  | PWB1 | PWB2 | 0.67 | 0.67 | 0.00 | Partial replacement, constant richness |
| *Fuscopannaria mediterranea* |  |  |  |  |  |  |
|  | COB1 | PWB1 | 0.50 | 0.00 | 0.50 | Nested turnover, variable richness |
| *Leptogium decipiens* |  |  |  |  |  |  |
|  | COB2 | COB2 | 0.67 | 0.67 | 0.00 | Partial replacement, constant richness |
| *Nephroma antarcticum* |  |  |  |  |  |  |
|  | COB2 | PUYB1 | 0.80 | 0.80 | 0.00 | Partial replacement, constant richness |
|  | COB2 | KKB1 | 0.80 | 0.80 | 0.00 | Partial replacement, constant richness |
|  | COB2 | KKB2 | 0.83 | 0.67 | 0.17 | Partial replacement, variable richness |
|  | COB2 | TPB1 | 0.83 | 0.67 | 0.17 | Partial replacement, variable richness |
|  | COB2 | RMB1 | 0.80 | 0.80 | 0.00 | Partial replacement, constant richness |
|  | COB2 | PWB1 | 1.00 | 0.50 | 0.50 | Total replacement, variable richness |
|  | COB2 | PWB2 | 1.00 | 1.00 | 0.00 | Total replacement, constant richness |
|  | PUYB1 | KKB1 | 0.80 | 0.80 | 0.00 | Partial replacement, constant richness |
|  | PUYB1 | KKB2 | 0.83 | 0.67 | 0.17 | Partial replacement, variable richness |
|  | PUYB1 | TPB1 | 0.83 | 0.67 | 0.17 | Partial replacement, variable richness |
|  | PUYB1 | RMB1 | 0.80 | 0.80 | 0.00 | Partial replacement, constant richness |
|  | PUYB1 | PWB1 | 1.00 | 0.50 | 0.50 | Total replacement, variable richness |
|  | PUYB1 | PWB2 | 1.00 | 1.00 | 0.00 | Total replacement, constant richness |
|  | KKB1 | KKB2 | 0.60 | 0.40 | 0.20 | Partial replacement, variable richness |
|  | KKB1 | TPB1 | 0.60 | 0.40 | 0.20 | Partial replacement, variable richness |
|  | KKB1 | RMB1 | 0.50 | 0.50 | 0.00 | Partial replacement, constant richness |
|  | KKB1 | PWB1 | 1.00 | 0.50 | 0.50 | Total replacement, variable richness |
|  | KKB1 | PWB2 | 1.00 | 1.00 | 0.00 | Total replacement, constant richness |
|  | KKB2 | TPB1 | 0.67 | 0.67 | 0.00 | Partial replacement, constant richness |
|  | KKB2 | RMB1 | 0.60 | 0.40 | 0.20 | Partial replacement, variable richness |
|  | KKB2 | PWB1 | 1.00 | 0.40 | 0.60 | Total replacement, variable richness |
|  | KKB2 | PWB2 | 1.00 | 0.86 | 0.14 | Total replacement, variable richness |
|  | TPB1 | RMB1 | 0.25 | 0.00 | 0.25 | Nested turnover, variable richness |
|  | TPB1 | PWB1 | 0.75 | 0.00 | 0.75 | Nested turnover, variable richness |
|  | TPB1 | PWB2 | 0.83 | 0.67 | 0.17 | Partial replacement, variable richness |
|  | RMB1 | PWB1 | 0.67 | 0.00 | 0.67 | Nested turnover, variable richness |
|  | RMB1 | PWB2 | 0.80 | 0.80 | 0.00 | Partial replacement, constant richness |
|  | PWB1 | PWB2 | 0.67 | 0.00 | 0.67 | Nested turnover, variable richness |
| *Nephroma cellulosum* |  |  |  |  |  |  |
|  | COB1 | COB2 | 0.50 | 0.00 | 0.50 | Nested turnover, variable richness |
|  | COB1 | PUYB1 | 1.00 | 1.00 | 0.00 | Total replacement, constant richness |
|  | COB1 | PUYB2 | 0.50 | 0.00 | 0.50 | Nested turnover, variable richness |
|  | COB1 | KKB1 | 0.00 | 0.00 | 0.00 | No turnover, constant richness |
|  | COB1 | TPB1 | 1.00 | 0.67 | 0.33 | Total replacement, variable richness |
|  | COB1 | RMB1 | 0.50 | 0.00 | 0.50 | Nested turnover, variable richness |
|  | COB2 | PUYB1 | 0.50 | 0.00 | 0.50 | Nested turnover, variable richness |
|  | COB2 | PUYB2 | 0.00 | 0.00 | 0.00 | No turnover, constant richness |
|  | COB2 | KKB1 | 0.50 | 0.00 | 0.50 | Nested turnover, variable richness |
|  | COB2 | TPB1 | 0.67 | 0.67 | 0.00 | Partial replacement, constant richness |
|  | COB2 | RMB1 | 0.67 | 0.67 | 0.00 | Partial replacement, constant richness |
|  | PUYB1 | PUYB2 | 0.50 | 0.00 | 0.50 | Nested turnover, variable richness |
|  | PUYB1 | KKB1 | 1.00 | 1.00 | 0.00 | Total replacement, constant richness |
|  | PUYB1 | TPB1 | 0.50 | 0.00 | 0.50 | Nested turnover, variable richness |
|  | PUYB1 | RMB1 | 1.00 | 0.67 | 0.33 | Total replacement, variable richness |
|  | PUYB2 | KKB1 | 0.50 | 0.00 | 0.50 | Nested turnover, variable richness |
|  | PUYB2 | TPB1 | 0.67 | 0.67 | 0.00 | Partial replacement, constant richness |
|  | PUYB2 | RMB1 | 0.67 | 0.67 | 0.00 | Partial replacement, constant richness |
|  | KKB1 | TPB1 | 1.00 | 0.67 | 0.33 | Total replacement, variable richness |
|  | KKB1 | RMB1 | 0.50 | 0.00 | 0.50 | Nested turnover, variable richness |
|  | TPB1 | RMB1 | 0.67 | 0.67 | 0.00 | Partial replacement, constant richness |
| *Nephroma pseudoparile* |  |  |  |  |  |  |
|  | COB1 | COB2 | 0.00 | 0.00 | 0.00 | No turnover, constant richness |
|  | COB1 | KKB2 | 0.00 | 0.00 | 0.00 | No turnover, constant richness |
|  | COB1 | TPB1 | 0.67 | 0.00 | 0.67 | Nested turnover, variable richness |
|  | COB2 | KKB2 | 0.00 | 0.00 | 0.00 | No turnover, constant richness |
|  | COB2 | TPB1 | 0.67 | 0.00 | 0.67 | Nested turnover, variable richness |
|  | KKB2 | TPB1 | 0.67 | 0.00 | 0.67 | Nested turnover, variable richness |
| *Nephroma skottsbergii* |  |  |  |  |  |  |
|  | PUYB1 | PUYB2 | 0.83 | 0.33 | 0.50 | Partial replacement, variable richness |
| *Pannaria farinosa* |  |  |  |  |  |  |
|  | TPB1 | RMB1 | 0.67 | 0.00 | 0.67 | Nested turnover, variable richness |
|  | TPB1 | PWB1 | 0.67 | 0.00 | 0.67 | Nested turnover, variable richness |
|  | TPB1 | PWB2 | 0.00 | 0.00 | 0.00 | No turnover, constant richness |
|  | RMB1 | PWB1 | 0.50 | 0.50 | 0.00 | Partial replacement, constant richness |
|  | RMB1 | PWB2 | 0.67 | 0.00 | 0.67 | Nested turnover, variable richness |
|  | PWB1 | PWB2 | 0.67 | 0.00 | 0.67 | Nested turnover, variable richness |
| *Pannaria gr. sphinctrina* |  |  |  |  |  |  |
|  | TPB1 | RMB1 | 0.50 | 0.00 | 0.50 | Nested turnover, variable richness |
|  | TPB1 | PWB1 | 0.75 | 0.00 | 0.75 | Nested turnover, variable richness |
|  | RMB1 | PWB1 | 0.50 | 0.00 | 0.50 | Nested turnover, variable richness |
| *Pannaria pulverulacea* |  |  |  |  |  |  |
|  | PUYB1 | PUYB2 | 0.80 | 0.40 | 0.40 | Partial replacement, variable richness |
| *Parmeliella nigrocinta* |  |  |  |  |  |  |
|  | COB2 | PWB1 | 1.00 | 0.67 | 0.33 | Total replacement, variable richness |
| *Peltigera collina* |  |  |  |  |  |  |
|  | KKB1 | KKB2 | 0.00 | 0.00 | 0.00 | No turnover, constant richness |
|  | KKB1 | TPB1 | 0.00 | 0.00 | 0.00 | No turnover, constant richness |
|  | KKB2 | TPB1 | 0.00 | 0.00 | 0.00 | No turnover, constant richness |
| *Peltigera hymenina* |  |  |  |  |  |  |
|  | COB1 | PUYB1 | 0.50 | 0.00 | 0.50 | Nested turnover, variable richness |
| *Pseudocyphellaria dubia* |  |  |  |  |  |  |
|  | TPB1 | RMB1 | 0.00 | 0.00 | 0.00 | No turnover, constant richness |
|  | TPB1 | PWB2 | 0.50 | 0.00 | 0.50 | Nested turnover, variable richness |
|  | RMB1 | PWB2 | 0.50 | 0.00 | 0.50 | Nested turnover, variable richness |
| *Pseudocyphellaria faveolata* |  |  |  |  |  |  |
|  | COB1 | RMB1 | 0.67 | 0.00 | 0.67 | Nested turnover, variable richness |
| *Pseudocyphellaria freycinettii* |  |  |  |  |  |  |
|  | PWB1 | PWB2 | 0.67 | 0.67 | 0.00 | Partial replacement, constant richness |
| *Pseudocyphellaria glabra* |  |  |  |  |  |  |
|  | COB1 | PUYB1 | 0.33 | 0.00 | 0.33 | Nested turnover, variable richness |
|  | COB1 | PUYB2 | 0.33 | 0.00 | 0.33 | Nested turnover, variable richness |
|  | PUYB1 | PUYB2 | 0.00 | 0.00 | 0.00 | No turnover, constant richness |
| *Pseudocyphellaria gilva* |  |  |  |  |  |  |
|  | KKB1 | TPB1 | 0.67 | 0.67 | 0.00 | Partial replacement, constant richness |
|  | KKB1 | RMB1 | 0.00 | 0.00 | 0.00 | No turnover, constant richness |
|  | KKB1 | PWB1 | 0.00 | 0.00 | 0.00 | No turnover, constant richness |
|  | KKB1 | PWB2 | 0.00 | 0.00 | 0.00 | No turnover, constant richness |
|  | TPB1 | RMB1 | 0.67 | 0.67 | 0.00 | Partial replacement, constant richness |
|  | TPB1 | PWB1 | 0.67 | 0.67 | 0.00 | Partial replacement, constant richness |
|  | TPB1 | PWB2 | 0.67 | 0.67 | 0.00 | Partial replacement, constant richness |
|  | RMB1 | PWB1 | 0.00 | 0.00 | 0.00 | No turnover, constant richness |
|  | RMB1 | PWB2 | 0.00 | 0.00 | 0.00 | No turnover, constant richness |
|  | PWB1 | PWB2 | 0.00 | 0.00 | 0.00 | No turnover, constant richness |
| *Pseudocyphellaria* gr. *argyracea* |  |  |  |  |  |  |
|  | COB2 | PUYB2 | 0.00 | 0.00 | 0.00 | No turnover, constant richness |
|  | COB2 | KKB1 | 0.50 | 0.00 | 0.50 | Nested turnover, variable richness |
|  | COB2 | TPB1 | 0.00 | 0.00 | 0.00 | No turnover, constant richness |
|  | COB2 | RMB1 | 0.00 | 0.00 | 0.00 | No turnover, constant richness |
|  | COB2 | PWB1 | 0.00 | 0.00 | 0.00 | No turnover, constant richness |
|  | COB2 | PWB2 | 0.00 | 0.00 | 0.00 | No turnover, constant richness |
|  | PUYB2 | KKB1 | 0.50 | 0.00 | 0.50 | Nested turnover, variable richness |
|  | PUYB2 | TPB1 | 0.00 | 0.00 | 0.00 | No turnover, constant richness |
|  | PUYB2 | RMB1 | 0.00 | 0.00 | 0.00 | No turnover, constant richness |
|  | PUYB2 | PWB1 | 0.00 | 0.00 | 0.00 | No turnover, constant richness |
|  | PUYB2 | PWB2 | 0.00 | 0.00 | 0.00 | No turnover, constant richness |
|  | KKB1 | TPB1 | 0.50 | 0.00 | 0.50 | Nested turnover, variable richness |
|  | KKB1 | RMB1 | 0.50 | 0.00 | 0.50 | Nested turnover, variable richness |
|  | KKB1 | PWB1 | 0.50 | 0.00 | 0.50 | Nested turnover, variable richness |
|  | KKB1 | PWB2 | 0.50 | 0.00 | 0.50 | Nested turnover, variable richness |
|  | TPB1 | RMB1 | 0.00 | 0.00 | 0.00 | No turnover, constant richness |
|  | TPB1 | PWB1 | 0.00 | 0.00 | 0.00 | No turnover, constant richness |
|  | TPB1 | PWB2 | 0.00 | 0.00 | 0.00 | No turnover, constant richness |
|  | RMB1 | PWB1 | 0.00 | 0.00 | 0.00 | No turnover, constant richness |
|  | RMB1 | PWB2 | 0.00 | 0.00 | 0.00 | No turnover, constant richness |
|  | PWB1 | PWB2 | 0.00 | 0.00 | 0.00 | No turnover, constant richness |
| *Pseudocyphellaria* gr. *citrina* |  |  |  |  |  |  |
|  | COB1 | COB2 | 0.00 | 0.00 | 0.00 | No turnover, constant richness |
|  | COB1 | PUYB2 | 0.50 | 0.00 | 0.50 | Nested turnover, variable richness |
|  | COB1 | KKB1 | 0.00 | 0.00 | 0.00 | No turnover, constant richness |
|  | COB1 | KKB2 | 0.00 | 0.00 | 0.00 | No turnover, constant richness |
|  | COB1 | TPB1 | 0.50 | 0.00 | 0.50 | Nested turnover, variable richness |
|  | COB1 | RMB1 | 0.50 | 0.00 | 0.50 | Nested turnover, variable richness |
|  | COB1 | PWB1 | 0.67 | 0.00 | 0.67 | Nested turnover, variable richness |
|  | COB1 | PWB2 | 0.67 | 0.00 | 0.67 | Nested turnover, variable richness |
|  | COB2 | PUYB2 | 0.50 | 0.00 | 0.50 | Nested turnover, variable richness |
|  | COB2 | KKB1 | 0.00 | 0.00 | 0.00 | No turnover, constant richness |
|  | COB2 | KKB2 | 0.00 | 0.00 | 0.00 | No turnover, constant richness |
|  | COB2 | TPB1 | 0.50 | 0.00 | 0.50 | Nested turnover, variable richness |
|  | COB2 | RMB1 | 0.50 | 0.00 | 0.50 | Nested turnover, variable richness |
|  | COB2 | PWB1 | 0.67 | 0.00 | 0.67 | Nested turnover, variable richness |
|  | COB2 | PWB2 | 0.67 | 0.00 | 0.67 | Nested turnover, variable richness |
|  | PUYB2 | KKB1 | 0.50 | 0.00 | 0.50 | Nested turnover, variable richness |
|  | PUYB2 | KKB2 | 0.50 | 0.00 | 0.50 | Nested turnover, variable richness |
|  | PUYB2 | TPB1 | 0.00 | 0.00 | 0.00 | No turnover, constant richness |
|  | PUYB2 | RMB1 | 0.00 | 0.00 | 0.00 | No turnover, constant richness |
|  | PUYB2 | PWB1 | 0.33 | 0.00 | 0.33 | Nested turnover, variable richness |
|  | PUYB2 | PWB2 | 0.33 | 0.00 | 0.33 | Nested turnover, variable richness |
|  | KKB1 | KKB2 | 0.00 | 0.00 | 0.00 | No turnover, constant richness |
|  | KKB1 | TPB1 | 0.50 | 0.00 | 0.50 | Nested turnover, variable richness |
|  | KKB1 | RMB1 | 0.50 | 0.00 | 0.50 | Nested turnover, variable richness |
|  | KKB1 | PWB1 | 0.67 | 0.00 | 0.67 | Nested turnover, variable richness |
|  | KKB1 | PWB2 | 0.67 | 0.00 | 0.67 | Nested turnover, variable richness |
|  | KKB2 | TPB1 | 0.50 | 0.00 | 0.50 | Nested turnover, variable richness |
|  | KKB2 | RMB1 | 0.50 | 0.00 | 0.50 | Nested turnover, variable richness |
|  | KKB2 | PWB1 | 0.67 | 0.00 | 0.67 | Nested turnover, variable richness |
|  | KKB2 | PWB2 | 0.67 | 0.00 | 0.67 | Nested turnover, variable richness |
|  | TPB1 | RMB1 | 0.00 | 0.00 | 0.00 | No turnover, constant richness |
|  | TPB1 | PWB1 | 0.33 | 0.00 | 0.33 | Nested turnover, variable richness |
|  | TPB1 | PWB2 | 0.33 | 0.00 | 0.33 | Nested turnover, variable richness |
|  | RMB1 | PWB1 | 0.33 | 0.00 | 0.33 | Nested turnover, variable richness |
|  | RMB1 | PWB2 | 0.33 | 0.00 | 0.33 | Nested turnover, variable richness |
|  | PWB1 | PWB2 | 0.00 | 0.00 | 0.00 | No turnover, constant richness |
| *Pseudocyphellaria* gr. *vaccina* |  |  |  |  |  |  |
|  | COB1 | KKB1 | 0.67 | 0.67 | 0.00 | Partial replacement, constant richness |
|  | COB1 | KKB2 | 0.33 | 0.00 | 0.33 | Nested turnover, variable richness |
|  | COB1 | TPB1 | 1.00 | 0.67 | 0.33 | Total replacement, variable richness |
|  | COB1 | PWB1 | 1.00 | 0.80 | 0.20 | Total replacement, variable richness |
|  | COB1 | PWB2 | 1.00 | 0.57 | 0.43 | Total replacement, variable richness |
|  | KKB1 | KKB2 | 0.75 | 0.50 | 0.25 | Partial replacement, variable richness |
|  | KKB1 | TPB1 | 1.00 | 0.67 | 0.33 | Total replacement, variable richness |
|  | KKB1 | PWB1 | 1.00 | 0.80 | 0.20 | Total replacement, variable richness |
|  | KKB1 | PWB2 | 1.00 | 0.57 | 0.43 | Total replacement, variable richness |
|  | KKB2 | TPB1 | 1.00 | 0.86 | 0.14 | Total replacement, variable richness |
|  | KKB2 | PWB1 | 1.00 | 1.00 | 0.00 | Total replacement, constant richness |
|  | KKB2 | PWB2 | 1.00 | 0.75 | 0.25 | Total replacement, variable richness |
|  | TPB1 | PWB1 | 0.83 | 0.67 | 0.17 | Partial replacement, variable richness |
|  | TPB1 | PWB2 | 0.88 | 0.75 | 0.13 | Partial replacement, variable richness |
|  | PWB1 | PWB2 | 0.86 | 0.57 | 0.29 | Partial replacement, variable richness |
| *Pseudocyphellaria granulata* |  |  |  |  |  |  |
|  | COB1 | COB2 | 0.67 | 0.67 | 0.00 | Partial replacement, constant richness |
|  | COB1 | KKB2 | 0.67 | 0.67 | 0.00 | Partial replacement, constant richness |
|  | COB1 | TPB1 | 0.80 | 0.40 | 0.40 | Partial replacement, variable richness |
|  | COB1 | RMB1 | 0.75 | 0.50 | 0.25 | Partial replacement, variable richness |
|  | COB1 | PWB1 | 1.00 | 1.00 | 0.00 | Total replacement, constant richness |
|  | COB1 | PWB2 | 0.75 | 0.50 | 0.25 | Partial replacement, variable richness |
|  | COB2 | KKB2 | 0.67 | 0.67 | 0.00 | Partial replacement, constant richness |
|  | COB2 | TPB1 | 0.80 | 0.40 | 0.40 | Partial replacement, variable richness |
|  | COB2 | RMB1 | 0.75 | 0.50 | 0.25 | Partial replacement, variable richness |
|  | COB2 | PWB1 | 1.00 | 1.00 | 0.00 | Total replacement, constant richness |
|  | COB2 | PWB2 | 0.75 | 0.50 | 0.25 | Partial replacement, variable richness |
|  | KKB2 | TPB1 | 0.80 | 0.40 | 0.40 | Partial replacement, variable richness |
|  | KKB2 | RMB1 | 0.33 | 0.00 | 0.33 | Nested turnover, variable richness |
|  | KKB2 | PWB1 | 0.67 | 0.67 | 0.00 | Partial replacement, constant richness |
|  | KKB2 | PWB2 | 0.33 | 0.00 | 0.33 | Nested turnover, variable richness |
|  | TPB1 | RMB1 | 0.60 | 0.40 | 0.20 | Partial replacement, variable richness |
|  | TPB1 | PWB1 | 0.80 | 0.40 | 0.40 | Partial replacement, variable richness |
|  | TPB1 | PWB2 | 0.60 | 0.40 | 0.20 | Partial replacement, variable richness |
|  | RMB1 | PWB1 | 0.33 | 0.00 | 0.33 | Nested turnover, variable richness |
|  | RMB1 | PWB2 | 0.00 | 0.00 | 0.00 | No turnover, constant richness |
|  | PWB1 | PWB2 | 0.33 | 0.00 | 0.33 | Nested turnover, variable richness |
| *Pseudocyphellaria hirsuta* |  |  |  |  |  |  |
|  | COB2 | PUYB1 | 1.00 | 1.00 | 0.00 | Total replacement, constant richness |
|  | COB2 | KKB1 | 0.67 | 0.00 | 0.67 | Nested turnover, variable richness |
|  | COB2 | KKB2 | 0.50 | 0.00 | 0.50 | Nested turnover, variable richness |
|  | COB2 | TPB1 | 1.00 | 0.67 | 0.33 | Total replacement, variable richness |
|  | PUYB1 | KKB1 | 1.00 | 0.50 | 0.50 | Total replacement, variable richness |
|  | PUYB1 | KKB2 | 1.00 | 0.67 | 0.33 | Total replacement, variable richness |
|  | PUYB1 | TPB1 | 0.50 | 0.00 | 0.50 | Nested turnover, variable richness |
|  | KKB1 | KKB2 | 0.33 | 0.00 | 0.33 | Nested turnover, variable richness |
|  | KKB1 | TPB1 | 1.00 | 0.80 | 0.20 | Total replacement, variable richness |
|  | KKB2 | TPB1 | 1.00 | 1.00 | 0.00 | Total replacement, constant richness |
| *Pseudocyphellaria intricata* |  |  |  |  |  |  |
|  | KKB1 | KKB2 | 0.50 | 0.00 | 0.50 | Nested turnover, variable richness |
|  | KKB1 | PWB2 | 0.33 | 0.00 | 0.33 | Nested turnover, variable richness |
|  | KKB2 | PWB2 | 0.67 | 0.00 | 0.67 | Nested turnover, variable richness |
| *Pseudocyphellaria lechlerii* |  |  |  |  |  |  |
|  | TPB1 | RMB1 | 0.50 | 0.00 | 0.50 | Nested turnover, variable richness |
|  | TPB1 | PWB1 | 1.00 | 1.00 | 0.00 | Total replacement, constant richness |
|  | TPB1 | PWB2 | 1.00 | 1.00 | 0.00 | Total replacement, constant richness |
|  | RMB1 | PWB1 | 0.50 | 0.00 | 0.50 | Nested turnover, variable richness |
|  | RMB1 | PWB2 | 0.50 | 0.00 | 0.50 | Nested turnover, variable richness |
|  | PWB1 | PWB2 | 0.00 | 0.00 | 0.00 | No turnover, constant richness |
| *Pseudocyphellaria scabrosa* |  |  |  |  |  |  |
|  | TPB1 | RMB1 | 0.33 | 0.00 | 0.33 | Nested turnover, variable richness |
|  | TPB1 | PWB1 | 0.33 | 0.00 | 0.33 | Nested turnover, variable richness |
|  | TPB1 | PWB2 | 0.67 | 0.00 | 0.67 | Nested turnover, variable richness |
|  | RMB1 | PWB1 | 0.00 | 0.00 | 0.00 | No turnover, constant richness |
|  | RMB1 | PWB2 | 0.50 | 0.00 | 0.50 | Nested turnover, variable richness |
|  | PWB1 | PWB2 | 0.50 | 0.00 | 0.50 | Nested turnover, variable richness |
| *Sticta hypochra* |  |  |  |  |  |  |
|  | COB1 | COB2 | 0.50 | 0.00 | 0.50 | Nested turnover, variable richness |
